# SupporTive Care At Home Research (STAHR) for patients with advanced cancer: Protocol for a cluster non-randomized controlled trial

**DOI:** 10.1371/journal.pone.0302011

**Published:** 2024-05-13

**Authors:** Dong-Wook Lee, Sun Young Lee, Shin Hye Yoo, Kyae Hyung Kim, Min-Sun Kim, Jeongmi Shin, In-Young Hwang, In Gyu Hwang, Sun Kyung Baek, Do yeun Kim, Yu Jung Kim, Beodeul Kang, Joongyub Lee, Belong Cho

**Affiliations:** 1 Department of Occupational and Environmental Medicine, Inha University Hospital, Inha University, Incheon, Republic of Korea; 2 Public Healthcare Center, Seoul National University Hospital, Seoul, Republic of Korea; 3 Department of Human System Medicine, Seoul National University College of Medicine, Seoul, Republic of Korea; 4 Center for Palliative Care and Clinical Ethics, Seoul National University Hospital, Seoul, Republic of Korea; 5 Department of Internal Medicine, Chung-Ang University Hospital, Seoul, Republic of Korea; 6 Department of Internal Medicine, Kyung Hee University Hospital, Seoul, Republic of Korea; 7 Department of Internal Medicine, Dongguk University Ilsan Hospital, Goyang, Republic of Korea; 8 Department of Internal Medicine, Seoul National University Bundang Hospital, Seongnam, Republic of Korea; 9 Department of Internal Medicine, Bundang CHA Hospital, Seongnam, Republic of Korea; 10 Department of Preventive Medicine, Seoul National University College of Medicine, Seoul, Republic of Korea; 11 Institute on Aging, Seoul National University College of Medicine, Seoul, Republic of Korea; 12 Department of Human Systems Medicine, Seoul National University College of Medicine, Seoul, Republic of Korea; University of Maribor, SLOVENIA

## Abstract

Advancements in the treatment and management of patients with cancer have extended their survival period. To honor such patients’ desire to live in their own homes, home-based supportive care programs have become an important medical practice. This study aims to investigate the effects of a multidimensional and integrated home-based supportive care program on patients with advanced cancer. SupporTive Care At Home Research is a cluster non-randomized controlled trial for patients with advanced cancer. This study tests the effects of the home-based supportive care program we developed versus standard oncology care. The home-based supportive care program is based on a specialized home-based medical team approach that includes (1) initial assessment and education for patients and their family caregivers, (2) home visits by nurses, (3) biweekly regular check-ups/evaluation and management, (4) telephone communication via a daytime access line, and (5) monthly multidisciplinary team meetings. The primary outcome measure is unplanned hospitalization within 6 months following enrollment. Healthcare service use; quality of life; pain and symptom control; emotional status; satisfaction with services; end-of-life care; advance planning; family caregivers’ quality of life, care burden, and preparedness for caregiving; and medical expenses will be surveyed. We plan to recruit a total of 396 patients with advanced cancer from six institutions. Patients recruited from three institutions will constitute the intervention group, whereas those recruited from the other three institutions will comprise the control group.

## Introduction

Advancements in medicine and the development of anti-cancer drugs and medical devices have improved the survival rates of patients with advanced cancer since the 1970s [1]. Although in many cases the cancer is not cured, a greater number of severely ill patients under treatment are surviving for longer periods. Patients with advanced cancer usually need to be hospitalized to help manage their distressing symptoms; however, a hospital stay is typically an unwanted experience for them [2]. Nevertheless, such patients need frequent hospitalizations and readmissions against their wish [3, 4]. Despite the increasing interest and investment in cancer treatment, there is an obvious lack of medical care for patients with advanced cancer.

Patients in the advanced stage of cancer desire to live at home, accept the inevitable, and prepare for their death [5]. Although they need medical services for their daily lives, social and medical problems lead to having restricted access to the necessary services outside of hospitals [6]. Home-based supportive care (HbSC) was developed to manage adverse reactions during cancer treatment and to respond to the needs and will of patients with advanced cancer. HbSC is reported to increase patients’ quality of life (QoL) and unplanned hospital visits [7–10]. Subsequently, the scope and target of HbSC has grown substantially worldwide [11–13].

HbSC for patients with advanced cancer with complicated chronic conditions can be used not only for unmet medical needs but also for allocation of medical resources. Patients with advanced cancer experience frequent hospitalization that is avoidable with HbSC [14]. Furthermore, long-term hospital stays in tertiary general hospitals reduce the number of available acute care beds, which may prevent many patients who need acute care from undergoing proper treatment. In South Korea, cancer-related emergency room (ER) visits comprised 5.5% of total ER visits from 2017 to 2019; hence, improving cancer care at the pre-hospital level with the help of HbSC programs may be necessary to solve ER overcrowding [15].

As HbSC programs for patients receiving supportive and palliative cancer care have become a common medical practice, related research has also been reported. For example, home-based intervention improved rehospitalization and survival rates in severely ill patients compared to conventional hospitalization and outpatient service [16]. Additionally, a recent study found HbSC to be more effective in controlling symptoms in patients with cancer than conventional care [17]. However, the effect on unplanned hospital visits has not been thoroughly investigated. In 1998, a systematic review concluded that the effectiveness of comprehensive home care programs remains unclear [18]. Another systematic review in 2016 also noted a lack of controlled clinical trials for HbSC in patients with advanced cancer [19].

SupporTive Care At Home Research (STAHR) is a cluster non-randomized controlled trial that aims to investigate the effects of home-based multidimensional and integrated supportive care for patients with advanced cancer with mobility difficulties and their family caregivers on reducing unplanned rehospitalization within six months.

## Methods and analysis

### Study design

A cluster non-randomized controlled trial design was chosen for this study for the following reasons. First, the HbSC programs for patients with advanced cancer that we designed are only available in a very limited number of hospitals. As there is a lack of adequate fee-for-service mechanisms for HbSC within the health insurance system, only a few hospitals with a stronger interest in providing public services operate small HbSC teams. Although the study’s funding was sufficient to support the expenses for a small number of additional healthcare professionals, there were only three hospitals that were suitable for the HbSC program intervention, and these were already operating their own HbSC teams. Second, within these three hospitals, using a randomization and blinding approach was not feasible, as it could lead to expectation bias on the part of the researchers. Third, among the hospitals categorized as tertiary general hospitals and that were similar in size and level of care, those that expressed interest in the HbSC program and decided to participate in this study were included as a cluster, and a separate control group was recruited. Thus, as we were unable to establish HbSC service teams for all hospitals for a randomized controlled study, we designed a non-randomized controlled trial using comparable hospitals; the hospitals in the intervention group will provide services to all study participants in their institutions.

The target group of this study is patients with advanced cancer undergoing active anti-cancer treatment with limited life expectancy and mobility difficulties. For this study, we aim to recruit a total of 396 patients with cancer from six institutions (66 participants from each institution; 198 each for the control and intervention groups). Participants for the intervention group will be recruited from Seoul National University Hospital, Chung-Ang University Hospital, and Dongguk University Ilsan Hospital. Participants for the control group will be recruited from Kyung Hee University Medical Center, CHA Bundang Medical Center, and Seoul National University Bundang Hospital. The intervention group will be provided with specialized HbSC until one year after the registration of the study. The control group will receive standard oncology care. Including unplanned hospital visits, the primary outcome of the study, outcome variables will be compared between the intervention and control groups at six months from enrollment.

**Fig 1:**
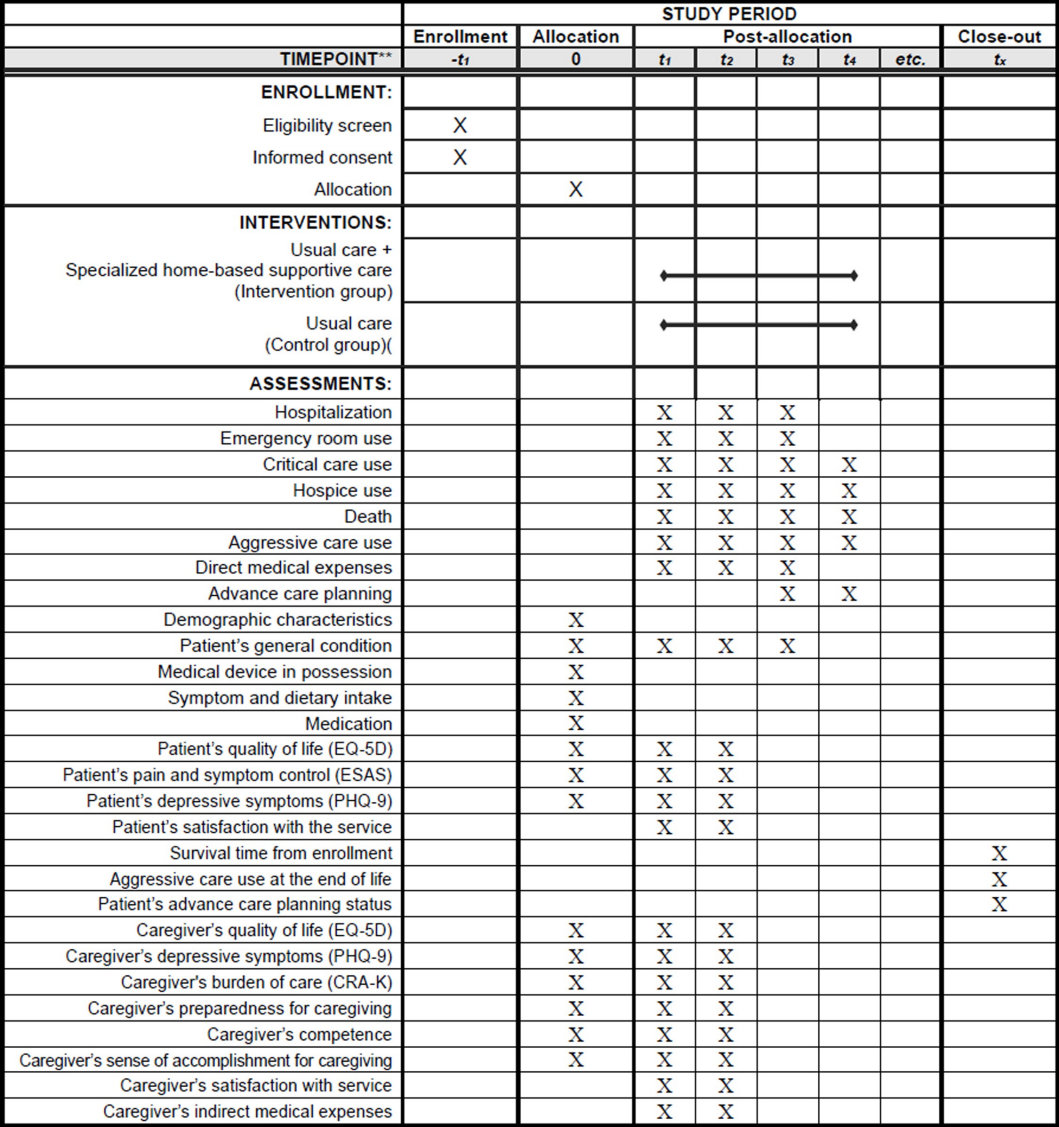
Timeline of recruitment, allocation, and assessments.

**Fig 2:**
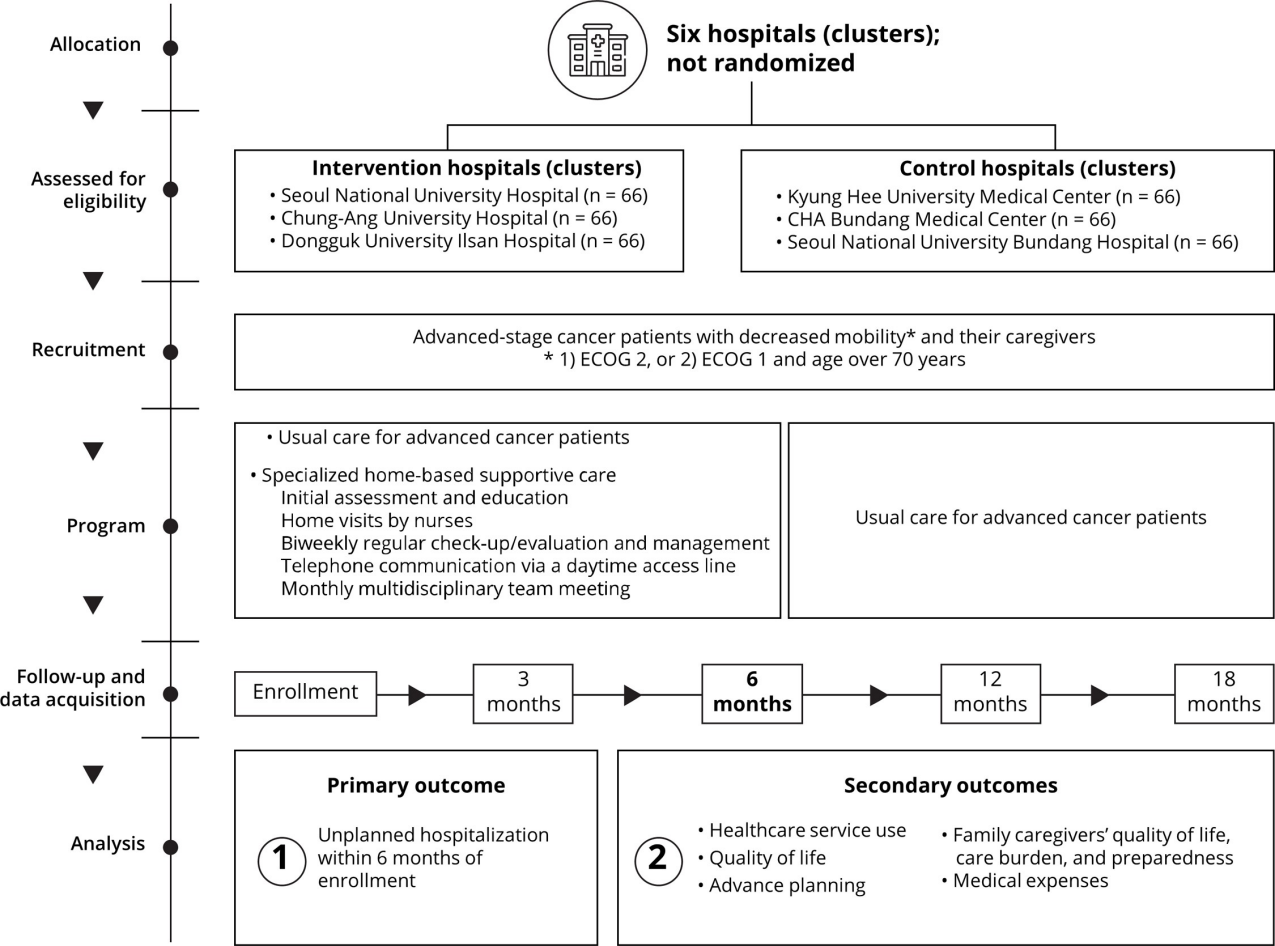
Schematic illustration of study design.

Fig 1 shows the timeline of recruitment, allocation, and assessments, and Fig 2 depicts the schematic structure of the study. This study began on July 25, 2022, and will be conducted until December 31, 2026. This trial protocol is registered with clinicaltrials.gov (NCT05636384) and follows the Standard Protocol Items: Recommendations for Intervention Trials reporting guidelines [20].

### Informed consent

Researchers will obtain written informed consent from all individuals in the study. Participants will sign an informed consent form.

### Inclusion/exclusion criteria

Both the patient and their family caregivers must meet the following criteria to be considered for this research. Inclusion criteria for patients are as follows: is a person who (1) has an advanced-stage solid cancer diagnosis (ICD-10 code C00-C70) and is receiving or planning to receive cancer treatment; (2) meets one of the following conditions: (a) has been evaluated as having Eastern Cooperative Oncology Group (ECOG) performance status 2 or (b) has been evaluated as having ECOG performance status 1 and is over 65 years of age [21]; (3) wishes to stay at home (in South Korea, patients who need supportive care are usually hospitalized in long-term care hospitals because national insurance covers the cost and there is a lack of supportive care in cancer treatment hospitals [22]); (4) has a family caregiver cohabiting with the patient or visiting the patient’s home three times or more per week; and (5) wishes to participate in the research. Inclusion criteria for family caregivers are as follows: (1) is a family member of the patient (“family” refers to the patient’s spouse [including a common-law partner], descendants and their spouses, siblings and their spouses, and other close relatives and their spouses) and (2) a person who meets one of the following conditions: (a) lives with the patient (member of the patient’s household) or (b) does not live with the patient but visits the patient’s home three or more times a week; (c) wants the patient to stay at home; (d) can communicate easily with medical staff via telephone without any difficulties; and (e) wishes to participate in the research.

Patients will be excluded from the study if they (1) cannot speak, hear, or read Korean; (2) are judged by a medical doctor to be unfit to take part in this research due to extremely poor health; (3) reside outside the range that can be visited by the respective medical institution; (4) are already receiving hospice service; and (5) are under the age of 19. Exclusion criteria for family caregivers are as follows: is a person who (1) cannot speak, hear, or read Korean; (2) is judged by a medical doctor to be unfit to take part in this research due to extremely poor health; and (3) is under the age of 19.

### Intervention

**Fig 3:**
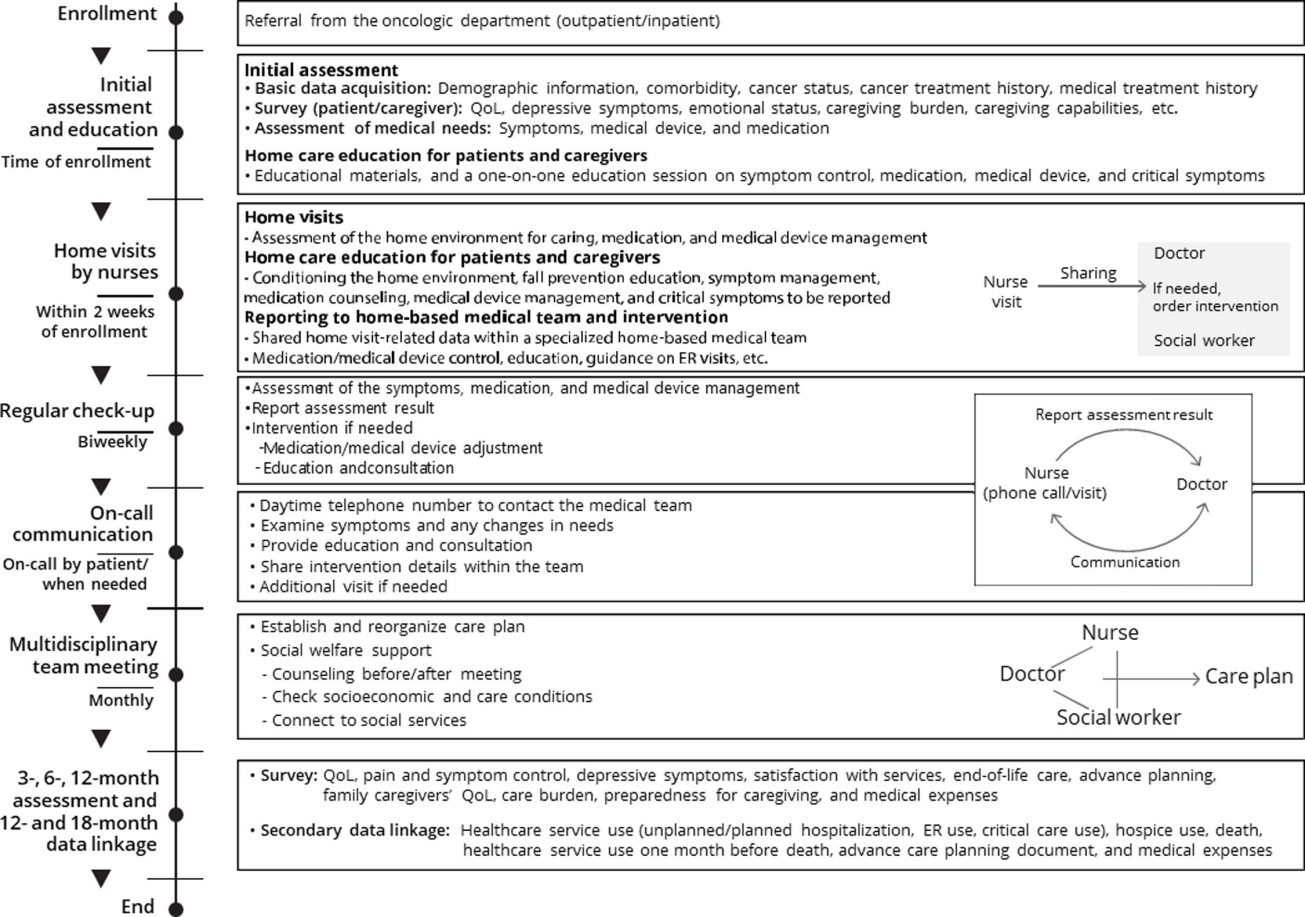
Intervention program of STAHR study.

The HbSC intervention program for the STAHR study (Fig 3) was developed by a multidisciplinary expert group based on reviews of relevant literature [23–36], as well as focus group interviews with five cancer patients, four caregivers of cancer patients, and healthcare professionals to explore their unmet medical needs. This program includes (1) initial assessment and education for patients and their family caregivers, (2) home visits by nurses, (3) biweekly regular check-ups/evaluations and management, (4) telephone communication via a daytime access line, and (5) monthly multidisciplinary team meetings. The institutions for intervention will establish home-based medical teams comprising doctors, nurses, and social workers. The doctors are home-based care specialists and/or hematologist-oncologists. The nurses are dedicated home-based medical intervention nurses who are newly recruited and have undergone home-based medical intervention training for more than three months. Professional medical staff provided the educational program, consisting of lectures to facilitate the understanding of the palliative care and symptoms of patients with advanced cancer and HbSC program simulation training. Patients enrolled in the intervention cluster (institution) will receive specialized home-based medical care based on a specialized home-based medical team approach, initial assessment and education, initial home visits and education, a multidisciplinary team meeting, and regular assessment after the initial visits.

#### Initial assessment and education for patients and their family caregivers

When patients are enrolled in the study, an initial assessment will be conducted immediately. Nurses will assess patients regarding demographic information, past medical history, current medical condition, QoL, symptoms, emotional status, care burden, care ability, pharmaceutical history, symptom control status, and medical device usage. Patients will receive the educational materials and have a one-on-one education session on symptom control (15 min), medication counseling, medical device management, and critical symptoms that must be reported to nurses.

#### Home visits by nurses

Nurses on the team will visit the enrolled patient’s home within two weeks following the initial assessment. They will make an appointment for a home visit when both the patient and their family caregiver are available. Next, they will assess the home environment for care, medication, and medical device management of patients. The patients will be educated regarding conditioning the home environment, fall prevention education, symptom management, medication counseling, medical device management, and critical symptoms that need to be reported to nurses.

#### Biweekly regular check-up/evaluation and management

The nurses will contact the patients biweekly via telephone and conduct assessments of their symptom status. The assessment includes symptom status, medication adherence, medical device management, home environment for caring, and any needs of patients and family caregivers. The team doctor will review the assessment, and if necessary, decide to provide medication/medical device adjustment, education, and guidance on visiting the ER. If the patient assessment is insufficient via telephone or in-person patient education is required, the nurses will visit the patients’ home.

#### Telephone communication via a daytime access line

A daytime telephone number to contact the home medical team nurse between 9 am and 5 pm on weekdays will be provided to the study participants. If the patient has any questions or concerns, they can leave a message for the nurse via an access line. The nurse will call the patient and their caregivers to consult about their symptoms, events, or any concerns about the patient’s status.

#### Monthly multidisciplinary team meeting

A multidisciplinary team meeting of specialized home-based medical teams will be convened once a month. Team meeting agendas are as follows: sharing information on patients who are enrolled in the study, establishing a care plan for participating patients, and regularly reassessing the patients to adjust the care plan. The care plan includes the objectives of home care based on the assessment, symptom management, additional medical needs at home, and reported critical symptoms of patients.

### Control

Patients enrolled in the control cluster (institution) will receive educational materials. Usual oncology care for patients with advanced cancer will not be restricted to these patients.

### Primary and secondary outcomes

The primary outcome of this study is unplanned hospitalization within six months following enrollment. Unplanned hospitalization is defined as one of the following: (a) admission to the enrolled hospital to treat unexpected symptoms or medical conditions (not including planned admission for chemotherapy or admission for planned surgery or intervention); (b) admission to a hospital other than the enrolled hospital; and (c) admission to a nursing hospital for a duration of fewer than four weeks.

Secondary outcomes will be selected across healthcare service use; QoL; pain and symptom control; emotional status; satisfaction with services; advance planning; aggressive care use at the end of life; family caregivers’ QoL, care burden, and preparedness for caregiving; and medical expenses. The patient healthcare service use domain will include hospitalization, ER visits, and healthcare use for severe diseases (intensive care unit [ICU] admission, ventilator treatment). The patient end-of-life care domain will comprise ICU admission one month before death and ventilator treatment. Advance care planning status concerns the existence of an advance directive. The patient QoL and satisfaction with services domain will include QoL (EuroQol-5 [EQ5D] index score), pain and symptom control (Modified Edmonton Symptom Assessment System [ESAS]), depressive symptoms (Patient Health Questionnaire-9 [PHQ-9]), and satisfaction with services. The family caregiver domain will encompass QoL (EQ-5D), care burden (the Korean version of the Caregiver Reaction Assessment scale [CRA-K]), depressive symptoms (PHQ-9), ability to perform care items, and satisfaction with services. Finally, the expenses domain will include medical and care expenses.

### Outcome measurement

For those patients who agree to secondary data linkage and are enrolled in the intervention study, National Health Insurance Service data will be shared after more than 12 months of enrollment (Table 1). The relevant data will be used to investigate information on healthcare service use and direct medical cost up to 12 months after their enrollment. Data will be evaluated at 12 months to recalculate and utilize medical use and cost information at three, six, and 12 months. At 18 months or more after enrollment, all patients who are enrolled in the intervention study and provide consent for data sharing will be investigated for information on death, hospice use, healthcare service use before death, and advance care planning status. The items to be investigated through sharing data from the National Health Insurance Service are listed in Table 1.

**Table 1:** Secondary data linkage plan for primary and secondary outcomes.

Item	Outcome measurement timing (from the time of enrollment)			
	3 months	6 months	12 months	18 months
***Hospitalization*** Hospitalization date, discharge date Hospitalization route (outpatient, emergency room) Hospitalization results (discharge, death)	○	○	○	
***Emergency room use*** Date (year month date) Result (discharge, hospitalization, death)	○	○	○	
***Critical care use*** Date of ICU hospitalization (day) Date of ventilator support (day) Number of CPR attempts	○	○	○	○
***Hospice use*** Hospitalization date, discharge date	○	○	○	○
***Death*** Vital status Date of death Cause of death	○	○	○	○
***Aggressive care use 1 month before death*** Date of ICU hospitalization (day) Date of ventilator support (day) Number of CPR attempts	○	○	○	○
***Direct medical expenses (KRW)***	○	○	○	
***Advance care planning status*** Advance directives Physician orders for life-sustaining treatment			○	○

The intervention and control groups will undergo an interview evaluation by the researchers at the time of enrollment, and the questionnaire will be evaluated at the time of enrollment, at three, six, and 12 months (Table 2). The questionnaire evaluation will be conducted when the patients and caregivers visit the institutions for outpatient treatment or hospitalization within one month before or after the time of enrollment. The nurse will visit the outpatient or inpatient ward to directly evaluate the patients. If the patient and family caregiver do not visit the hospital due to the absence of outpatient or hospitalization schedules within a month before or after the time of enrollment, the nurse will contact the patient and family caregiver by phone or mail/email to complete the questionnaire evaluation.

**Table 2:** Outcomes and time points for investigation.

Item	Outcome measurement timing (from the time of enrollment)				
	0 mo.	3 mo.	6 mo.	12 mo.	Death
Demographic characteristics^#^	○				
Patient’s general condition	○	○	○	○	
Medical device in possession	○				
Symptom and dietary intake	●				
Medication	●				
Patient’s quality of life (EQ-5D)	○	○	●		
Patient’s pain and symptom control (ESAS)	○	○	●		
Patient’s depressive symptoms (PHQ-9)	○	○	●		
Patient’s satisfaction with the service		●	●		
Survival time from enrollment					○
Aggressive care use at the end of life (ICU admission, chemotherapy, and ventilator treatment)					○
Patient’s advance care planning status					○
Caregiver’s quality of life (EQ-5D)	○	○	●		
Caregiver’s depressive symptoms (PHQ-9)	○	○	●		
Caregiver’s burden of care (CRA-K)	○	○	●		
Caregiver’s preparedness for caregiving	○	○	●		
Caregiver’s competence	○	○	●		
Caregiver’s sense of accomplishment for caregiving	○	○	●		
Caregiver’s satisfaction with service		●	●		
Caregiver’s indirect medical expenses		○	○^*^		

^#^ Patient: age, sex, residence, medical security, education, marriage, religion, employment, monthly income, residence, welfare service and disability type, private insurance, household characteristics, family and household composition, care provision status; Caregiver: age, sex, relationship with patient, co-residence with patient, education, marriage, religion, employment, patient care experience

○ Both intervention/control groups, ● Only intervention group

^*^ Conducted in the control group only when consent was provided at 3 months after enrollment for evaluation at 6 months.

### Safety and monitoring

The intervention group will be provided with a home-based medical program, comprising education and counseling services. No side effects or risks associated with the intervention are expected. The control group will receive the same existing medical service and is expected to have no side effects or risks associated with participation in the study. Investigators in participating hospitals will have regular meetings once a month to audit trial conduct.

### Data management

We have established the following data management plan. Data from the six hospitals will be collected using a web-based case report form (CRF), which is based on the data management program iCReaT (http://icreat.cdc.go.kr) and developed by the Korea National Institute of Health. Data will be entered at each hospital by trained nurses. Validation of the entered data will be performed at Seoul National University Hospital. Every missing, extreme, and unsound input item will be submitted to every investigator as a question or a comment via iCReat. Each investigator will be expected to respond to the question and fix any errors found.

### Sample size calculation

The number of samples of the home-based program to be evaluated in this study is based on a dichotomous variable, which is the primary endpoint, to secure a stable and sufficient number of patients for power. The primary endpoint of this study is unplanned hospitalization within six months following enrollment. This study aims to show differences in the effects by comparing the odds ratio based on the proportion of unplanned hospitalizations in the intervention and control groups. Although there is no previous intervention study with a similar design conducted outside of Korea, the difference in pre- and post-intervention changes between the intervention and control groups is approximately 20% (20% less in the intervention group) based on the literature review. The number of institutions in both groups is three, and parameters will be estimated using a hierarchical mixed model to compensate for the cluster effects of the study participants. Based on the described analysis method, a power of 80.0%, significance level of 0.05 to control for type 1 error, and 20% difference in the number of unplanned hospitalizations by intervention will be used to calculate the sample size. The proportion of unplanned hospitalization in the control group was assumed to be 0.3, after referencing a related study [37]. In a ratio of 1:1 for participants in each group, the minimal sample size is 336 patients in total (56 for each institution) with assumption of intracluster correlation 0.015. Assuming a maximum dropout rate of 15%, the final number of enrolled study participants is expected to be 396 patients (66 patients for each hospital). Intracluster correlation was estimated using the analysis of variance estimation method according to similar studies found through the literature review. The calculation formula is as follows [38]:

$$\hat{ICC}=\frac{MSB-MSW}{MSB+\left(n_{A}-1\right)MSW}$$

$$n_{A}=\frac{1}{k-1}\left(N-\frac{\sum n_{i}^{2}}{N}\right),MSB=\frac{1}{k-1}\left(\sum \frac{Z_{I}^{2}}{n_{i}}-\frac{\sum z_{i}^{2}}{N}\right),MSW=\frac{1}{N-k}\left(\sum Z_{i}-\sum \frac{Z_{i}^{2}}{n_{i}}\right),$$

where *k* is the number of clusters, and *N* is the total number of study participants. *n*_*i*_ and *Z*_*i*_ are the number of participants in group *i* and number of patients with endpoints in group *i*, respectively. Incidence rates by group were extracted from Chen et al. and applied in this study [37].

Sample size calculation was performed with PASS 2022 (NCSS, LLC. Kaysville, UT). A total of three institutions each will participate in the intervention (Seoul National University Hospital, Chung-Ang University Hospital, Dongguk University Ilsan Hospital) and control groups (Kyunghee University Hospital, Bundan CHA hospital, and Bundang Seoul University Hospital).

### Statistical analysis

The intention-to-treat principle that only considers the initial classification of the intervention will be used. The mean proportion of unplanned hospitalization will be compared between the intervention and control groups for the primary outcome. The mean of continuous variables and proportion of dichotomous variables will be compared between the two groups for the secondary outcome. A hierarchical mixed model will be used to evaluate intra-group correlation to compensate for clusters of patients in the participating institutions. Survival after enrollment will be compared using the Kaplan–Meier curve and Cox proportional hazard model.

### Ethics approval

All methods were carried out in accordance with relevant guidelines and regulations (S1 Checklist). This study was approved by the institutional review boards (IRBs) of Seoul National University Hospital (No. H-2201-147-1294), Dongguk University Ilsan Hospital (No. DUIH 2022-02-013-017), Chung-Ang University Hospital (No. 2204-006-502), Bundang CHA Hospital (CHAMC 2022-04-025-005), Seoul National University Bundang Hospital (B-2206-765-402), and Kyung Hee University Hospital (KHUH 2022-06-064-013). For more information, please see S1 Data.

### Informed consent

Informed consent will be obtained from every patient before any procedure related to this clinical trial is performed; IRB-approved forms will be used for informed consent and for providing an explanation to the patients. The principal investigator (or a delegated researcher) will explain the clinical trial to the patient. Patients who provide consent voluntarily to the study will be enrolled and provided with a copy of the signed informed consent form. The patient may withdraw consent at any time for any reason during the clinical trial.

### Patient and public involvement

The intervention program for this study was developed by a multidisciplinary expert group based on reviews of relevant literature and focus group interviews with five cancer patients, four caregivers of cancer patients, and healthcare professionals to explore their unmet medical needs.

## Discussion

Previous trial studies have shown that HbSC improved QoL [3, 4], reduced medical service use [5, 6], alleviated the side effects of anti-cancer drugs [7], and increased patient satisfaction [8] among patients with cancer. Additionally, HbSC was found to be associated with low rates of depression and admission to nursing institutions [9]. In contrast, other findings have indicated that a rapid transition to home-based palliative care has no significant effects on the survival rate and place of death [10, 11]. Based on these findings, although prior studies have found that HbSC improves QoL and reduces medical service use, there is no clear consensus on the benefits of HbSC [23–28, 32, 36, 39].

This STAHR study protocol and intervention program were developed based on a literature review and discussions. Many interventions in other studies provided supportive care at home based on a multidisciplinary team [3–5, 10, 12] and included education related to pain [13], programs to monitor side effects during the administration of anti-cancer drugs, visits to patients [7], programs to promote physical activity [14], and periodic visits and psychological support [12]. One-time visits and phone consultations did not have lasting effects [15], and long-term low-intensity visits by non-professional medical staff were also ineffective [12], suggesting that a multidisciplinary team is necessary to provide HbSC. In studies of severely ill patients, educating both the patients and their families was found to increase treatment compliance [11, 16]. These findings imply that caregivers who care for the patients must also be targeted by intervention programs [23–26, 29, 30, 32, 33, 35, 36, 40].

This study has some limitations. First, a non-randomized cluster-controlled trial can result in selection bias and the limited comparability between groups due to potential confounding factors. However, we believe that this study, which will investigate the effects of HbSC, is only feasible with this study design, as we previously described. Second, this study was performed in tertiary hospitals in South Korea, which may limit the generalizability of its findings to patients with advanced cancer in other countries. The differences in healthcare systems, healthcare insurance, religion, and cultural background may affect participant compliance with and applicability of the HbSC program. Third, it is generally recommended that at least four clusters per arm be used, but this study uses three clusters (hospitals) owing to feasibility issues [41]. To compensate, our statistical analysis methods will take into account how the smaller number of clusters could affect the results of a hierarchical mixed model [42, 43].

We believe that the STAHR trial for patients with advanced cancer will be beneficial, especially for those who require HbSC. Herein, we developed application protocols, clinical guidelines, and training programs for an evidence-based multidisciplinary home-based medical care intervention program for severely ill patients that suit the healthcare system in Korea. This study will also extend the implementation and dissemination of HbSC and produce a manual of HbSC intervention. In addition, if the effectiveness of the service can be confirmed, it could serve as evidence for HbSC to be covered by national health insurance. By conducting the STAHR study and reporting its results, our findings may provide the clinical basis and evidence for the utilization and application of the developed multidisciplinary home-based medical care intervention program.

### Trial status

The present protocol is version 1.2, dated March 4, 2022. Study participants have been recruited since August 1, 2022, and the trial has started.

## Supporting information

S1 Checklist SPIRIT checklist. (DOC)

S1 Data IRB approval documents. (ZIP)

S1 File (PDF)

S2 File (PDF)
