# Supplementary material for: SupporTive Care At Home Research (STAHR) for patients with advanced cancer: Protocol for a cluster non-randomized controlled trial
Source: PLoS One. 2024 May 13;19(5):e0302011. doi: 10.1371/journal.pone.0302011 (PMC11090303; doi:10.1371/journal.pone.0302011)
Supplement: S1 Data — (ZIP) [file pone.0302011.s002.zip › IRB_CAUH_2204-006-502_approval.pdf]

CAU

Chung-Ang University Hospital Institutional Review Board (IRB)

Tel :82-2-6299-2738 ~ 2741, FAX:82-2-6299-2739

102 Heukseok-ro Dongjak-Gu, Seoul 06973, Korea.

Official Report of IRB Review

This letter is to inform you of the results of IRB review.

**Reason for Request of review**	■ Initial Review ☐ Modifications or changes to the Protocol ☐ Unanticipated Problems or Non-Compliance ☐ Continuing Review ☐ Final Report ☐ Others		
**Title of Study**	A Cluster, Non-randomized Controlled Trial of the Effectiveness of a Korean Model for Home-based Care in Patients with Advanced Cancer		
	**Protocol No.**		**Version No.** 1.3 cau ver 1.0
**IRB No**	2204-006-502	**Date of Review**	23. May. 2022
**Investigator**	In-Gyu Hwang M.D., PhD.	**Department**	Department of hemato-oncology
**Study Agent(s)**	**Generic Names(s)**		**Brand Name(s)**
**Phase**	☐ Phase I ☐ Phase II ☐ Phase III ☐ Phase IV ☐ PMS ☐ Bioequivalence ■ others		
**Proposed Period of Study**	23. May. 2022 ~ 22. May. 2023		
**Sponsor**			
**Result of Review**	■ Approved ☐ Approved with Condition ☐ Modifications Requires ☐ Disapproved		
**Continuing review report interval**	**1 year**		
**Reviewer's comments**	The initial report was approved.		

This is to certify that the information contained herein is true and correct as reflected in the records of the Chung-Ang University Hospital IRB. We certify that Chung-Ang University Hospital IRB is in full compliance with Good Clinical Practice as defined under the Korea Food and Drug Administration (KFDA) regulations and functions in accordance with the ICH GCP Guidelines (CPMP/ICH/135/95) and the Korean national ethics requirements.

Jeong-Kyu Lee
Chairperson

23-May-22
Date
