# Supplementary material for: SupporTive Care At Home Research (STAHR) for patients with advanced cancer: Protocol for a cluster non-randomized controlled trial
Source: PLoS One. 2024 May 13;19(5):e0302011. doi: 10.1371/journal.pone.0302011 (PMC11090303; doi:10.1371/journal.pone.0302011)
Supplement: S1 Data — (ZIP) [file pone.0302011.s002.zip › IRB_CHAMC_2202-04-025-005_approval.pdf]

Institutional Review Board

CHA Bundang Medical Center, CHA University

59 Yatap-ro, Bundang-gu, Seongnam-si, Gyeonggi-do 463-712, KOREA

Tel: 82 31 780 5302 / Fax: 82 31 780 5305

Approval Date: 23 May, 2022

CERTIFICATE OF APPROVAL

The Institutional Review Board has approved the following documents related to the following protocol:

IRB No.	Title		Name of PI
**2022-04-025**	A Cluster, Non-randomized Controlled Trial of the Effectiveness of a Korean Model for Home-based Care in Patients with Advanced Cancer		Beodeul Kang
**Documents approved**	- Re-submitted Protocol		
**Frequency of continuing review**	Every 12 months	**Expiry of IRB Approval**	22 May, 2023

Post Approval Responsibilities:

1) This IRB complies with the KGCP and the ICH-GCP as well as applicable laws and regulations including the Bioethics and Safety Act.
2) The clinical research protocol that has been conditionally approved or requires modifications for approval can be reviewed again by the review committee by submitting supplementary data or objection data in accordance with Chapter 5.9 of the IRB SOP.
3) Conditionally approved protocols are subject to an expedited review, and those that require modifications for approval or have been rejected are subject to a full board review.
4) To the extent possible, the principal investigator must submit the complementary or objection data within one (1) month. If such data are not submitted within six (6) months after the notice of review, the IRB can suspend the review or progress of the study.
5) All amendments to the approved documents must be submitted to the IRB for approval before implementation. Before the relevant protocol is approved, the implementation of a clinical study different from the protocol is prohibited.
6) Subjects are not allowed to participate in clinical research before the relevant protocol is approved.
7) All SAEs occurred at this institution must be reported to the IRB within the reporting period specified by the IRB SOP.
8) The validity period for the study is up to one (1) year after the IRB approval date. The principal investigator must submit an interim report within 60 days prior to the end of the approval validity period and obtain the approval of such an interim report during the approval validity period. If an interim report is not submitted, the IRB may suspend the study.

9) To the extent possible, the completion report must be submitted within one (1) month after the completion of the study, and, to the extent possible, the final result report must be submitted within six (6) months after the approval of the completion report. However, the deadline may be extended for multi-country or multi-center studies.
10) Study-related documents must be handed over to the document storage manager within six (6) months after the final result report. (Only applicable to the clinical trials)
11) The version finally approved by the IRB must be used for the subjects' informed consent form with perforation and the IRB's approval stamp.
12) If necessary, the IRB may inspect the study.
13) If the principal investigator or the researcher in charge (including those related to the study) is an IRB member, he or she has no right to vote or speak and may not participate in the relevant review of the clinical study.
14) According to the Declaration of Helsinki, all clinical trials should be registered and disclosed in the publicly accessible Primary Registry prior to the recruitment of the first subject. For example, the Clinical Research Information Service (http://cris.nih.go.kr) may be used.

Institutional Review Board.
