# Supplementary material for: SupporTive Care At Home Research (STAHR) for patients with advanced cancer: Protocol for a cluster non-randomized controlled trial
Source: PLoS One. 2024 May 13;19(5):e0302011. doi: 10.1371/journal.pone.0302011 (PMC11090303; doi:10.1371/journal.pone.0302011)
Supplement: S1 Data — (ZIP) [file pone.0302011.s002.zip › IRB_DUIH 2022-02-013-017_approval.pdf]

Certificate of Approval

DUIH IRB
Dongguk University Ilsan Hospital
Institutional Review Board

Tel :82-31-961-8405
FAX:82-31-961-8449
27 Dongguk-ro, Ilsandong-gu, Goyang-si, Gyeonggi-do, 10326, Korea

THE FOLLOWING WERE APPORVED:

BOARD ACTION DATED: **26 April 2022**
STUDY NO: **N/A**
IRB NO: **DUIH 2022-02-013**

INVESTIGATOR: **Dr. Do Yeun Kim, Professor, Department of Hematology and Medical Oncology, Dongguk University Ilsan Hospital**

SPONSOR: **N/A (Investigator Initiated Trial)**

PROTOCOL NO: **N/A**

TITLE: **A Cluster, Non-randomized Controlled Trial of the Effectiveness of a Korean Model for Home-based Care in Patients with Advanced Cancer**

APPROVAL INCLUDES:

ALL CONDITIONS OF APPROVAL PREVIOUSLY ESTABLISHED BY DUIH IRB
FOR THIS RESEARCH PROJECT CONTINUE TO APPLY.

CONTINUING REVIEW REPORT INTERVAL: **1 year**

DISTRIBUTION OF COPIES

SPONSOR: **N/A**

CRO: **N/A**

OTHER INSTITUTION: **N/A**

IF YOU HAVE ANY QUESTIONS, CONTACT DUIH IRB(Tel: 82-31-961-8405)

This is to certify that the information contained herein is true and correct as reflected in the records of the DUIH Institutional Review Board. **We certify that DUIH IRB is in full compliance with Good Clinical Practice as defined under the Ministry of Food and Drug Safety (MFDS) regulations and the International Conference on Harmonisation (ICH) guidelines.**

[signature]	05 October 2023
**Chairperson**	**Date**

ALL DUIH IRB APPROVED INVESTIGATORS MUST COMPLY WITH THE FOLLOWING:

1. Conduct the research as required by the protocol.
2. Provide non-Korean speaking subjects with a certified translation of the approved Consent Form in the subject's first language. The translated version must be approved by the DUIH IRB.
3. Obtain pre-approval from the DUIH IRB of any changes in the research activity (except when necessary to protect human subjects; immediately report to the DUIH IRB any such emergency changes for the protection of human subjects).
4. Report to the DUIH IRB the death, hospitalization, or serious illness of any study subject.
5. Promptly report to the DUIH IRB any new information that may adversely affect the safety of the subjects or the conduct of the trial.
6. Provide reports to the DUIH IRB concerning the progress of the research, when requested.
7. Obtain pre-approval of study advertisements from the DUIH IRB before use.
8. Conduct the informed consent process without coercion or undue influence, and provide the potential subject sufficient opportunity to consider whether or not to participate.

Ministry of Food and Drug Safety regulations require that the DUIH IRB conduct review of approved research. You will receive Continuing Review Report forms from the DUIH IRB. These reports must be returned even though your study may not have started.

DISTRIBUTION OF COPIES

SPONSOR: N/A

CRO: N/A

OTHER INSTITUTION: N/A

This is to certify that the information contained herein is true and correct as reflected in the records of the DUIH Institutional Review Board. **We certify that DUIH IRB is in full compliance with Good Clinical Practice as defined under the Ministry of Food and Drug Safety (MFDS) regulations and the International Conference on Harmonisation (ICH) guidelines.**
