# Supplementary material for: SupporTive Care At Home Research (STAHR) for patients with advanced cancer: Protocol for a cluster non-randomized controlled trial
Source: PLoS One. 2024 May 13;19(5):e0302011. doi: 10.1371/journal.pone.0302011 (PMC11090303; doi:10.1371/journal.pone.0302011)
Supplement: S1 Data — (ZIP) [file pone.0302011.s002.zip › IRB_DUIH 2022-02-013-017_approval_extension_document_kor.pdf]

통지서

※ 본 과제의 문서보존기간은 3 년입니다.						
수신	의뢰(지원)기관	한국보건의료연구원				
	연구책임자	내과 김도연				
IRB File No.		DUIH 2022-02-013-020	심사내용	중간보고서	통지일자	2023.03.02
연구과제명	국문	항암치료를 지속 중인 고형암 환자를 대상으로 재택의료를 제공하여 등록부터 6개월 이내 예정되지 않은 입원 감소 효과를 평가하기 위한 군집 비무작위 배정 연구자 주도 임상시험				
	영문	A Cluster, Non-randomized Controlled Trial of the Effectiveness of a Korean Model for Home-based Care in Patients with Advanced Cancer				
임상시험코드				Study Nick Name		

연구분류1	☐ 약물 ☐ 생물학적 제재 ☐ 세포치료제 ☐ 건강기능식품
	☐ 의료시술 ☐ 의료기기 (○ 1등급 ○ 2등급 ○ 3등급 ○ 4등급)
	■ 해당사항없음
연구분류2	■ 인간대상연구 ☐ 인체유래물(검체)연구 ☐ 의무기록연구
	☐ 유전자연구 ☐ 유전자치료
	☐ 배아연구 ☐ 체세포복제배아연구 ☐ 줄기세포주연구
	☐ 기타 ( )
연구분류3	◉ 전향적 연구 ○ 후향적 연구 ○ 전항적 & 후향적 병행연구
연구분류 4	■ 중재연구 ☐ 설문조사 ☐ 자료분석 및 분석연구
	☐ 관찰연구 (☐ 단면조사연구 ☐ 환자대조군연구 ☐ 코호트 연구)
	☐ 기타 ( )
연구분류 5	☐ 인간을 대상으로 하지 않는 연구 Non-clinical study (in vitro. in vivo preclinical study)

일반명			상품명			
전체피험자증례수	전체	396 명	국내	명	본원	66 명

본 서식은 전자서식(PDF 파일)으로 발급되었습니다.

바코드가 입력되지 않은 전자서식은 확인용 전용뷰어로 진본 여부를 확인할 수 없으며, 진본 여부가 표시되지 않습니다.

<table>
<tr><td>연구승인기간</td><td colspan="7">2022.04.26 ~ 2024.04.25</td></tr>
<tr><td>지원의뢰기관</td><td>기관명</td><td colspan="2">한국보건의료연구원</td><td>대표(직위)</td><td></td><td>성명</td><td>한광협</td></tr>
<tr><td>제출서류목록</td><td colspan="7">(첨부) 연구계획서 [1.6] [221202]
(첨부) 연구대상자 설명문 및 동의서 [1.5] [221027]
(첨부) 연구과제점검리스트 [] []</td></tr>
<tr><td>관련근거</td><td>평가일자</td><td colspan="6">2023.03.02</td></tr>
<tr><td>중간보고시기</td><td colspan="2">2024년 02월 25일까지</td><td>비고</td><td colspan="4"></td></tr>
<tr><td>심사결과</td><td colspan="7">◉ 승인 ○ 시정승인</td></tr>
<tr><td>심사결과</td><td colspan="7">상기 과제의 과제점검리스트 및 제출된 서류를 검토한 결과, 연구의 최초 승인 이후 위험 대비 이득을 비추어볼 때 추가적으로 연구대상자에 대한 위험이 유의하게 증가하지 않은 것으로 판단되어 ‘승인’합니다.

■ 중간보고 승인일: 2023.03.02

[안내사항]
1. 연구자는 지속심의 주기에 맞춰 지속심의를 제출 및 승인 받아야 하며, 연구가 종료된 경우 6개월 이내에 종료보고를 하여야 합니다.</td></tr>
</table>

본 위원회에서 승인된 모든 연구자들은 다음의 사항을 준수하여야 합니다.

1. 연구계획서 및 변경계획서의 승인 이전에 연구대상자의 해당 임상연구의 참여 금지됩니다.
2. 승인 받은 계획서에 따라 연구를 수행하여야 합니다. 변경계획서에 대한 승인 이전에 원 임상연구 계획서와 다른 임상연구의 실시는 금지됩니다.
3. IRB 승인 받은 동의서를 사용하여야 합니다.
4. 연구대상자에게 강제 혹은 부당한 영향이 없는 상태에서 충분한 설명에 근거하여 동의과정을 수행할 것이며, 잠재적인 연구대상자에게 연구의 참여여부를 고려할 수 있도록 충분한 기회를 제공하여야 합니다.
5. 연구진행에 있어 연구대상자를 보호하기 위해 불가피한 경우를 제외하고 연구의 어떠한 변경이든 위원회의 사전 승인을 받고 수행하여야 합니다. 연구대상자들의 보호를 위해 취해진 어떠한 응급상황에서의 변경도 즉각 위원회에 보고하여야 합니다.
6. 연구대상자에게 발생한 즉각적 위험 요소의 제거가 필요하여 원 계획서와 다르게 연구를 실시해야 하는 경우, 연구대상자에게 발생하는 위험요소를 증가 시키거나 연구의 실시에 중대한 영향을 미칠 수 있는 변경사항, 예상하지 못한 중대한 이상약물 / 의료기기 반응에 관한 사항, 연구대상자의 안전성이나 임상연구의 실시에 부정적인 영향을 미칠 수 있는 새로운 정보에 관한 사항은 위원회에 신속히 보고하여야 합니다.
7. 위원회의 승인을 받은 연구대상자 모집 광고문을 사용해야 합니다.
8. 위원회의 승인은 1년을 초과할 수 없습니다. 1년 이상 연구를 지속하고자 하는 경우에는 반드시 지속보고를 하여

본 서식은 전자서식(PDF 파일)으로 발급되었습니다.

바코드가 입력되지 않은 전자서식은 확인용 전용뷰어로 진본 여부를 확인할 수 없으며, 진본 여부가 표시되지 않습니다.

야 합니다.
9. 심의결과가 승인이 아닌 경우에는 답변서를 제출하여야 하며, 심의일로부터 4개월 이내에 이루어져야 합니다.
10. 연구 종료 시에는 종료 및 결과보고서를 작성하여 제출해야 합니다.
11. 생명윤리 및 안전에 관한 법률, 약사법 / 의료기기법, 헬싱키 선언 및 ICH-GCP 가이드라인 등 국내외 관련 법규를 준수하여야 합니다.
12. 승인 받은 연구에 대하여 기관의 내부 점검 및 외부의 실태조사를 받을 수 있습니다. 기관의 내부 점검자, 외부의 모니터요원 및 점검자, 규제기관의 실태조사자 등이 연구 관련 문서(전자문서 포함)에 대한 열람을 요청하는 경우 연구담당자는 이에 적극 협조해야 합니다.

▶ 본 연구와 이해상충(COI, Conflict of Interest)이 있는 위원은 심사 결정과정에 참여하지 않습니다.
▶ 문서 하단의 바코드를 스캐너로 확인하여 위변조 여부를 확인할 수 있습니다.
▶ 본 위원회는 국제 임상시험 통일안(ICH) 및 임상시험관리기준(GCP)을 준수합니다.

동국대학교일산병원 기관윤리심의위원회

동국대학교일산불교병원기관윤리심의위원회위원장인

본 서식은 전자서식(PDF 파일)으로 발급되었습니다.
바코드가 입력되지 않은 전자서식은 확인용 전용뷰어로 진본 여부를 확인할 수 없으며, 진본 여부가 표시되지 않습니다.
