# Supplementary material for: SupporTive Care At Home Research (STAHR) for patients with advanced cancer: Protocol for a cluster non-randomized controlled trial
Source: PLoS One. 2024 May 13;19(5):e0302011. doi: 10.1371/journal.pone.0302011 (PMC11090303; doi:10.1371/journal.pone.0302011)
Supplement: S1 Data — (ZIP) [file pone.0302011.s002.zip › IRB_KHUH-2022-06-064-013_approval_extension_document_kor.pdf]

통지서

※ 본 과제의 문서보존기간은 3 년입니다.						
수신	의뢰(지원)기관	서울대학교병원				
	연구책임자	종양혈액내과 백선경				
IRB File No.		KHUH 2022-06-064-025	심사내용	중간보고서	통지일자	2023.08.28
연구과제명	국문	항암치료를 지속 중인 고형암 환자를 대상으로 재택의료를 제공하여 등록부터 6개월 이내 예정되지 않은 입원 감소 효과를 평가하기 위한 군집 비무작위 배정 연구자 주도 임상시험				
	영문	A Cluster, Non-randomized Controlled Trial of the Effectiveness of a Korean Model for Home-based Care in Patients with Advanced Cancer				
임상시험코드			Study Nick Name			

연구분류1	□ 약물 □ 생물학적 제재 □ 세포치료제 □ 건강기능식품
	□ 의료시술 □ 의료기기 (○ 1등급 ○ 2등급 ○ 3등급 ○ 4등급)
	■ 해당사항없음
연구분류2	■ 인간대상연구 □ 인체유래물(검체)연구 □ 의무기록연구
	□ 유전자연구 □ 유전자치료
	□ 배아연구 □ 체세포복제배아연구 □ 줄기세포주연구
	□ 기타 ( )
연구분류3	◉ 전향적 연구 ○ 후향적 연구 ○ 전향적 & 후향적 병행연구
연구분류 4	□ 중재연구 ■ 설문조사 □ 자료분석 및 분석연구
	■ 관찰연구 (□ 단면조사연구 □ 환자대조군연구 ■ 코호트 연구)
	□ 기타 ( )
연구분류 5	□ 인간을 대상으로 하지 않는 연구 Non-clinical study (in vitro. in vivo preclinical study)

일반명			상품명		
전체피험자증례수	전체	396 명	국내	396 명	본원	66 명

본 서식은 전자서식(PDF 파일)으로 발급되었습니다.

바코드가 입력되지 않은 전자서식은 확인용 전용뷰어로 진본 여부를 확인할 수 없으며, 진본 여부가 표시되지 않습니다.

<table>
<tr><td>연구승인기간</td><td colspan="6">2023.08.21 ~ 2024.08.20</td></tr>
<tr><td>지원의뢰기관</td><td>기관명</td><td>서울대학교병원</td><td>대표(직위)</td><td></td><td>성명</td><td>김영태</td></tr>
<tr><td>제출서류목록</td><td colspan="6">- 연차지속심의신청서
- 연구계획서 [1.9]
- 연구계획서 변경대비표[1.3] -> [1.4] -> [1.5] -> [1.6] -> [1.9]
- 증례기록서(CRF) [1.7]
- 연구대상자설명서 및 동의서(ICF) [1.4]
- 연구대상자설명서 및 동의서 변경대비표(ICF) [1.1] -> [1.4] -> [1.2] -> [1.3] -> [1.4]
- 연구정보의 제 3자 제공 및 2차 연구 이용에 대한 동의서 [1.5]
- 연구정보의 제 3자 제공 및 2차 연구 이용에 대한 동의서 변경대비표 [1.1] -> [1.4] -> [1.5]
- 환자용 설문지 [1.4]
- 보호자용 설문지 [1.4]
- 연구비 산정 내역서(140,600,000원)
- 시험책임자 이력서 : 종양혈액내과 백선경 교수
- 자료 및 안전성 모니터링 계획서[1.2]
- 위임서명록
- 주관기관승인서
- 시정승인 답변자료
- 대상자 진행현황리스트
- 공동연구자 변경대비표</td></tr>
<tr><td>관련근거</td><td>대면회의</td><td colspan="5">2023.08.21</td></tr>
<tr><td>중간보고시기</td><td colspan="2">2024년 08월 20일까지</td><td>비고</td><td colspan="3"></td></tr>
<tr><td>심사결과</td><td colspan="6">◉ 승인 ○ 시정승인 ○ 보완 ○ 반려</td></tr>
<tr><td>심사결과</td><td colspan="6">본 위원회는 상기 연구의 제출된 서류를 검토한 결과, 최초 승인 이후 추가적으로 대상자에 대한 위험이 증가하지 않을 것으로 판단한 바, 상기 연구심의신청서에 대해 승인하기로 결정하였습니다.

※ 중간심의 주기 : 연차심의로 대체(12개월)

※ 보완을 요하는「보완」,「시정승인」시에는 (통지일로부터 3개월 내에) 보완자료 또는 이의에 관한 자료 등을 임상시험심사위원회에 제출하여 주십시오. 기간 내에 보완자료 또는 이의에 관한 자료를 제출하지 않으면 부결 처리됩니다. 문의사항은 행정간사 : 한수진(전화 : 958-9579, 팩스:958-9559, 이메일:khcri@khmc.or.kr), 행정직원 : 신지은(전화: 958-9571, 팩스: 958-9559, 이메일: 27459@khmc.or.kr)로 연락하시면 됩니다.</td></tr>
</table>

본 서식은 전자서식(PDF 파일)으로 발급되었습니다.

바코드가 입력되지 않은 전자서식은 확인용 전용뷰어로 진본 여부를 확인할 수 없으며, 진본 여부가 표시되지 않습니다.

※ 경희대학교병원 임상시험심사위원회는 국제임상시험 통일안 ICH / GCP 및 임상시험관리기준 (GCP) , 생명윤리 및 안전에 관한 법률 등 관련 법규를 준수합니다.
※ 이 연구와 이해관계 (Conflict of Interest) 가 있는 위원이 있을 경우 이 연구의 심의에서 배제하였습니다.
※ 본 임상연구 결과는 임상시험실시기관의 사전 서면동의 없이는 어떤 경우라도 학술목적 이외에 실시기관명을 사용할 수 없습니다.
※ 문서 하단의 바코드를 스캐너로 확인하여 위변조 여부를 확인할 수 있습니다.

경 희 대 학 교 병 원 임 상 시 험 심 사 위 원 회 위 원 장

본 서식은 전자서식(PDF 파일)으로 발급되었습니다.
바코드가 입력되지 않은 전자서식은 확인용 전용뷰어로 진본 여부를 확인할 수 없으며, 진본 여부가 표시되지 않습니다.
