# Supplementary material for: SupporTive Care At Home Research (STAHR) for patients with advanced cancer: Protocol for a cluster non-randomized controlled trial
Source: PLoS One. 2024 May 13;19(5):e0302011. doi: 10.1371/journal.pone.0302011 (PMC11090303; doi:10.1371/journal.pone.0302011)
Supplement: S1 Data — (ZIP) [file pone.0302011.s002.zip › IRB_KHUH-2022-06-064-013_approval_kor.pdf]

통지서

※ 본 과제의 문서보존기간은 3 년입니다.						
수신	의뢰(지원)기관	서울대학교병원				
	연구책임자	종양혈액내과 백선경				
IRB File No.		KHUH 2022-06-064-003	심사내용	시정계획서	통지일자	2022.07.25
연구과제명	국문	항암치료를 지속 중인 고형암 환자를 대상으로 재택의료를 제공하여 등록부터 6개월 이내 예정되지 않은 입원 감소 효과를 평가하기 위한 군집 비무작위 배정 연구자 주도 임상시험				
	영문	A Cluster, Non-randomized Controlled Trial of the Effectiveness of a Korean Model for Home-based Care in Patients with Advanced Cancer				
임상시험코드			Study Nick Name			

연구분류1	☐ 약물	☐ 생물학적 제재	☐ 세포치료제	☐ 건강기능식품	
	☐ 의료시술	☐ 의료기기	(○ 1등급	○ 2등급	○ 3등급 ○ 4등급)
	■ 해당사항없음				
연구분류2	■ 인간대상연구	☐ 인체유래물(검체)연구	☐ 의무기록연구		
	☐ 유전자연구	☐ 유전자치료			
	☐ 배아연구	☐ 체세포복제배아연구	☐ 줄기세포주연구		
	☐ 기타 ( )				
연구분류3	◉ 전향적 연구	○ 후향적 연구	○ 전향적 & 후향적 병행연구		
연구분류 4	☐ 중재연구	■ 설문조사	☐ 자료분석 및 분석연구		
	■ 관찰연구 (	☐ 단면조사연구	☐ 환자대조군연구	■ 코호트 연구)	
	☐ 기타 ( )				
연구분류 5	☐ 인간을 대상으로 하지 않는 연구 Non-clinical study (in vitro. in vivo preclinical study)				
일반명		상품명			
전체피험자증례수	전체 396 명	국내 396 명	본원 66 명		

본 서식은 전자서식(PDF 파일)으로 발급되었습니다.

바코드가 입력되지 않은 전자서식은 확인용 전용뷰어로 진본 여부를 확인할 수 없으며, 진본 여부가 표시되지 않습니다.

연구승인기간	2022.07.22 ~ 2023.07.21					
지원의뢰기관	기관명	서울대학교병원	대표(직위)		성명	김연수
제출서류목록	(첨부) 연구계획서 [1.3]<br>(첨부) 시험(연구)대상자설명서 및 동의서(ICF) [1.1]<br>(첨부) 연구정보의 제 3자 제공 및 2차 연구 이용에 대한 동의서[1.1]<br>(첨부) 심사의견에 대한 답변서<br>(첨부) Protocol agreement<br>(첨부) 변경대비표					
관련근거	평가일자	2022.07.22				
중간보고시기	2023년 07월 21일까지		비고			
심사결과	◉ 승인 ○ 시정승인					
심사결과	〈위원회 심사의견에 대한 답변서〉<br>1) 198명에서 15% 탈락을 감안할 경우 172명이 달성되지 않습니다. 표본 수의 계산을 다시 확인하여 주십시오.<br>[답변]기존에 제출한 protocol이 서울대병원에서 승인 전의 버전으로 표본의 수 관련 산출식등을 포함하고 있는 변경된 1.3ver으로 제출합니다.<br><br>2) 연구계획서 내 1차 평가 변수가 상이하오니 일치시켜 주십시오(6페이지 : 연구 등록 후 6개월간 계획되지 않은 입원의 수, 22페이지 : 연구 등록 후 6개월간 계획되지 않은 입원을 한 환자의 분율).<br>[답변]6페이지: 연구 등록 후 6개월간 계획되지 않은 입원의 수 / 23페이지: 연구 등록 후 6개월간 계획되지 않은 입원을 한 환자의 수로 수정하였습니다.<br><br>3) 파일 첨부된 수정된 연구대상자설명서 및 동의서(연구정보의 제3자 제공 및 2차 연구 이용에 대한 동의서 포함)를 참고하여 수정해 주십시오.<br>[답변]파일에 첨부된 양식으로 연구대상자설명서 및 동의서(연구정보의 제3자 제공 및 2차 연구 이용에 대한 동의서 포함)를 수정하여 첨부합니다<br><br>※ 워터마크가 삽입된 동의서로 출력하여 대상자에게 동의를 취득하여 주십시오.<br><br>※ 연구계획서는 서울대학교병원 기준 Ver 1.0 -〉 Ver 1.3으로 변경함<br><br>※ 문의사항은 행정간사: 한경화, 전화: 958-9566, 팩스:958-9559, 이메일: yynam@khmc.or.kr로 연락하시면 됩니다.<br><br>※ 신속심사점검표에서 대상자의 Risk를 증가시키지 않거나 최소위험 이하인 각종 보고나 변경사항에 해당되어 신속심사로 진행함.					

본 서식은 전자서식(PDF 파일)으로 발급되었습니다.
바코드가 입력되지 않은 전자서식은 확인용 전용뷰어로 진본 여부를 확인할 수 없으며, 진본 여부가 표시되지 않습니다.

심사결과	-IRB의 심사 결과(시정승인)에 따라 보완되어 제출된 계획서의 심사<br>(Review of amended protocol as instructed by the IRB review)

※ 경희대학교병원 임상시험심사위원회는 국제임상시험 통일안 ICH / GCP 및 임상시험관리기준（GCP）, 생명윤리 및 안전에 관한 법률 등 관련 법규를 준수합니다.
※ 이 연구와 이해관계（Conflict of Interest）가 있는 위원이 있을 경우 이 연구의 심의에서 배제하였습니다.
※ 본 임상연구 결과는 임상시험실시기관의 사전 서면동의 없이는 어떤 경우라도 학술목적 이외에 실시기관명을 사용할 수 없습니다.
※ 문서 하단의 바코드를 스캐너로 확인하여 위변조 여부를 확인할 수 있습니다.

경 희 대 학 교 병 원 임 상 시 험 심 사 위 원 회 위 원 장

본 서식은 전자서식(PDF 파일)으로 발급되었습니다.

바코드가 입력되지 않은 전자서식은 확인용 전용뷰어로 진본 여부를 확인할 수 없으며, 진본 여부가 표시되지 않습니다.
