# Supplementary material for: SupporTive Care At Home Research (STAHR) for patients with advanced cancer: Protocol for a cluster non-randomized controlled trial
Source: PLoS One. 2024 May 13;19(5):e0302011. doi: 10.1371/journal.pone.0302011 (PMC11090303; doi:10.1371/journal.pone.0302011)
Supplement: S1 Data — (ZIP) [file pone.0302011.s002.zip › IRB_SNUBH_B-2206-765-402_approval.pdf]

SEOUL NATIONAL UNIVERSITY BUNDANG HOSPITAL INSTITUTIONAL REVIEW BOARD

TEL: 82-31-787-8801~6
FAX:82-31-787-8869
ADDRESS:82, GUMI-RO 173BEON-GIL BUNDANG-GU, SEONGNAM-SI, GYEONGGI- DO, 13620, KOREA

SNUH 분당서울대학교병원

IRB Decision Notification Document

The followings were Reviewed:

IRB No: B-2204-750-401

Research Title: Baseline study for Development and Evaluation of the Effectiveness of Korean Model for Home-based Care for Patients with Serious Illness

Protocol No: **/ Version No.:** 1.0

Investigator: Yu Jung Kim

Sponsor: SCRIPT ERROR

Protocol Approval Date: 2022-03-24 **/ Approval effective period:** 2023-03-23
Continuing Review Report Interval: 12 Months

Receipt Date: 2022-03-24

Review Date: 2022-03-24

Risk of the Study: Minimal Risk

Review Type: Application for Protocol Review

Review item: Joint Review

Review List & Comments :

Review Result: APPROVAL

All conditions of approval previously established by SNUBH IRB for this research project continue to apply.
If you have any questions, contact SNUBH IRB at 82-31-787-8801~6

분당서울대학교병원생명윤리심의위원회위원장인

2022-03-24

Chairperson: **Date**

This is to certify that the information contained herein is true and correct as reflected in the records of the SNUBH Institutional Review Board (SNUBH IRB).

WE CERTIFY THAT SNUBH IRB IS IN FULL COMPLIANCE WITH GOOD CLINICAL PRACTICES AS DEFINED UNDER THE KOREA FOOD AND DRUG ADMINISTRATION (MFDS) REGULATIONS AND THE INTERNATIONAL CONFERENCE ON HARMONISATION (ICH) GUIDELINES.

SEOUL NATIONAL UNIVERSITY BUNDANG HOSPITAL
INSTITUTIONAL REVIEW BOARD

TEL: 82-31-787-8801~6
FAX:82-31-787-8869

ADDRESS:82, GUMI-RO 173BEON-GIL BUNDANG-GU, SEONGNAM-SI, GYEONGGI- DO, 13620, KOREA

IRB Decision Notification Document

All investigators performing SNUBH IRB approved projects must comply with the followings:

1. Enrollment of participated subjects before the IRB approval of protocol/protocol amendment is forbidden.

2. To conduct the study according to the approved protocol. To conduct the study differently from the original protocol is forbidden.

3. To use the approved Informed Consent Form.

4. The informed consent process shall be conducted based on sufficient explanation under no coercion or unfair influence, and a potential subject shall be provided with sufficient opportunity to consider the study participation.

5. Except for the unavoidable cases to protect subjects during the study conduct, any amendment of the study shall be implemented after getting the prior approval of the Board, and any amendment taken in an emergency situation for protection of subjects shall be immediately reported to the Board.

6. In case the study should be conducted differently from the original protocol since the immediate risk factor occurring to subjects should be eliminated, the amendment item that may increase risk factors occurring to subjects or have serious effects of the study conduct, items on the unexpected serious adverse drug reaction, or items on new information that may have negative effects on subjects' safety or study conduct shall be promptly reported to the Board.

7. The subject recruitment advertisement approved by the Board shall be used.

8. The Board approval period may not exceed one year. In case of intending to continue the study for more than one year, you are required to submit an annual continuation report, submit a report on the study progress in line with the interim reporting cycle as requested by the Board.

9. In case the IRB review decision is not an 'Approval', written response for IRB decision result shall be submitted within six months since the IRB review date.

10. In the case of a decision by the Board to disapprove, you may have the opportunity to submit an appeal in writing. However, you should not file an appeal 2 times in a row with the same reason.

11. When completing the research, Study completion report and Study result report shall be submitted.

12. You shall comply with Bioethics and Safety Act, Pharmaceutical Affairs Act/Medical Device Affairs Act as defined under the Ministry of Food and Drug Safety (MFDS) regulations, the International Conference on Harmonization (ICH) guidelines and the Declaration of Helsinki.

13. According to the Declaration of Helsinki, all clinical studies shall be disclosed in the database that allows public access(primary registry) prior to the first subject enrollment; for example, you may use http://register.clinicaltrials.gov. For details, please refer to the IRB website.

14. The internal audit or inspection from the regulatory agency for the approved study could be conducted. Investigator shall cooperate in helping this to carry out, when requested for reading of study document(including electronic document) by internal auditor or monitor from sponsor, or inspector from regulatory agency.

Korea FDA regulations require that the SNUBH IRB conduct review of approved research. You will receive Continuing Review Report forms from the SNUBH IRB. These reports must be returned even though your study may not have started.

This is to certify that the information contained herein is true and correct as reflected in the records of the SNUBH Institutional Review Board (SNUBH IRB).

WE CERTIFY THAT SNUBH IRB IS IN FULL COMPLIANCE WITH GOOD CLINICAL PRACTICES AS DEFINED UNDER THE KOREA FOOD AND DRUG ADMINISTRATION (MFDS) REGULATIONS AND THE INTERNATIONAL CONFERENCE ON HARMONISATION (ICH) GUIDELINES.
