# Supplementary material for: SupporTive Care At Home Research (STAHR) for patients with advanced cancer: Protocol for a cluster non-randomized controlled trial
Source: PLoS One. 2024 May 13;19(5):e0302011. doi: 10.1371/journal.pone.0302011 (PMC11090303; doi:10.1371/journal.pone.0302011)
Supplement: S1 Data — (ZIP) [file pone.0302011.s002.zip › IRB_SNUH_H-2201-147-1294_approval.pdf]

SNUCM/SNUH IRB
Seoul National University College of Medicine
Seoul National University Hospital Institutional Review Board

Tel :82-2-2072-0694
FAX:82-2-3675-6824 101 Daehak-ro, Jongno-gu, Seoul, 110-744, Korea

IRB Decision Notification Document

IRBNO : H-2201-147-1294

RESEARCH TITLE : A Cluster, Non-randomized Controlled Trial of the Effectiveness of a Korean Model for Home-based Care in Patients with Advanced Cancer

INVESTIGATOR : BELONG CHO

SPONSOR :

REVIEW LIST:
0. Application
1. Home Medical Care_Solid Cancer_Protocol_ver1.2
2. Home Medical Care_Solid Cancer_Third-Party Consent_v1.1
3. Home Medical Care_Solid Cancer_CRF_v1.0
4. Professor Cho Bi-ryong's CV V1.0
5. Home Medical Care_Solid Cancer_Information Sheet and Informed Consent_v1.2
6. Home Medical Care_Solid Cancer_Questionnaire for Guardians_v1.0
7. Home medical_solid cancer_questionnaire_v1.0
8. Home Medical Care_Solid Cancer_Recruitment Notice_v1.1
9. Home medical care_Statement of solid cancer_Statement of Research Grants_V1.0
10. Home Medical Care_Solid Cancer_DSMP_v1.0
11. [Appendix 4] In-home care educational materials
12. [Appendix 5] Home care education materials_Medical device supplement
13. Home Medical Care_Solid Cancer_Indemnification Provisions for Subjects v1.1

Review Comment:
According to [the IRB Approval Criteria], the IRB approves the research.

ReView Date : 2022-04-04

ALL CONDITIONS OF APPROVAL PREVIOUSLY ESTABLISHED BY SNUCM/SNUH IRB
FOR THIS RESEARCH PROJECT CONTINUE TO APPLY.

CONTINUING REVIEW REPORT INTERVAL: (12) Month

IF YOU HAVE ANY QUESTIONS, CONTACT SNUCM/SNUH IRB (Tel: 82-2-2072-0694)

Institutional Review Board Chairman

All investigators performing SNUBH IRB approved projects must comply with the followings:

1. Enrollment of participated subjects before the IRB approval of protocol/protocol amendment is forbidden.

2. To conduct the study according to the approved protocol. To conduct the study differently from the original protocol is forbidden.

3. To use the approved Informed Consent Form.

4. The informed consent process shall be conducted based on sufficient explanation under no coercion or unfair influence, and a potential subject shall be provided with sufficient opportunity to consider the study participation.

5. Except for the unavoidable cases to protect subjects during the study conduct, any amendment of the study shall be implemented after getting the prior approval of the Board, and any amendment taken in an emergency situation for protection of subjects shall be immediately reported to the Board.

6. In case the study should be conducted differently from the original protocol since the immediate risk factor occurring to subjects should be eliminated, the amendment item that may increase risk factors occurring to subjects or have serious effects of the study conduct, items on the unexpected serious adverse drug reaction, or items on new information that may have negative effects on subjects' safety or study conduct shall be promptly reported to the Board.

7. The subject recruitment advertisement approved by the Board shall be used.

8. The Board approval period may not exceed one year. In case of intending to continue the study for more than one year, you are required to submit an annual continuation report.

9. In case the IRB review decision is not an 'Approval', written response for IRB decision result shall be submitted within six months since the IRB review date.

10. In the case of a decision by the Board to disapprove, you may have the opportunity to submit an appeal in writing. However, you should not file an appeal 2 times in a row with the same reason.

11. When completing the research, Study completion report and Study result report shall be submitted.

12. You shall comply with Bioethics and Safety Act, Pharmaceutical Affairs Act/Medical Device Affairs Act as defined under the Ministry of Food and Drug Safety (MFDS) regulations, the International Conference on Harmonization (ICH) guidelines and the Declaration of Helsinki.

13. According to the Declaration of Helsinki, all clinical studies shall be disclosed in the database that allows public access(primary registry) prior to the first subject enrollment; for example, you may use http://register.clinicaltrials.gov. For details, please refer to the IRB website.

14. The internal audit or inspection from the regulatory agency for the approved study could be conducted. Investigator shall cooperate in helping this to carry out, when requested for reading of study document (including electronic document) by internal auditor or monitor from sponsor, or inspector from regulatory agency.

This is to certify that the information contained herein is true and correct as reflected in the records of the SNUCM/SNUH IRB. **We certify that SNUCM/SNUH IRB is in full compliance with Good Clinical Practice as defined under the Korean Ministry of Food and Drug Safety (MFDS) regulations and the International Conference on Harmonisation (ICH) guidelines.**
