# Supplementary material for: SupporTive Care At Home Research (STAHR) for patients with advanced cancer: Protocol for a cluster non-randomized controlled trial
Source: PLoS One. 2024 May 13;19(5):e0302011. doi: 10.1371/journal.pone.0302011 (PMC11090303; doi:10.1371/journal.pone.0302011)
Supplement: S1 File — (PDF) [file pone.0302011.s003.pdf]

임상시험 계획서

항암치료를 지속 중인 고형암 환자를 대상으로 재택의료를 제공하여 등록부터 6개월 이내 예정되지 않은 입원 감소 효과를 평가하기 위한 군집 비무작위 배정 연구자 주도 임상시험

A Cluster, Non-randomized Controlled Trial of the Effectiveness of a Korean Model for Home-based Care in Patients with Advanced Cancer

책임 연구자 소속: 서울대학교병원 공공진료센터
책임 연구자 이름: 조비룡

임상연구계획서 개요

국문 제목	항암치료를 지속 중인 고형암 환자를 대상으로 재택의료를 제공하여 등록부터 6개월 이내 예정되지 않은 입원 감소 효과를 평가하기 위한 군집 비무작위 배정 연구자 주도 임상시험
영문 제목	A Cluster, Non-randomized Controlled Trial of the Effectiveness of a Korean Model for Home-based Care in Patients with Advanced Cancer
시험조정자	서울대학교병원 가정의학과/공공진료센터 조비룡
연구 목적	거동에 어려움이 있는 진행암 환자와 가족 보호자에게 재가돌봄 교육을 제공하고 의료진의 가정방문, 주기적 상태 점검을 포함하는 재택의료 중재 프로그램을 제공하는 것이 6개월 이내 예정되지 않은 입원을 감소시키는데 미치는 효과를 검증하고자 함.
연구 설계	국내 다기관 군집 비 무작위 배정 임상시험
연구 기간	IRB승인일 ~ 2026년 12월 31일
연구 가설	1. 재가돌봄 교육 및 재택의료 중재 프로그램을 시행한 진행암 환자에서 대조군에 비해 예정되지 않은 입원, 응급실 방문 등 의료 이용이 유의하게 줄어들 것임.<br>2. 재가돌봄 교육 및 재택의료 중재 프로그램을 시행한 진행암 환자에서 대조군에 비해 삶의 질이 유의하게 높고, 증상 조절 정도가 유의하게 좋을 것임.<br>3. 재가돌봄 교육 및 재택의료 중재 프로그램을 시행한 진행암 환자에서 대조군에 비해 사전돌봄계획 수립 비율이 유의하게 높을 것임.<br>4. 재가돌봄 교육 및 재택의료 중재 프로그램을 시행한 진행암 환자에서 대조군에 비해 사망률이 유의하게 줄어들 것임.
연구근거	-중증질환자에게 재가환경 기반 중재를 시행하였을 때 기존의 입원과 외래 서비스만을 제공하는 것에 비해 재입원 또는 사망률을 개선한다는 것이 알려져 있음 (Malik, A. H., et al. (2019). "Effect of home-based follow-up intervention on readmissions and mortality in heart failure patients: a meta-analysis." Future Cardiol 15(5): 377-386.)<br>-암환자에게 재가기반 완화의료 서비스를 제공하였을 때 환자의 증상 조절이 더 잘 이루어짐. (Gomes, B., et al. (2013). "Effectiveness and cost-effectiveness of home palliative care services for adults with advanced illness and their caregivers." Cochrane Database of Systematic Reviews(6).)
연구 대상	암에 대한 치료가 종결되지 않은 상태에서 거동에 어려움을 겪는 진행기 고형암 환자와 가족 보호자
연구 대상자 수 (환자수 기준)	- 전체 대상자(6개 기관): 396명, 본 기관(중재군) 연구대상자: 66명<br>- 중재군과 대조군에 각각 3개 기관이 참여하여 전체 396명의 환자를 등록할 예정임. 중재군과 대조군은 1:1로 각 군에서 198명씩 등록 예정임.<br>- 목표 대상자 산출의 이론적 근거: 본 연구에서 검증하고자 하는 재가관리 프로그램의 표본 수는 검정력에서 안정적이고 충분한 수의 대상자를 확보하기 위해 일차적 결과지표인 이분형 변수일 때를 기준으로 함. 본 연구의 일차적

	결과지표(Primary Endpoint)는 연구 등록 후 6개월 이내의 계획되지 않은 입원 여부로 중재군과 대조군에서의 계획되지 않은 입원 대상자의 비율에 근거한 오즈비를 보여 줌으로써 효과차이를 보이고자 함. 국외에서 유사하게 시행된 디자인의 선행 중재연구가 부재하나 문헌 고찰을 통하여 파악하였을 때에 중재군과 대조군 간 중재에 따른 차이는 약 20% 정도로 (재택의료군이 20% 작음) 가정할 수 있겠음. 양 군의 기관 수가 각각 3개이고 기관별 수집되는 연구대상자의 집단 효과(cluster effect)를 고려하기 위해 GEE를 사용하여 모수를 추정할 예정이며, 두 군의 비교성 확보를 위해 기타 교란요인을 고려한 이분형 결과변수의 효과비교모형인 로지스틱회귀모형을 기준으로 분석할 예정임. 위의 분석 방법을 고려할 때, 검정력(Power) 80%, 1종 오류를 제어하기 위한 유의수준 0.05, 계획되지 않은 입원 수의 중재 전후 변화의 차이를 20%로 (재택의료군이 20% 작음) 가정한 조건에서 필요한 연구대상자 수는 중재군:대조군을 1:1로 배정 시에 최소 표본수는 중재군 3개 cluster 총 198명, 대조군 3개 cluster 총 198명, 전체 396명임. (within-cluster coefficient=0.015 가정). 최대 15% 탈락률을 가정하였을 때 최종 수집 될 연구대상자 수는 각 cluster별 약 57명으로 총 연구대상자 342명이 수집될 예정이며 예상되는 최소 검정력은 78.8%로 예상됨.(PASS 2022, v22.0.2 사용)<br>- 중재군으로 총 3개 기관 (서울대학교병원, 중앙대학교병원, 동국대학교 일산병원), 대조군으로 총 3개 기관 (경희대학교병원, 분당차병원, 분당서울대학교병원)이 참여함.
취약한 연구대상자	해당사항 없음
연구 계획	**1. 대상자 모집**<br>- 연구 참여기관의 외래나 병동, 공용게시판에 안내문을 공고하고, 공동연구자의 외래나 병동에서 연구 대상 기준이 되는 환자 및 보호자에게 연구를 설명하고 동의시 등록 계획임.<br><br>**2. 임상시험 내용**<br>1) 중재군(재택의료 제공)<br>- 중재군 기관에서는 의사, 간호사, 사회복지사로 구성된 재택의료팀을 구성함. 의사와 사회복지사는 기존 환자 진료와 상담에 참여하던 인력이 겸직 가능하며, 간호사는 재택의료 전담간호사로 지정함.<br>- 입원환자는 퇴원 예정일로부터 일주일 이내에 재택의료팀 간호사가 초기평가를 수행하고 재가돌봄 교육자료를 제공하고 재가돌봄 교육을 시행함. 외래 환자는 외래 방문일에 재택의료팀 간호사가 초기평가를 수행하고 재가돌봄 교육자료를 제공하고 재가돌봄 교육을 시행함.<br>- 연구 등록 2주 이내에 재택의료팀 의료진(의사 또는 간호사)이 환자의 집에 방문하여 재가환경을 평가하고, 재가 환경에 맞는 재가돌봄을 교육함. 환자에게 제공되는 재가돌봄 교육에는 약제 복용방법 교육, 발생 가능성이 있는 증상 관련 교육, 돌봄목표 수립이 포함됨.<br>- 재택의료팀은 월 1회 다학제 미팅을 시행하여 재택의료팀 등록 환자 관련

	정보를 공유하고, 주기적 재평가를 통해 재가관리 플랜을 재설정함.<br>- 첫 가정방문 이후에는 재택의료팀 간호사가 2주 주기로 전화 또는 문자로 환자와 보호자에게 연락하여 환자의 재가관리 상태를 점검함. 환자의 증상 변화에 따라 재택의료팀 의사에게 상황을 노티하여 약제 조절, 외래 및 입원 일정 조정 등을 제공함.<br>- 재택의료를 받는 환자에게는 평일 9A-5P에 연락 가능한 전화번호를 제공하여 재택의료팀 간호사가 해당 전화를 받아 필요한 상황에서 상담과 교육을 제공함.<br>- 재택의료 외 기존의 입원, 외래, 응급실, 가정간호, 지역사회 의료기관 방문 의료서비스는 기존처럼 이용함.<br>2) 대조군<br>- 연구 참여에 동의한 환자를 대상으로 입원환자는 퇴원 1주일 전, 외래 환자는 외래 방문일에 연구간호사가 환자에게 재가돌봄 교육자료를 제공함.<br>- 재택의료 외 기존의 입원, 외래, 응급실, 가정간호, 지역사회 의료기관 방문 의료서비스 이용을 지속함.<br><br>3. 분석 방법<br>- 중재군/대조군 참여기관에서 각각 모집한 환자를 추적관찰하여 추적 종료 후 대상자에 대한 결과변수 비교 분석을 시행함.
중지 기준	1. 환자 또는 보호자가 재택의료 중재 중단을 원하는 경우<br>2. 연구 대상자가 사망하는 경우<br>3. 의료기관에 4주 초과로 입원하는 경우<br>4. 해당병원 혈액종양내과 진료를 종료하였거나 추적관찰이 중단된 경우<br>5. 입원형 호스피스 혹은 가정형 호스피스를 이용하는 경우<br>6. 연구자 판단에 따라 임상시험 참여 중단이 연구 대상자에게 최선인 경우<br>7. 연구 대상자가 임상시험 담당의사에게 협조하지 않거나 임상시험 담당의사의 지침을 따르지 않는 경우<br>8. 규제당국 또는 윤리위원회/임상연구심사위원회가 본 시험을 중단시키는 경우<br><br>* 연구 중단한 환자에 대한 연구 종료에 대한 것은 이하와 같음.<br>- 1, 3, 4, 5, 6, 7, 8번 사유: 등록 후 12개월 혹은 동의 철회 시까지 의무기록 조사, 2차 자료 연계(18개월 포함)를 시행하며, 추가 설문 응답에 대해서는 동의 확인하여 동의하면 진행<br>- 2번 사유: 등록 후 12개월 시점까지 설문, 의무기록 조사는 불가, 2차 자료 연계를 시행함<br><br>*연구 동의철회를 한 경우에는 기존 자료의 연구 활용 가능 여부를 확인하여 동의하지 않는 경우 기존에 수집된 자료를 폐기한다.

선정기준	환자와 보호자가 모두 선정기준에 부합하여야 연구 대상이 됨.<br>1. 환자<br>① 진행기 고형암 진단 보유 (ICD-10 코드 C00-C70)<br>①-1. 암 치료를 받고 있거나 계획하는 자<br>② 다음 중 하나의 조건에 해당하는 자<br>②-1. ECOG performance status 2로 평가되는 자<br>②-2. ECOG performance status 1이면서 70세 이상인 자<br>③ 집에서 지내기를 희망하는 자<br>④ 집에 가족 보호자가 상주하는 자<br>⑤ 연구에 참여하기를 희망하는 자<br>2. 보호자<br>① 환자의 가족<br>* 가족: 환자의 배우자(사실혼 관계 동거인 포함), 2촌 이내 존비속과 그 배우자, 형제자매와 그 배우자, 8촌 이내 친척과 그 배우자를 의미한다.<br>② 다음 중 하나의 조건에 해당하는 자<br>②-1. 환자와 함께 동거하는 자 (환자 가구원)<br>②-2. 환자와 동거하지는 않으나, 환자 집에 주 3회 이상 방문하는 자<br>③ 환자가 집에서 지내기를 희망하는 자<br>④ 의료진과 원활한 소통이 가능한자<br>⑤ 연구 참여를 희망하는 자
제외기준	환자와 보호자 중 한명이라도 제외기준에 부합하면 연구 제외 대상이 됨.<br>1. 환자<br>① 한글을 말하거나 듣거나 읽지 못하는 자<br>② 의사의 판단에 의해 의학적으로 건강 상태가 극도로 불량하여 본 연구를 수행할 수 없다고 판단되는 자<br>③ 해당 의료기관에서 방문이 불가능한 범위(의료기관 별로 거리 조건 사전에 지정)에 거주하는 자 (※)<br>④ 입원형 혹은 가정형 호스피스 완화의료 서비스를 이용한 적 있는 자<br>※ 대조군에서는 배제 조건에서 제외<br>⑤ 만 19세 미만인 자<br>2. 보호자<br>① 한글을 말하거나 듣거나 읽지 못하는 자<br>② 의사의 판단에 의해 의학적으로 건강 상태가 극도로 불량하여 본 연구를 수행할 수 없다고 판단되는 자<br>③ 만 19세 미만인 자
유효성 평가	1. 데이터 수집<br>1) 설문<br>- 중재군과 대조군 모두 연구 대상자 등록시점에 환자와 보호자에게 설문을 시행함.<br>- 중재군과 대조군에게 등록 3개월 시점에 추적 설문을 시행함.

	- 중재군에서는 등록 6개월 시점에 추가적인 설문을 시행함. 대조군에서는 3개월 시점에서 동의한 자에 한해 6개월 시점에 추가적인 설문을 시행함.<br><br>2) 의무기록 조사<br>- 등록 3, 6, 12개월 시점에 의료기관 의무기록조사를 시행하여 각 시점의 입원(입원 횟수, 입원일), 중증 의료이용(중환자실 입원일, 인공호흡기 적용일, 심폐소생술 시행 횟수), 응급실 이용 정보(응급실 방문 횟수)를 확인함.<br>- 등록 3, 6, 12개월 시점에 생존 여부를 확인하여 사망이 확인된 경우 사망 1달 이내의 의료이용(입원, 중증 의료이용, 응급실 이용)을 확인함.<br><br>3) 2차 자료 연계<br>- 2차 자료 연계에 동의한 환자들에 대해 모든 환자가 등록 12개월 이상 경과한 시점에 등록 후 12개월 시점까지의 입원, 응급실 이용 정보, 의료비, 중증 의료이용, 사망 여부, 호스피스 이용 여부와 생애말기 의료이용 종류를 확인함.<br>- 모든 환자가 등록 18개월 이상 경과한 시점에 등록 18개월 시점의 사망 여부, 호스피스 이용 관련 정보와 생애말기 의료이용 종류를 확인함.<br>- 2차 자료 연계에 동의한 환자에 대해 모든 환자가 등록 12, 18개월 이상 경과한 시점에 등록 12, 18개월 시점의 연명의료 법정 서식 (사전연명의료의향서, 연명의료계획서) 작성 여부와 작성 주체를 확인함.<br><br>2. 결과 변수<br>- 1차 평가 변수: 연구 등록 후 6개월간 계획되지 않은 입원의 수<br>*계획되지 않은 입원: 해당 기관에 급성기 치료 목적으로 입원한 경우 (항암치료를 위한 예정된 입원, 수술/시술을 위해 입원일자를 지정하여 지정한 입원 제외), 해당 의료기관이 아닌 타 의료기관에 입원한 경우, 요양병원에 4주 이하 입원한 경우<br>- 2차 평가 변수<br>■ 환자: 의료이용 (입원, 응급실), 중증의료이용 (중환자실 입원, 인공호흡기 적용), 생애 말 의료이용 (사망 1개월 이전 중환자실 입원, 인공호흡기 적용), 서비스 만족도(satisfaction with services), 삶의 질 (ESAS, EQ5D), 정서장애(PHQ9), 사전연명의료의향서 작성<br>■ 보호자: 서비스 만족도(satisfaction with services), 삶의 질(EQ5D), 돌봄 부담 (CRA-K), 정서장애(PHQ9), 돌봄 항목 수행 역량<br>■ 비용: 의료비용, 돌봄비용
안전성 평가	해당사항 없음
통계적 분석	결과 변수에 대하여 재택의료 중재군과 비교군, 각 참여 의료기관을 분석집단으로 하여 아래와 같이 통계적 분석을 시행함.

	연구대상자의 분석군은 최초 분류된 중재 여부에만 의존하는 최초 배정된 군에 따른 분석 (ITT; intention-to-treat)에 준한 군 분류를 원칙으로 하여 치료군의 효과를 보수적으로 추정할 수 있도록 하며, 추적관찰 중 동의 철회 또는 중도 탈락이 발생하는 경우 동의 철회 또는 중도 탈락 시점까지의 자료를 활용하고, 군별 탈락률에 대한 비교 분석 및 보고함<br>- 기저특성에 대한 기술통계<br>연속변수: 평균 (표준편차), 중앙값 (IQR)<br>이분형변수: 빈도 (%)<br>- 군간 차이에 대한 분석<br>군간 기저특성의 차이를 보정한 재택의료 중재의 효과를 평가하기 위하여 회귀모형을 이용하며, 관찰단위인 연구대상자들이 6개의 참여기관 내에 clustering 되어 있음을 고려한 분석을 시행함<br>이분형 변수(1차 평가 변수): 참여 기관 내 환자의 cluster를 고려하기 위하여 GEE를 사용하며, 로짓 연결함수(logit link function)를 매개로 하여 로지스틱 회귀모형을 기본 모형으로 재택의료 중재의 효과를 평가함<br>연속변수: 참여기관 내의 환자의 cluster를 고려하기 위하여 Mixed model을 이용하여 재택의료 중재의 효과를 평가함.<br>Count data: 참여기관 내의 환자의cluster를 고려하기 위하여 GEE를 사용하며, link function으로 로그 링크 함수(log link function)를 매개로 하여 변수의 분포에 따라 Poisson, negative binomial을 랜덤성분으로 적절히 사용한 회귀 모형을 통해 재택의료 중재의 효과를 평가함.
기대효과 및 예상결과	한국의 의료 상황에 적합한 중증환자에 대한 재택의료 모델을 개발하고 효과를 검증하여 국내 중증질환자 대상 재택의료 서비스의 확산을 위한 근거를 생성한다.

연구계획서

1. 연구 제목

(국문) 항암치료를 지속 중인 고형암 환자를 대상으로 재택의료를 제공하여 등록부터 6개월 이내 예정되지 않은 입원 감소 효과를 평가하기 위한 군집 비무작위 배정 연구자 주도 임상시험
(영문) A Cluster, Non-randomized Controlled Trial of the Effectiveness of a Korean Model for Home-based Care in Patients with Advanced Cancer

2. 연구의 실시기관 명칭 및 주소

실시기관 명칭	실시기관 주소
서울대학교병원	03080 서울특별시 종로구 대학로 101
분당서울대학교병원	13620 경기도 성남시 분당구 구미로173번길 82
동국대학교 일산병원	10326 경기도 고양시 일산동구 동국로 27
중앙대학교병원	06973 서울특별시 동작구 흑석로 102
분당차병원	13496 경기도 성남시 분당구 야탑로 59
경희대학교병원	02453 서울특별시 동대문구 경희대로 23

3. 연구책임자 및 공동연구자 성명 및 직명

1) 연구책임자: 조비룡 교수, 서울대학교병원 가정의학과/공공진료센터
2) 내부 공동연구자 (가나다 순)

이름	소속	직위
김계형	서울대학교병원 공공진료센터	진료부교수
김민선	서울대학교병원 소아청소년과/공공진료센터	임상조교수
김소희	서울대학교병원 공공진료센터	연구코디네이터
김범석	서울대학교병원 혈액종양내과/완화의료・임상윤리센터	임상교수
배윤경	서울대학교병원 완화의료・임상윤리센터	연구원
신정미	서울대학교병원 공공진료센터	진료조교수
유신혜	서울대학교병원 완화의료・임상윤리센터	임상조교수
이가영	서울대학교병원 공공진료센터	연구코디네이터
이선영	서울대학교병원 공공진료센터	임상조교수
이중엽	서울대학교 의과대학 예방의학교실	교수
장민설	서울대학교병원 공공진료센터	임상강사
한현정	서울대학교 병원 사회복지팀	사회복지사
황인영	서울대학교병원 공공진료센터	진료조교수

3) 외부 공동연구자 (가나다 순)

이름	소속	직위
강버들	분당차병원 혈액종양내과	교수
김도연	동국대학교 일산병원 혈액종양내과	교수

김달용	동국대학교 일산병원 혈액종양내과	교수
김유정	분당서울대학교병원 혈액종양내과	교수
백선경	경희대학교병원 혈액종양내과	교수
심진아	한림대학교 인공지능융합학부	교수
오충렬	중앙대학교병원 혈액종양내과	교수
황인규	중앙대학교병원 혈액종양내과	교수

3) 연구담당자:

■ 유신혜 교수, 서울대학교병원 완화의료/임상윤리센터 (ifi1024@gmail.com)

■ 이선영 교수, 서울대학교병원 공공진료센터 (sy2376@gmail.com)

4) 임상시험용 의약품 관리약사 / 임상시험용 의료기기 관리자: 해당사항 없음

4. 연구 의뢰기관: 해당사항 없음

5. 연구비 지원기관 명칭 및 주소:

1) 명칭: 한국보건의료연구원

2) 주소: 서울특별시 중구 퇴계로 173 남산스퀘어빌딩 7층

6. 예상연구기간: IRB 승인일 ~ 2026년 12월 31일

7. 연구 대상 질환: 진행암환자

임상시험의 구체 내용

1. 임상연구의 명칭

- 항암치료를 지속 중인 고형암 환자를 대상으로 재택의료를 제공하여 등록부터 6개월 이내 예정되지 않은 입원 수의 감소 효과를 평가하기 위한 군집 비무작위 배정 연구자 주도 임상시험

2. 서론

가. 연구의 배경

○ 중증질환자 돌봄의 미충족 의료영역

- 의학의 발전에 따라 중증질환자에 대한 치료의 강도는 점점 강해지고 있다. 새로운 항암제가 지속개발되며 진행암환자 생존기간이 길어지고 있으며, 각종 의료기기의 발달로 완치는 되지 않으나 장기간 치료받으며 생존하는 비암 중증질환자도 증가하고 있다.
- 의료기술이 발달하며 각종 생명유지를 위한 의료기기가 소형화되고 단순화되어 의료기기에 의존하여 생명을 영위하는 환자 역시 증가하고 있다. 그러나 이렇게 만성화된 상태로 살아가는 중증질환자에 대한 의료적 돌봄(보존적 치료, supportive care)은 부족하여 미충족 의료영역으로 존재한다.

○ 진행암 환자에 대한 부족한 의료적 돌봄

- 의료 기술의 발전과 중증질환에 대한 보장성 강화로 암환자에 대해 강도 높은 치료가 제공되며 진행암 환자의 생존기간은 증가하고 있다. 그러나 암에 대한 치료에 의료적 관심이 집중되며 진행암 환자에 대한 돌봄은 부족한 수준이다.
- 진행암 환자, 특히 고형암 환자는 표준 항암치료를 받으며 주로 외래 기반 항암치료를 받게 된다. 이 때 외래 기반으로 표준치료에 더해 조기 완화의료를 받는 것이 효과적임이 국외 결과에서 입증되었으나, 국내에서 조기 완화의료는 아직 제대로 도입되지 못하였다.
- 신체기능(Performance status)이 저하된 진행암 환자가 말기로 판단되면 호스피스 완화의료를 받을 수 있다. 그러나 입원형/가정형 호스피스 완화의료 서비스는 항암치료를 완전히 종결한 상태에서만 이용이 가능하여, 국내에서 실제로 암환자가 호스피스를 이용하게 되는 시기는 해외에 비해 상당히 늦은 편이다.
- 진행암 환자는 암에 대한 치료 중 점차 신체기능이 저하되어 독립적으로 거동이 어려운 재가상태(homebound)가 될 수 있다. 이러한 경우에는 국외에서는 항암치료를 잘 시행하지 않으나, 국내에서는 불량한 신체기능 (ECOG performance status 2 이하)로도 항암치료를 지속하는 진행암 환자들이 상당한 규모이다.
- 또한 대형병원으로 환자쏠림이 발생하며 강도 높은 항암치료, 방사선치료 등을 받는 환자들에게 적절한 의료적 돌봄까지 제공하기는 어려운 것이 현실이다. 이들은 주로 요양병원/2차병원을 이용하며 많은 의료비를 지출하고 있고, 돌봄 희망장소가 병원이 아니어도 다른 대안이 없어서 집에서의 돌봄이 이루어지지 못하고 있다.
- 의료적 돌봄이 필요한 환자들이 상급종합병원에 장기간 입원하면 가용 가능한 급성기 병상이 줄어들어 급성기 치료가 필요한 환자가 적절한 치료를 받지 못할 우려가 있다. 그러나

이들이 다른 병원을 이용하더라도 항암치료 중이기 때문에 사실상 일차의료 주치의가 없는 국내에서는 작은 문제에도 빈번하게 기존 상급종합병원의 응급실을 이용하게 되어 이는 또 응급실 과밀화를 야기하게 된다. 이에 점차적으로 와상(bed-ridden state) 상태로 접어드는 재가환자이지만 호스피스 완화의료 서비스를 이용하지 못하는 진행암 환자들의 의료적 돌봄은 현재 사각지대 미충족 의료영역이다.

나. 연구의 근거

○ 재택의료는 재가환자의 삶의 질을 향상시키고 비용효과적임.

- 노인인구가 증가하고 의료기술이 발전함에 따라 지속적인 의료서비스가 필요하나 각종 의학적, 사회적 문제로 인해 의료기관 방문이 어려운 재가환자군이 증가하고 있다. 그래서 이들을 대상으로 한 재택의료 서비스(home-based medical care, HBMC)가 개발되어 시행되고 있다.
- 재택의료는 기존의 급성기 치료나 요양병원, 요양원 등 돌봄과 의료서비스를 동시에 제공하는 거주시설과 다르게 환자가 기존에 거주하던 집에서 지내며 의료인이 환자의 거주 장소로 방문하여 서비스를 제공한다는 특성이 있다.
- 환자가 기존에 삶을 영위하던 공간에 지속 거주할 수 있으므로 삶의 질이 향상되고, 서비스를 제공하기 위한 건물 등 의료시설이 많이 필요하지 않아 비용이 적게 든다는 장점이 있어 전세계적으로 재택의료의 대상과 범위는 점점 더 확대되고 있다.

○ 비중증질환 재가환자에 대한 재택의료 중재는 의료이용을 줄이고 삶의 질을 향상시킴.

- 해외에서 진행된 재택의료 중재 연구의 대상은 연약한 노인 환자(frail elderly), 복잡한 만성 질환자(치매, 심한 정신질환, 당뇨 등 만성질환을 여러 개 가진 환자), 거동이 불편하거나 장애(disability)가 있는 환자 등 일차의료기관의 지속적인 관리가 필요하나 거동이 자유롭지 않은 재가환자이다.
- 비중증질환 재가환자에 대하여 재가기반 일차의료 중재(home-based primary care, HBPC)의 의료이용 감소와 삶의 질 향상의 효과는 알려져 있다. 비중증질환 재가환자에 대한 지속적인 재택의료 중재는 18개월 이상 추적관찰을 했을 때에도 의료이용과 총 의료비용을 줄이는 긍정적인 효과가 증명되었다.

○ 중증질환자에 대한 전환기 치료(transitional care)를 포함한 재택의료 중재는 효과적임.

- 급성기 치료병원에서 심부전, 만성폐쇄성호흡기질환 등 급성악화 가능성이 높은 만성 중증질환 퇴원환자를 대상으로 전환기 치료의 일환으로 재택의료를 제공하면 재입원을 줄이고 의료비용을 줄이는 긍정적인 효과가 있었다.
- 암, 심부전, 만성폐쇄성호흡기질환 등 완치가 불가능하고 기대여명이 제한되는 중증질환자에게 재가기반 완화의료(home-based palliative care) 제공은 생존 기간을 짧게 하지 않고 증상 조절에 효과적이며 환자의 건강관련 삶의 질을 높이는 긍정적인 효과가 증명되었다.

3. 임상연구의 목적

- 거동에 어려움이 있는 진행암 환자와 가족 보호자에게 재가돌봄 교육을 제공하고, 의료진의 가정방문, 주기적 상태 점검을 포함하는 재택의료 중재 프로그램을 제공하는 것이 6개월 이내 예정되지 않는 재입원을 감소시키는데 미치는 효과를 검증하고자 한다.

4. 임상연구의 디자인

가. 임상연구의 단계

- Phase Ⅲ

나. 시험대상자

- 항암치료를 지속 중인 기대여명이 제한되고 거동이 어려운 고형암 환자

5. 대상자 선정, 제외 기준

- 본 연구의 대상자는 환자와 보호자가 각각 선정기준과 제외기준을 만족한 환자를 대상으로 한다.

가. 대상자 선정 기준

1) 환자

① 진행기 고형암 진단 보유 (ICD-10 코드 C00-C70)

①-1. 암 치료를 받고 있거나 계획하는 자

② 다음 중 하나의 조건에 해당하는 자

②-1. ECOG performance status 2로 평가되는 자

②-2. ECOG performance status 1이면서 65세 이상인 자

③ 집에서 지내기를 희망하는 자

④ 집에 가족 보호자가 상주하는 자 또는 주 3회 이상 방문하는 자

⑤ 연구에 참여하기를 희망하는 자

2) 보호자

① 환자의 가족

* 가족: 환자의 배우자(사실혼 관계 동거인 포함), 2촌 이내 존비속과 그 배우자, 형제자매와 그 배우자, 8촌 이내 친척과 그 배우자를 의미한다.

② 다음 중 하나의 조건에 해당하는 자

②-1. 환자와 함께 동거하는 자 (환자 가구원)

②-2. 환자와 동거하지는 않으나, 환자 집에 주 3회 이상 방문하는 자

③ 환자가 집에서 지내기를 희망하는 자

④ 의료진과 원활한 소통이 가능한자

⑤ 연구 참여를 희망하는 자

나. 대상자 제외 기준

- 환자와 보호자 중 한명이라도 제외기준에 부합하면 연구 제외 대상이 된다.

1) 환자

① 한글을 말하거나 듣거나 읽지 못하는 자

② 의사의 판단에 의해 의학적으로 건강 상태가 극도로 불량하여 본 연구를 수행할 수 없다고 판단되는 자

③ 해당 의료기관에서 방문이 불가능한 범위(의료기관 별로 거리 조건 사전에 지정)에 거주하는 자 (※)

④ 입원형 혹은 가정형 호스피스 완화의료 서비스를 이용한 적 있는 자

※ 대조군에서는 배제 조건에서 제외함.

⑤ 만 19세 미만인 자

2) 보호자

① 한글을 말하거나 듣거나 읽지 못하는 자

② 의사의 판단에 의해 의학적으로 건강 상태가 극도로 불량하여 본 연구를 수행할 수 없다고 판단되는 자

③ 만 19세 미만인 자

6. 대상자 수 산출

- 본 연구에서 검증하고자 하는 재가관리 프로그램의 표본 수는 검정력에서 안정적이고 충분한 수의 대상자를 확보하기 위해 일차적 결과지표인 이분형 변수일 때를 기준으로 함. 본 연구의 일차적 결과지표(Primary Endpoint)는 연구 등록 후 6개월 이내의 계획되지 않은 입원 여부로 중재군과 대조군에서의 계획되지 않은 입원 대상자의 비율에 근거한 오즈비를 보여 줌으로써 효과차이를 보이고자 함. 국외에서 유사하게 시행된 디자인의 선행 중재연구가 부재하나 문헌 고찰을 통하여 파악하였을 때에 중재군과 대조군 간 중재 전후 변화의 차이는 약 20% 정도(재택의료군이 20% 작음)로 가정할 수 있겠음. 양 군의 기관 수가 각각 3개이고 기관별 수집되는 연구대상자의 집단 효과(cluster effect)를 고려하기 위해 GEE를 사용하여 모수를 추정할 예정이며, 두 군의 비교성 확보를 위해 기타 교란요인을 고려한 이분형 결과변수의 효과비교모형인 로지스틱회귀모형을 기준으로 분석할 예정임. 위의 분석 방법을 고려할 때, 검정력(Power) 80%, 1종 오류를 제어하기 위한 유의수준 0.05, 계획되지 않은 입원 수의 중재에 따른 차이를 20%로 (재택의료군이 20% 작음) 가정한 조건에서 필요한 연구대상자 수는 중재군:대조군을 1:1로 배정 시에 최소 표본수는 중재군 3개 cluster 총 198명, 대조군 3개 cluster 총 198명, 전체 396명임. (within-cluster coefficient=0.015 가정).
- 이 때, within-cluster coefficient(Intercluster coefficient, ICC)의 경우 문헌 고찰을 통해 확인된 유사 연구에 따라 ANOVA 추정법을 활용하여 추정하였으며 산출식은 다음과 같음(Sheng Wu 등(2012), Chen CY 등(2015))

$$\widehat{ICC} = \frac{MSB = MSW}{MSB + (n_A - 1)MSW}$$

여기서 $n_A = \frac{1}{k-1}\left(N - \sum n_i^2/N\right)$, $MSB = \frac{1}{k-1}\left(\sum Z_i^2/n_i - (\sum Z_i)^2/N\right)$, $MSW = \frac{1}{N-k}\left(\sum Z_i - \sum Z_i^2/n_i\right)$ 이며, k는 집단(cluster) 개수, N은 전체 연구 대상자 수, n_i는 집단 i의 연구대상자 수, Z_i는 집단 i의 결과지표 발생자 수로 정의되며, Chen CY 등(2015)의 연구에서 그룹별 발생률을 발췌하여 본 연구에 적용하였음

k	N	n_1,n_2	Z_1,Z_2	n_A	MSB	MSW	ICC
6	396	198,198	40,20	39.6	0.20202	0.127946	>0.01441

검정력 (Power)	Sample size	# of cluster	Allocation	Average Cluster size	Effect size	ICC	Alpha
0.72584	264	4	ri(1)	66	0.2	0.01	0.05
0.66263	264	4	ri(1)	66	0.2	0.015	0.05
0.5826	264	4	ri(1)	66	0.2	0.02	0.05
0.72584	264	4	ri(1)	66	0.2	0.01	0.05
0.83007	396	6	ri(1)	66	0.2	0.015	0.05
0.75675	396	6	ri(1)	66	0.2	0.02	0.05
0.88014	396	6	ri(1)	66	0.2	0.01	0.05
0.83007	396	6	ri(1)	66	0.2	0.015	0.05
0.8658	528	8	ri(1)	66	0.2	0.02	0.05
0.95162	528	8	ri(1)	66	0.2	0.01	0.05
0.92004	528	8	ri(1)	66	0.2	0.015	0.05
0.92905	660	10	ri(1)	66	0.2	0.02	0.05

ICC: Intercluster Correlation
ri(1) : 중재군, 대조군 할당 비율 1:1

- 기관별 최대 15% 탈락률을 가정하였을 때 최종 수집 될 연구대상자 수는 각 기관별 약 57명으로 총 연구대상자 342명이 수집될 예정이며 예상되는 최소 검정력은 78.8%로 예상됨.(PASS 2022, v22.0.2 사용)
- 본 연구를 위해 중재군으로 총 3개 기관 (서울대학교병원, 중앙대학교병원, 동국대학교 일산병원), 대조군으로 총 3개 기관 (경희대학교병원, 분당차병원, 분당서울대학교병원)이 참여하며, 기관당 최소 66명의 연구대상자를 수집할 예정으로 총 연구대상자 396명을 모집할 경우 효과차이를 검정할 충분한 검정력이 산출될 것으로 예상되며, 연구대상자 모집 및 통계분석 후 검정력에 대한 산출 및 기술 예정임

7. 임상연구 기간

- 약 3년 6개월 (모집기간 약 2년, 중재 제공 및 평가기간 약 1년 6개월)

가. 대상자 모집기간

- 약 24개월 (2022년 6월 ~ 2024년 5월)

나. 재택의료 중재 제공기간

- 재택의료 중재 프로그램 등록 후 1년
- 임상시험 참여자 중 중재군은 등록 후 1년간 재택의료 프로그램을 제공받을 것이다.

다. 추적기간

- 임상시험 등록 후 18개월 (중재 제공기간 포함)
- 임상시험 등록 후 재택의료 중재 제공과 추적이 동시에 이루어진다.

8. 임상시험 환자 등록 및 초기평가

가. 환자 모집

- 임상시험 참여기관의 외래나 병동, 공용게시판에 안내문을 공고하고, 공동연구자의 외래나 병동에서 기준이 되는 환자 및 보호자에게 연구를 설명하고 동의시 등록한다.
- 각 참여기관 외래 및 입원 환자 중 선정기준의 항목을 모두 만족하고, 제외 기준의 항목들은 모두 해당사항이 없을 경우에 환자 및 보호자에게 연구에 대한 자세히 설명하고, 연구 참여에 대하여 환자가 동의할 경우에 동의서를 작성하도록 한다. 보호자에게도 별도로 동의서를 작성하도록 한다.
- 임상시험 참여에 대한 동의서 내에 2차 자료 수집을 위해 환자의 고유식별정보(주민등록번호)를 수집하고 정보열람 및 이용하는 것에 대한 동의 항목 (보험공단 또는 건강보험심사평가원 자료, 통계청 사망 자료, 국가생명윤리정책원 자료 등)를 별도로 두어, 별도로 동의 여부를 체크한다.

나. 초기 평가 및 교육

- 연구에 등록한 환자와 보호자를 대상으로 연구 간호사(중재군의 경우 재택의료팀 간호사)가 1차 설문평가와 교육을 시행한다.
- 입원 환자는 퇴원 예정일로부터 일주일 이내 시점에 수행하고 외래 환자는 환자의 외래 방문일에 수행한다.
- 임상시험 참여자의 초기 연령, 성별, 인구학적 정보, 거주지, 보험종류, 가구 경제 상태, 가구 구성, 주 돌봄 제공자, 연명의료계획, 복지서비스, 과거력, 질병명, 보유 의료기기, 증상, 삶의 질, 정서평가 등과 같은 항목이 포함된다.
- 임상시험 대상자의 보호자에 대한 초기 평가에는 연령, 성별, 인구학적 정보, 환자와의 관계, 돌봄 정보, 삶의 질, 정서평가, 돌봄부담, 돌봄 준비, 돌봄제공자 역량, 돌봄 보람 등의 항목이 포함된다.
- 초기 평가 시점에 연구간호사가 재가돌봄 교육자료(개발 예정)를 제공한다. 재가돌봄 교육자료의 내용에는 이하 내용이 포함된다.

구분	내용
통증 관리	진통제의 종류와 복용 방법
증상 관리	해당 환자에게 발생할 수 있는 증상의 종류와 가정에서의 조절 방법
재가 관리	재가환경 관리 방법

9. 재택의료 중재

가. 재택의료팀 구성

- 중재군 기관에서는 의사, 간호사, 사회복지사로 구성된 재택의료팀을 구성한다. 재택의료팀 의사는 재택의료 전담의사 또는 혈액종양내과 의사가 겸직한다.
- 재택의료팀 간호사는 중재군 기관에서 간호사를 신규 채용하여 3개월 이상의 재택의료 중재 교육을 시행한 후 재택의료 전담간호사로 지정한다.
- 재택의료팀 사회복지사는 해당 기관에서 기존에 근무하던 사회복지사가 겸직한다.

나. 등록 시 재택의료 대상자 추가평가 및 교육

- 재택의료 중재 프로그램에 등록된 환자에게는 등록 시점에서 임상 시험 대상자에 대한 초기 평가 외에 재택의료팀 간호사가 약물 투약력, 증상 조절 정도, 보유 의료기기에 대한 추가 평가를 수행한다. 평가 항목 중 병원에서 평가하기 어려운 항목 (약물 투약력 등)에 대해서는 가정방문 평가 시점에 추가 평가한다. 복지서비스 등 일부 항목에 대해서는 재택의료팀 사회복지사가 평가하는 것이 가능하다.
- 재택의료 중재 대상자의 추가 평가 시에는 질병 관련 증상, 보유 의료기기, 약물 투약력에 대해 추가 조사하고 평가한다.
- 추가 평가와 함께 재택의료팀 간호사가 재가돌봄 교육자료(개발 예정)를 제공하고 환자와 보호자 대상으로 15분이 소요되는 재가돌봄 교육을 시행한다. 중재간호사가 교육하는 재가돌봄 교육의 내용에는 이하 내용이 포함된다.

구분	내용
증상관리	해당 환자에게 발생할 수 있는 증상의 종류와 가정에서의 조절 방법
약물 복용	해당 환자에게 처방된 약물의 종류와 복용 방법
의료기기 관리 방법	환자가 의료기기를 보유하고 있다면 해당 의료기기의 관리 방법
의료진 보고가 필요한 항목	의료진 보고가 필요한 증상의 종류와 강도

다. 가정방문 평가 및 교육

- 초기 평가 수행 후 2주 이내에 재택의료팀 의료진 (의사 또는 간호사)가 환자와 보호자가 둘 다 있는 시간에 환자의 집에 방문하여 평가와 교육을 시행한다.
- 재택의료팀 의료진이 환자의 집에 방문하여 평가하는 항목은 이하와 같다.

구분	내용
재가환경	환자가 거주하는 집 (방, 거실, 화장실)의 위험 요인<br>- 낙상 위험이 있는지, 약물과 의료기기가 적절히 관리되고 있는지
약물 복용	해당 기관에서 처방된 약물 이외에 환자가 보유하고 있거나 복용하고 있는 약물의 종류와 복용 방법
의료기기 관리 상태	환자가 의료기기를 보유하고 있다면 집에서 해당 의료기기를 적절하게 사용하고 있는지 확인
증상 및 식이섭취량	환자가 호소하고 있는 증상 및 식이 섭취량을 평가함.

전신 활동도	ECOG performance status, CFS score
활력 징후	등록 후 첫 방문 시 혈압, 맥박, 호흡수, 체온, 산소포화도 등

- 재택의료팀 의료진이 환자의 집에 방문하여 교육하는 항목은 이하와 같다. 초기 교육의 내용에 더해 재가 환경 조정이 필요한 항목이 있는 경우 관련 내용을 교육한다.

구분	내용
재가 환경 조정	환자가 거주하는 집에 위험 요인이 있다고 평가되는 경우, 재가 환경을 조정할 수 있는 방법 논의<br>- 미끄럼 방지 매트리스 적용, 가정용 산소 비상용 전원 마련 등
증상관리	해당 환자에게 발생할 수 있는 증상의 종류와 가정에서의 조절 방법
약물 복용	해당 환자에게 처방된 약물의 종류와 복용 방법
의료기기 관리 방법	환자가 의료기기를 보유하고 있다면 해당 의료기기의 관리 방법
의료진 보고가 필요한 항목	의료진 보고가 필요한 증상의 종류와 강도

라. 다학제 팀미팅

- 재택의료팀은 월 1회 재택의료팀 의사, 간호사, 사회복지사가 참여하는 다학제 미팅을 시행하여 재택의료팀 등록 환자 관련 정보를 공유하고, 신규 등록된 환자의 돌봄계획을 수립하며, 주기적 재평가를 통해 돌봄계획을 재설정한다.
- 재택의료 중재 프로그램에 등록되어 초기 평가를 시행하고, 가정방문 평가까지 완료된 환자는 다음 회수의 다학제 팀미팅에서 돌봄계획을 수립하여 해당 계획에 따라 이후 재택의료팀 재가관리를 수행한다.
- 다학제 팀미팅에서 수립하고, 주기적으로 재설정하는 돌봄계획에는 이하 내용이 포함된다.

구분	내용
돌봄목표 수립	환자와 보호자(돌봄제공자)의 평가 결과를 바탕으로, 환자의 집에서의 돌봄 목표를 수립한다.<br>- 집에서 지내기 희망하는 기간, 입원치료를 희망하는 상황 결정<br>- 연명의료에 대한 환자와 보호자의 의사 확인
증상관리	해당 환자에게 발생할 수 있는 증상의 종류와 대처방법을 공유한다.<br>- 1. 기존에 약물을 보유하고 있는 증상에 대해서는 재택의료팀 간호사가 약물 복용 방법을 교육하고 증상 호전 여부를 관찰한다.<br>- 2. 새로 발생한 증상이나 기존 약물 복용으로 조절되지 않는 증상에 대해서는 재택의료팀 간호사가 재택의료팀 의사에게 연락하여 추가 약물 처방 여부를 상의한다.<br>- 3. 추가 약물 처방이 필요한 등 의사의 진료 필요한 경우에는 재택의료팀 간호사가 재택의료팀 의사와 상의하여 외래 일정을 조정하여 환자와 보호자에게 안내한다.<br>- 4. 입원이 필요하거나 응급실 방문이 필요한 경우에는 재택의료팀 간호사가 환자와 보호자에게 연락하여 안내한다.

재가관리 계획	2주 주기의 정기 관리 외 추가적인 관리나 방문이 필요한지 여부를 결정한다.<br>- 증상관리, 약물복용 등 교육한 항목에 대해 추가 교육이 필요하고 환자와 보호자가 추가 방문을 원하는 경우 재택의료팀 간호사가 추가 방문하여 추가 교육을 시행한다.<br>- 증상 악화 가능성이 높은 환자에 대해서는 돌봄계획 수립 후 1주 이내에 연락하여 상태 변화를 파악한다.
의료진 보고가 필요한 항목	재택의료팀 간호사의 정기 관리 중 재택의료팀 의사에게 보고가 필요한 증상 또는 발생 가능한 이벤트를 공유한다. 공통적으로 포함되는 항목은 이하와 같다.<br>- 약물 복용 중에도 NRS 7점 이상의 통증 지속<br>- 약물 복용 중에도 조절되지 않는 증상 지속<br>- 의료기기 사용 중 간호사가 평가 및 교육 어려운 문제 발생<br>- 돌봄제공자의 돌봄환경 변화 (지속 돌봄 제공이 어려운 등)

마. 재가관리

- 다학제 팀미팅에서 환자의 돌봄계획이 수립된 이후에는 재택의료팀 간호사가 2주 주기로 전화로 환자/보호자에게 연락하여 환자의 재가관리 상태를 점검한다. 주기적 재가관리 점검에서 재택의료팀 간호사가 점검하는 항목은 다음과 같다.

구분	내용
전신 활동도	ECOG performance status, CFS score
증상관리	증상 유무와 이전 평가에 비해 악화, 완화 여부
약물 복용	약물을 처방받은 대로 복용하고 있는지 확인<br>- 처방 받은대로 복용하고 있지 않다면 이유 확인
의료기기 상태	환자가 의료기기를 보유하고 있다면 해당 의료기기가 이상 없이 작동되고 있는지 확인
기타 사항	기타 환자와 보호자가 의료진과 상의하고 싶은 사항이 있다면 해당 내용에 대한 확인

- 재택의료팀 간호사는 이상의 항목들을 확인하여 필요시 재택의료팀 의사에게 평가 결과를 노티하고 약제 조절, 외래 및 입원 일정 조정 등을 제공한다. 즉시 대응이 필요한 중증 증상 발생시에는 응급실 방문을 권유한다.
- 재택의료를 받는 환자에게는 평일 9A-5P에 재택의료팀 간호사에게 연락 가능한 전화번호를 제공하여 정기 관리 이외에 사건 발생시 환자와 보호자가 재택의료팀 간호사에게 연락하여 상의할 수 있도록 한다.
- 재가관리를 제공하는 중에는 월 1회 이루어지는 다학제팀미팅에서 환자 평가 결과를 팀 내부에서 공유하고 필요시 재가돌봄 계획을 수정한다.
- 재가관리 중 환자가 급성기 병원이나 요양병원에 입원하는 경우 관리를 중단하고, 환자가 집으로 퇴원한 이후 재가관리를 재개한다. 재가관리를 재개할 때는 재택의료팀 간호사가 환자 초기평가 항목 중 변화된 항목을 재평가하여 다학제미팅에서 공유하고 재가돌봄계획을 재수립한다. 환자가 해당병원에 입원한 경우에는 재택의료팀 간호사가 퇴원 전 환자

병실에 방문하여 환자 상태를 재평가하고, 환자가 이외 병원에 입원한 경우에는 전화로 연락하여 환자 상태를 재평가한다.

- 재가관리 중 2회 초과(4주 초과)로 환자 또는 보호자와 연락이 되지 않거나, 환자 또는 보호자가 주기적인 연락을 거부하는 경우 재가 관리를 중단한다.
- 재택의료 외 기존의 입원, 외래 서비스와 응급실, 가정간호, 지역사회 의료기관 방문 의료서비스는 이용을 유지한다.

10. 대조군

- 대조군 기관에서 등록된 환자는 초기평가 후 재택의료 교육자료를 제공받고 중재에 해당하는 중증환자 재택의료 프로그램 외 기존의 입원, 외래 서비스와 응급실, 가정간호, 지역사회 의료기관 방문 의료서비스 이용을 유지한다.
- 대조군에 등록된 환자가 중재군 의료기관으로 추적관찰 의료기관을 옮기게 되는 경우 임상시험에서는 탈락한다.

11. 변수 조사 및 수집

가. 설문조사 및 연구자 면접 평가

- 중재군과 대조군 환자에 대하여 등록 시점에 연구자 면접 평가를 시행하고, 등록 시점, 3개월, 6개월에 설문 평가를 수행한다. 설문 평가는 평가 시점에서 +- 1달 이내에 환자와 보호자가 외래 또는 입원을 위해 병원을 방문하면, 연구간호사가 외래 또는 입원 병실에 방문하여 직접 설문으로 시행한다. 평가시점 +-1달 이내에 환자와 보호자가 외래 또는 입원 일정 부재로 병원에 내원하지 않는 경우에는 연구간호사가 환자와 보호자에게 전화를 연결하거나 우편/이메일을 발송하여 설문을 시행한다. 중재군 환자에 대해서는 연구간호사가 환자의 집에 방문하여 설문을 시행하는 것도 가능하다.
- 설문을 이메일로 시행하는 경우 각 시점과 대상자에 따라 링크를 첨부하여 발송한다.
  - 환자용 3개월 https://forms.gle/S8a96zBnZZRVJZj98
  - 환자용 6개월 https://forms.gle/imX9U8cBTGPg49YB9
  - 보호자용 3개월 https://forms.gle/11y2QwqejZ1jM7388
  - 보호자용 6개월 https://forms.gle/JL1SznfcR137Dfdr7
- 양 군의 환자와 보호자에게 시행하는 설문 평가와 연구자 면접 통한 조사 항목은 아래와 같다.
- 대조군에서는 3개월 설문 평가시 6개월 시점에서의 간접의료비용(돌봄비용) 설문평가 동의여부를 구두로 확인하여 6개월 설문평가에 동의한 환자에 대해서만 중재 6개월(+-1개월 가능) 시점에 간접의료비용에 대한 추가 설문을 수행한다.

Item	등록	3m	6m	12m	조사 방법
인구사회학적 특성<br>- 환자: 연령, 성별, 거주지, 의료보장, 학력, 결혼, 종교, 근로 상태, 월소득, 거주지, 복지서비스 및 장애 종류, 민간보험, 가구 특징, 가족 및 가구 구성, 돌봄 제공 현황<br>- 보호자: 연령, 성별, 환자와의 관계, 환자 동거 여부, 학력, 결혼, 종교, 근로 상태, 환자 돌봄 경력	◉				연구자 면접<br>연구 대상자 설문
환자 전신 상태	◉	◉	◉	◉	연구자 면접
보유 의료기기	◉				연구자 면접
증상 및 식이섭취량	●				
약물 평가	●				
환자 삶의 질 (EQ-5D)	◉	◉	●		연구 대상자 설문
환자 증상 (ESAS)	◉	◉	●		연구 대상자 설문
환자 정서 (PHQ-9)	◉	◉	●		연구 대상자 설문
환자 서비스 만족도		●	●		연구 대상자 설문
보호자 삶의 질 (EQ-5D)	◉	◉	●		연구 대상자 설문
보호자 정서 (PHQ-9)	◉	◉	●		연구 대상자 설문
보호자 돌봄 부담 (CRA-K)	◉	◉	●		연구 대상자 설문
보호자 돌봄 준비	⊙	⊙	●		
보호자 돌봄제공자 역량	⊙	⊙	●		
보호자 돌봄 보람	⊙	⊙	●		
보호자 서비스 만족도		●	●		연구 대상자 설문
보호자 간접의료비용		◉	◉*		연구 대상자 설문

◉ 중재군/대조군 모두, ● 중재군만

*대조군에서는 3개월 시점에서 6개월 설문에 동의한 경우에만 시행

나. 의무기록 조사

- 연구간호사는 환자 등록 시점에 해당 의료기관의 의무기록 조사를 통하여 과거력 (당뇨, 고혈압, 고지혈증, 심장질환, 뇌졸중/뇌출혈, 만성간질환, 만성 신장질환, 천식/만성폐쇄성 폐질환, 근골격계 질환, 소화기질환, 신경계질환, 정신과질환, 기타), 암 진단 (암종 ICD-10 코드 및 진단일), 암 치료력, 환자 연명의료계획 상태를 조사한다.
- 연구간호사는 환자 등록 3, 6, 12개월 시점에 해당 의료기관의 의무기록조사를 시행하여 이전 평가 시점으로부터 각 시점까지의 기간 동안의 암 치료 상태, 입원, 응급실 이용, 중증 의료이용 정보를 조사하고, 사망환자의 경우 사망 1달 전 의료이용 정보를 조사한다. 의무기록 조사를 통해 조사하는 항목은 다음과 같다.

	3개월	6개월	12개월
암 치료 상태<br>- 암 진행 상태<br>- 항암치료 계획<br>- 이외 치료 계획	O	O	O
의료기관 입원<br>- 입원일, 퇴원일<br>- 입원 경로 (외래, 응급실)<br>- 예정된 입원 여부<br>- 입원 결과 (귀가, 전원, 사망)	O	O	O
응급실 이용<br>- 이용일 (년월일)<br>- 응급실 방문시 KTAS<br>- 응급실 방문 주소<br>- 이용결과 (귀가, 입원, 전원, 사망)	O	O	O
중증 의료이용<br>- 중환자실 입원일 (day)<br>- 인공호흡기 적용일 (day)<br>- 심폐소생술 횟수	O	O	O
원내 사망여부<br>- 사망일<br>- 사망장소 (병동, 중환자실, 응급실)<br>- 사망원인 (사망진단서 기재 기준)	O	O	O
사망 1달 전 의료이용<br>- 중환자실 입원일 (day)<br>- 인공호흡기 적용일 (day)<br>- 심폐소생술 횟수<br>- 항암치료 여부	O	O	O

다. 2차 자료 평가

- 2차 자료 연계에 동의한 환자들에 대하여, 중재연구에 등록한 모든 자료연계 동의 환자가 등록 12개월 이상 경과한 시점에 건강보험공단 자료 또는 건강보험심사평가원 자료를 연계한다. 해당 자료원을 활용하여 등록 후 12개월 시점까지의 의료이용 정보와 직접 의료비용 정보를 조사한다. 12개월 시점에서 자료를 조사하여 3, 6, 12개월 시점에서의 의료이용 및 비용 정보를 재산출하여 활용한다.
- 중재연구에 등록한 모든 자료연계 동의 환자가 등록 18개월 이상 경과한 시점에서 18개월 시점에서의 사망 여부와 호스피스 이용, 사망 전 의료이용에 대한 정보를 조사한다.
- 건강보험공단 또는 건강보험심사평가원 자료와의 연계를 통해 조사하는 항목은 다음과 같다.

	12개월	18개월
의료기관 입원<br>- 입원일, 퇴원일<br>- 입원 경로 (외래, 응급실)<br>- 입원 결과 (귀가, 사망)	O	
응급실 이용<br>- 이용일 (년월일)<br>- 이용결과 (귀가, 입원, 사망)	O	
중증 의료이용<br>- 중환자실 입원일 (day)<br>- 인공호흡기 적용일 (day)<br>- 심폐소생술 횟수	O	O
호스피스 이용<br>- 입원일, 퇴원일	O	O
사망<br>- 사망여부<br>- 사망일 (자격상실일)	O	O
사망 1달 전 의료이용<br>- 중환자실 입원일 (day)<br>- 인공호흡기 적용일 (day)<br>- 심폐소생술 횟수	O	O
직접 의료비용 (원)	O	

- 2차 자료 연계에 동의한 환자들에 대하여, 중재연구에 등록한 모든 자료연계 동의 환자가 등록 12개월 이상 경과한 시점에 국가생명윤리정책원 자료를 연계한다. 해당 자료원을 활용하여 등록 후 12개월, 18개월 시점에서의 연명의료서식 작성 여부 정보를 조사한다.
- 국가생명윤리정책원 자료와의 연계를 통해 조사하는 항목은 다음과 같다.

	12개월	18개월
사전연명의료의향서 작성 여부, 작성일	O	O
연명의료계획서 작성 여부, 작성일, 작성자	O	O
임종과정 이행서 작성 여부, 작성일, 종류	O	O

12. 효과 평가 기준, 방법 및 해석

가. 평가 기준

1) 1차 평가 변수

- 연구 등록 후 6개월간 계획되지 않은 입원을 한 환자의 분율
- 계획되지 않은 입원의 정의: 해당 기관에 급성기 치료 목적으로 입원한 경우 (항암치료를 위한 예정된 입원, 수술/시술을 위해 입원일자를 지정하여 지정한 입원 제외), 해당 의료기관이 아닌 타 의료기관에 입원한 경우, **요양병원에 4주 이하 입원한 경우**

2) 2차 평가 변수

- 도메인1: 환자의 의료이용과 중증 의료이용에 관련된 2차 평가 변수는 다음과 같다.
  - 연구 등록 후 6개월간 계획되지 않은 입원 횟수 (연속형 변수 혹은 count 변수)
  - 환자의 입원일 (총 입원기간, 연속형 변수)
  - 환자의 입원여부 (이분형 변수)
  - 환자의 응급실 이용 횟수 (연속형 변수)
  - 환자의 중환자실 입원일 (총 입원 기간, 연속형 변수)

- 도메인2: 환자의 삶의 질과 서비스 이용 만족도 등과 관련된 2차 평가 변수는 다음과 같다.
  - 환자의 삶의 질 (EQ-5D, 연속형 변수)
  - 환자의 증상 조절 정도 (ESAS, 연속형 변수)
  - 환자의 정서 수준 (PHQ-9, 연속형 변수)
  - 환자의 서비스 만족도 (연속형 변수)

- 도메인3: 환자의 사망과 사전연명의료계획 수립과 관련된 2차 평가 변수는 다음과 같다.
  - 등록 후 생존기간 (일, 연속형 변수)
  - 환자의 사망 1개월 전 중환자실 입원일 (총 입원일수, 연속형 변수)
  - 환자의 사망 1개월 전 항암치료 여부 (이분형 변수)
  - 환자의 사망 1개월 전 인공호흡기 적용일 (총 적용 일, 연속형 변수)
  - 환자의 사전연명의료의향서 또는 연명의료계획서 작성 여부 (이분형 변수)

- 도메인4: 보호자의 삶의 질과 서비스 이용 만족도 등과 관련된 2차 평가 변수는 다음과 같다.
  - 보호자의 삶의 질 (EQ-5D, 연속형 변수)
  - 보호자의 정서 수준 (PHQ-9, 연속형 변수)
  - 보호자의 돌봄 부담 (CRA-K, 연속형 변수)

- 보호자의 돌봄 역량 (연속형 변수)
- 보호자의 서비스 만족도 (연속형 변수)

- 도메인5: 의료비용과 관련된 2차 평가 변수는 다음과 같다.
  - 직접 의료비용 (연속형 변수, 원)
  - 간접 의료비용 (연속형 변수, 원)

나. 평가 방법

1) 1차 평가 변수

- 중재군과 대조군에서 계획되지 않은 입원을 한 분율의 평균치를 비교한다.

2) 2차 평가 변수

- 연속형 변수로 평가된 도메인 1-5의 각 항목에 대해 중재군과 대조군에서 연속형 변수의 평균과 이분형 변수의 분율을 비교한다.
- 등록 후 생존기간은 중재군과 대조군의 Kaplan-Meier curve를 그려 비교한다.

다. 평가 해석

1) 1차 평가 변수

- 중재군의 연속형 변수의 평균이 대조군 연속형 변수의 평균보다 20% 이상 적을 때 재택의료 중재가 재택의료 제공 6개월 이내 계획되지 않은 입원을 줄이는 효과가 있다고 해석한다.

2) 2차 평가 변수

- 중재군의 연속형 변수의 평균과 대조군 연속형 변수의 평균 차이가 p value $<$ 0.05 일 때 재택의료가 해당 2차 평가 변수에 있어 효과적이라고 해석한다.

13. 임상시험 중지 및 대상자 중도 탈락 절차

가. 임상시험 중지기준

- 이하의 경우 임상시험을 중단한다.

1. 환자 또는 보호자가 재택의료 중재 중단을 원하는 경우
2. 연구 대상자가 사망하는 경우
3. 의료기관에 4주 초과로 입원하는 경우
4. 해당병원 혈액종양내과 진료를 종료하였거나 추적관찰이 중단된 경우
5. 입원형 호스피스 혹은 가정형 호스피스를 이용하는 경우
6. 연구자 판단에 따라 임상시험 참여 중단이 대상자에게 최선인 경우
7. 연구 대상자가 임상시험 담당의사에게 협조하지 않거나 임상시험 담당의사의 지침을 따르지 않는 경우
8. 규제당국 또는 윤리위원회/임상연구심사위원회가 본 시험을 중단시키는 경우

나. 임상시험 중지절차

- 재택의료 중재군의 경우 중재 중단이 결정되면 다시 중재를 재개할 수 없다
- 임상시험 중단이 결정되면 사망에 의해 중지한 경우를 제외한 연구 대상자 (환자 및 보호자)에게 추가 설문 진행에 대한 동의 여부를 확인한다.

다. 임상시험 중지 대상자의 추적 관찰

- 임상시험 중지한 연구 대상자에 대해 중지 시점부터 남은 예정된 추적 관찰 기간 (등록 후 18개월) 동안 대상자의 사망, 추적관찰 실패, 혹은 동의 철회 시까지 의무기록 조사, 2차 자료 연계를 시행할 것이다. 연구 종료 후 본 임상시험 외의 추가적인 재택의료 서비스(다른 재택의료 시범사업 등)를 제공받은 경우 증례기록서에 기록한다.
- 사망이 아닌 사유로 임상시험 중지한 연구 대상자에서 추적 설문 평가의 경우 동의하는 자를 대상으로만 시행하며, 동의할 경우 연구 간호사가 전화 조사를 통하여 시행한다.

라. 임상시험 대상자 중도 탈락 절차

- 임상시험 대상자는 언제든지 자유롭게 임상시험 참여(재택의료 중재 제공과 평가 절차)를 중단할 수 있다 (동의 철회). 이러한 시험 대상자에게는 항상 그 사유를 확인할 것이다. 가능하다면 시험자가 시험대상자 면담을 시행하고 예정된 시험 종료 후 평가와 절차를 시행할 것이다.
- 동의 철회하는 중도 탈락자에게는 기존 자료의 연구 활용 가능 여부를 확인하여 동의하지 않는 경우 기존에 수집된 자료를 폐기한다.

14. 통계적 측면

가. 연구가설

- 재택의료 중재군과 비교군은 의료이용과 삶의 질에 있어서 차이가 있다.
- 연구의 일차가설: 재택의료 중재군은 비교군에 비하여 계획되지 않은 입원의 한 분율이 평균 20% 작다.

나. 결과변수

- 일차결과변수: 6개월 이내의 계획되지 않은 입원을 한 환자의 분율
- 이차결과변수: 의료이용과 중증 의료이용 (연구 등록 후 6개월간 계획되지 않은 입원여부, 환자의 입원일, 환자의 입원여부, 환자의 응급실 이용 횟수, 환자의 중환자실 입원기간), 사망과 사전연명의료계획 수립 (등록 후 생존기간, 사망 1개월 전 중환자실 입원일, 사망 1개월 전 항암치료 여부, 사전연명의료의향서 또는 연명의료계획서 작성 여부), 환자의 삶의 질과 서비스 이용 만족도 (삶의 질, 증상 조절 정도, 정서 수준, 서비스 만족도), 보호자의 삶의 질과 서비스 이용 만족도 (삶의 질, 정서 수준, 돌봄 부담, 돌봄 역량, 서비스 만족도), 의료비용 (직접 의료비용, 간접 의료비용)

다. 분석집단

- 재택의료 중재군과 비교군
- 각 참여 의료기관

라. 통계분석 방법

- 재택의료 중재군 정의 및 중도 탈락: 재택의료 중재 기관을 선정하여 해당 기관의 시험동의를 받은 환자에 대해 중재군으로 분류하며, 재택의로 비교군 기관의 시험동의를 받은 환자에 대해 비교군으로 정의함. 연구대상자의 분석군 분류의 경우 최초 분류된 중재 여부에만 의존하는 ITT(intention-to-treat)에 의한 군 분류를 원칙으로 하여 중도에 재택의료 서비스가 누락되는 경우에도 재택의료 중재군으로 정의되기 때문에 치료군의 효과를 감소 시키는 편향이 발생함. 즉 치료군의 효과를 보수적으로 추정할수 있도록 설계함에도 불구하고 통계적으로 유의한 차이를 확인하여 연구 결과를 도출하며, 추적관찰 중 동의철회 또는 중도 탈락이 발생하는 경우 동의 철회 또는 중도 탈락 시점까지의 자료를 활용하고 군별 탈락률에 대한 비교 분석 및 보고함
- 기저특성에 대한 기술통계
  연속형 변수: 평균 (표준편차), 중앙값 (IQR)
  이분형 변수: 빈도 (%)
- 군간 차이에 대한 분석
  - 이분형 변수(1차 평가 변수): 참여 기관 내 환자의 cluster를 고려하기 위하여 GEE를 사용하며, 로짓 연결함수(logit link function)를 매개로 하여 로지스틱 회귀모형을 기본모형으로 재택의료 중재의 효과를 평가함
  - 연속변수: 참여기관 내의 환자의 cluster를 고려하기 위하여 Mixed model을 이용하여 재택의료 중재의 효과를 평가함.
  - Count data: 참여기관 내의 환자의cluster를 고려하기 위하여 GEE를 사용하며, link function으로 로그 링크 함수(log link function)를 매개로 하여 변수의 분포에 따라 Poisson, negative binomial을 랜덤성분으로 적절히 사용한 회귀 모형을 통해 재택의료 중재의 효과를 평가함.
  - 재택의료 두 군의 비교성확보를 위해 일차적으로 ITT에 의한 군분류를 원칙으로 하여 보수적으로 추정된 통계적 유의성을 확인하며, 연구대상자의 기저특성을 교란요인으로 고려하여 이분형 변수, 연속변수, Count data분석에서 공변량으로 활용하고자 함

15. 안전성 평가기준, 평가방법 및 보고방법

- 본 임상시험의 중재군으로 배정되는 연구 대상자는 교육과 상담 서비스로 이루어지는 재택의료 프로그램을 제공받는 것이므로 중재에 따른 부작용이나 위험은 없을 것으로 예상됨. 대조군으로 배정되는 연구대상자는 현재 통상적으로 제공되는 기존의 의료서비스를 동일하게 받을 것이므로 연구 참여로 인한 부작용이나 위험이 없음.

16. 피해자 보상에 대한 규약 및 피험자 안전보호에 관한 대책

가. 윤리적 측면

1) 연구심의 위원회

- 시험자는 시험 시작 전에 임상시험계획서, 시험대상자 설명문 및 동의서 양식, 시험대상자 모집 자료/절차(예: 광고) 및 시험대상자에게 제공하는 모든 서면 정보에 대해서 IRB로부터 서면으로 된 승인서를 득해야 한다. 시험자는 임상시험 실시기관 절차에 따라 IRB에 보

고해야 할 업데이트 및 기타 정보들(예: 신속 안전성 보고, 개정, 및 행정 서신 등)을 제공해야 한다.

2) 시험의 윤리적 수행

- 본 임상시험은 헬싱키선언, 국내법 및 관련 규정을 준수하여 실시된다. 본 임상시험은 임상시험계획서를 준수하여 수행되고 임상시험계획서/임상시험계획서 개정 및 시험대상자 동의서는 시험 시작 전에 임상시험 심사위원회(IRB; Institutional Review Board)의 승인을 받아야 한다.
- 본 임상시험의 수행에 관여하는 시험자는 본인의 업무 수행을 위한 교육, 훈련 및 경험을 통하여 적절한 자격을 갖추어야 한다.
- 본 임상시험에는 제제 조치를 당하거나 과학적 부정 행위 또는 기만과 연관된 (예, 의사면허 상실, 제제) 시험자는 참여할 수 없다.
- 본 시험의 모든 측면에서 품질을 보장하기 위한 절차 및 시스템이 실행될 것이다.

3) 시험대상자 정보 및 동의

- 본 임상시험과 관련된 절차가 수행되기 전에 모든 시험대상자로부터 동의서를 받을 것이며, 시험대상자 설명문 및 동의서는 IRB의 승인을 받을 것이다.
- 시험책임자 (혹은 위임자)는 시험대상자에게 본 임상시험에 대해 충분히 설명한다. 시험대상자는 자발적인 의사에 따라 동의하였고 서명된 동의서 사본을 제공받는다. 시험대상자는 임상시험 중에 어떠한 사유가 되었건, 언제든지 동의철회 할 수 있다.
- 임상시험 참여에 대한 동의서와 별도로, 본 연구 종료 후 본 연구 목적 이외의 2차 연궁에 사용해도 될지에 대한 제3자 제공 및 2차 연구 이용 동의서는 별도 파일로 두어, 동의하는 자는 별도로 동의서를 작성한다.

4) 피해에 대한 보상

- 본 임상시험에서 중재군에서는 교육과 상담으로 이루어진 중재를 받게 되고, 대조군에서는 통상적 의료 서비스를 이용하므로, 이 연구로 인해 연구대상자가 추가적으로 입게 되는 신체적, 정신적 위해 및 특이 손상은 없을 것으로 예측된다.
- 임상시험 도중, 기존 진료 과정 외 임상시험 참여로 추가된 절차 및 개입으로 인해, 예측되지 못한 심리적, 정신적 피해 등이 발생할 경우, 연구대상자가 적절한 처치를 받을 수 있도록 가능한 최선의 조치를 취할 것이며, 임상시험과 손상 사이의 합리적인 인과관계가 있는 경우 시험대상자에게 보상한다.

나. 실천상의 측면

1) 임상시험계획서 및 임상시험계획서 변경의 준수

- 본 임상시험은 승인된 임상시험계획서에 명시되어 있는 대로 시행될 것이다. 시험자는 변경에 대한 IRB의 사전 검토와 서면 승인서 없이 임상시험계획서를 미준수(deviation)하거나 변경해서는 안 된다. 단, 시험대상자에게 직접적인 위험을 제거하기 위해 필요한 경우는 예외이다.

2) 기록 보관

- 시험자는 해당 법규와 지침 또는 의뢰자가 명시한 기간에 맞게 재택의료 프로그램의 제공 기록, 증례기록서 사본(또는 전자 파일) 및 근거 문서를 보관해야 한다.

3) 증례기록서

- 시험자는 재택의료 중재 프로그램을 제공하며 획득한 모든 관찰 및 연구와 관련된 기타 자료를 기록하기 위해 고안된 적절하고 정확한 증례 기록 이력을 마련하고 보관해야 한다. 증례기록서 상 보고되는 자료들은 근거 문서들을 기반으로 하는 것이기 때문에, 근거 문서와 일치해야 하며 불일치한 점은 설명되어야 한다.
- 시험대상자를 식별할 수 있는 기록의 비밀 보장은 해당 규제 요건을 준수하여 개인 정보 및 기밀 유지를 존중하며 보호되어야 한다.
- 시험자는 증례기록서에 입력을 하거나 그리고/또는 수정을 하도록 승인을 받은 모든 사람의 서명 및 이니셜을 기록하기 위한 서명 문서를 유지할 것이다.
- 시험자는 변경 및 수정에 대한 기록을 포함하여 증례기록서 사본을 유지해야 한다.

17. 참고문헌

Brumley RD, Enguidanos S, Cherin DA. Effectiveness of a home-based palliative care program for end-of-life. J Palliat Med. 2003 Oct;6(5):715-24. doi: 10.1089/109662103322515220. PMID: 14622451.
Brumley R, Enguidanos S, Jamison P, Seitz R, Morgenstern N, Saito S, McIlwane J, Hillary K, Gonzalez J. Increased satisfaction with care and lower costs: results of a randomized trial of in-home palliative care. J Am Geriatr Soc. 2007 Jul;55(7):993-1000. doi: 10.1111/j.1532-5415.2007.01234.x. PMID: 17608870.
Chen CY, Thorsteinsdottir B, Cha SS, Hanson GJ, Peterson SM, Rahman PA, Naessens JM, Takahashi PY. Health care outcomes and advance care planning in older adults who receive home-based palliative care: a pilot cohort study. J Palliat Med. 2015 Jan;18(1):38-44. doi: 10.1089/jpm.2014.0150. PMID: 25375663; PMCID: PMC4273188.
Brian Cassel J, Kerr KM, McClish DK, Skoro N, Johnson S, Wanke C, Hoefer D. Effect of a Home-Based Palliative Care Program on Healthcare Use and Costs. J Am Geriatr Soc. 2016 Nov;64(11):2288-2295. doi: 10.1111/jgs.14354. Epub 2016 Sep 2. PMID: 27590922; PMCID: PMC5118096.
Lustbader D, Mudra M, Romano C, Lukoski E, Chang A, Mittelberger J, Scherr T, Cooper D. The Impact of a Home-Based Palliative Care Program in an Accountable Care Organization. J Palliat Med. 2017 Jan;20(1):23-28. doi: 10.1089/jpm.2016.0265. Epub 2016 Aug 30. PMID: 27574868; PMCID: PMC5178024.
Pouliot K, Weisse CS, Pratt DS, DiSorbo P. First-Year Analysis of a New, Home-Based Palliative Care Program Offered Jointly by a Community Hospital and Local Visiting Nurse Service. Am J Hosp Palliat Care. 2017 Mar;34(2):166-172. doi: 10.1177/1049909115617139. Epub 2016 Jul 11. PMID: 26656032.
Dhollander N, Smets T, De Vleminck A, Van Belle S, Deliens L, Pardon K. Phase 0-1 early palliative home care cancer treatment intervention study. BMJ Support Palliat Care. 2019 May 8: PMID: 31068333.
Rich, M. W., Beckham, V., Wittenberg, C., Leven, C. L., Freedland, K. E., & Carney, R. M. (1995). A multidisciplinary intervention to prevent the readmission of elderly patients with congestive heart failure. New England Journal of Medicine, 333(18), 1190-1195.
Stewart, S., Pearson, S., & Horowitz, J. D. (1998). Effects of a home-based intervention among patients with congestive heart failure discharged from acute hospital care. Archives of Internal Medicine, 158(10), 1067-1072.
Stewart, S., Vandenbroek, A. J., Pearson, S., & Horowitz, J. D. (1999). Prolonged beneficial effects of a home-based intervention on unplanned readmissions and mortality among patients with congestive heart failure. Archives of internal medicine, 159(3), 257-261.
Stewart, S., Marley, J. E., & Horowitz, J. D. (1999). Effects of a multidisciplinary, home-based intervention on planned readmissions and survival among patients with chronic congestive heart failure: a randomised controlled study. The Lancet, 354(9184), 1077-1083.
Stewart, S., Wiley, J. F., Ball, J., Chan, Y. K., Ahamed, Y., Thompson, D. R., & Carrington,

M. J. (2016). Impact of nurse-led, multidisciplinary home-based intervention on event-free survival across the spectrum of chronic heart disease: composite analysis of health outcomes in 1226 patients from 3 randomized trials. Circulation, 133(19), 1867-1877.
Carrington, M. J., Chan, Y. K., Calderone, A., Scuffham, P. A., Esterman, A., Goldstein, S., & Stewart, S. (2013). A multicenter, randomized trial of a nurse-led, home-based intervention for optimal secondary cardiac prevention suggests some benefits for men but not for women: the Young at Heart study. Circulation: Cardiovascular Quality and Outcomes, 6(4), 379-389.
Hermiz, O., Comino, E., Marks, G., Daffurn, K., Wilson, S., & Harris, M. (2002). Randomised controlled trial of home based care of patients with chronic obstructive pulmonary disease. Bmj, 325(7370), 938.
Stewart, S., Pearson, S., Luke, C. G., & Horowitz, J. D. (1998). Effects of home‐based intervention on unplanned readmissions and out‐of‐hospital deaths. Journal of the American Geriatrics Society, 46(2), 174-180.
Hughes, S. L., Weaver, F. M., Giobbie-Hurder, A., Manheim, L., Henderson, W., Kubal, J. D., ... & Department of Veterans Affairs Cooperative Study Group on Home-Based Primary Care. (2000). Effectiveness of team-managed home-based primary care: a randomized multicenter trial. Jama, 284(22), 2877-2885.
Cordesse, V., Sidorok, F., Schimmel, P., Holstein, J., & Meininger, V. (2015). Coordinated care affects hospitalization and prognosis in amyotrophic lateral sclerosis: a cohort study. BMC health services research, 15(1), 1-6.
Pozzilli, C., Brunetti, M., Amicosante, A. M. V., Gasperini, C., Ristori, G., Palmisano, L., & Battaglia, M. (2002). Home based management in multiple sclerosis: results of a randomised controlled trial. Journal of Neurology, Neurosurgery & Psychiatry, 73(3), 250-255.
Pouliot, K., Weisse, C. S., Pratt, D. S., & DiSorbo, P. (2017). First-year analysis of a new, home-based palliative care program offered jointly by a community hospital and local visiting nurse service. American Journal of Hospice and Palliative Medicine®, 34(2), 166-172.
Lustbader, D., Mudra, M., Romano, C., Lukoski, E., Chang, A., Mittelberger, J., ... & Cooper, D. (2017). The impact of a home-based palliative care program in an accountable care organization. Journal of palliative medicine, 20(1), 23-28.
Brian Cassel, J., Kerr, K. M., McClish, D. K., Skoro, N., Johnson, S., Wanke, C., & Hoefer, D. (2016). Effect of a home‐based palliative care program on healthcare use and costs. Journal of the American Geriatrics Society, 64(11), 2288-2295.
