# Supplementary material for: SupporTive Care At Home Research (STAHR) for patients with advanced cancer: Protocol for a cluster non-randomized controlled trial
Source: PLoS One. 2024 May 13;19(5):e0302011. doi: 10.1371/journal.pone.0302011 (PMC11090303; doi:10.1371/journal.pone.0302011)
Supplement: S2 File — (PDF) [file pone.0302011.s004.pdf]

Study Protocol

A Cluster Non-randomized Controlled Trial on the Effectiveness of a Korean Model for Home-based Care among Patients with Advanced Cancer

Principal Investigator

Belong Cho

Public Healthcare Center, Seoul National University Hospital

Clinical Trial Protocol Overview

Korean Title	Research-led, Non-randomized Cluster Clinical Trial on the Effectiveness of Home-Based Medical Care in Reducing Unscheduled Hospitalizations Within 6 Months of Enrollment Among Advanced Solid Cancer Patients Undergoing Anti-cancer Treatments
English Title	A Cluster Non-randomized Controlled Trial on the Effectiveness of a Korean Model for Home-Based Care Among Patients with Advanced Cancer
Principal Investigator	Professor Belong Cho, Family Medicine and Comprehensive Community Care Center, Seoul National University Hospital
Study Purpose	To determine the effect of home care education for advanced cancer patients with mobility difficulties and their family caregivers using a home-based medical care (HBMC) intervention program in which home visits and periodic condition checks are provided by the medical staff to reduce unscheduled hospitalizations within six months.
Study Design	Korean multicenter non-randomized cluster clinical trial
Study Period	Date of IRB approval – December 31, 2026
Hypotheses	1. Use of medical services, such as unscheduled hospitalizations and emergency room (ER) visits, will be significantly reduced among advanced cancer patients who receive home care education and participate in the HBMC intervention program as compared to the patients in the control group.<br>2. The quality of life (QoL) will be significantly higher, and the degree of symptom control will be significantly better among advanced cancer patients who receive home care education and participate in the HBMC intervention program than those in the control group.<br>3. The rate of establishing advance care planning will be significantly higher among advanced cancer patients who receive home care education and participate in the HBMC intervention program than those in the control group.<br>4. The mortality rate will be significantly lower among advanced cancer patients who receive home care education and participate in the HBMC intervention program than those in the control group.
Basis of the Study	- The provision of home care environment-based intervention to patients with severe illnesses can significantly reduce readmission rates and improve survival rates compared to the provision of existing hospitalization and outpatient services (Malik et al., 2019).<br>- Symptom control is better when home-based palliative care services are provided to cancer patients (Gomes et al., 2013).
Participants	Advanced solid cancer patients with mobility difficulties undergoing cancer treatment and their family caregivers
Number of Study Participants (based on the number of patients)	- Total number of participants (6 institutions): 396; Participants at the intervention institution: 66<br>- Three institutions will be assigned to the intervention and control groups each. A total of 396 patients will be enrolled. The ratio of participants between the intervention and control groups will be 1:1 with 198 patients enrolled in each group.<br>- The rationale for sample size calculation: The number of samples in the home care management program in this study is based on the binary variable, the primary outcome indicator, to secure an appropriate number of participants for stable and sufficient power. The primary endpoint of this clinical trial is

	unscheduled hospitalization within 6 months of enrollment in the study. The study aims to reveal the difference in the effectiveness of the HBMC program by examining the odds ratio based on the proportion of unscheduled hospitalizations in the intervention and control groups. Although no previous study using an intervention with a similar design has been conducted overseas, the difference between the intervention and control groups can be expected to be approximately 20% (20% lower in the HBMC group) based on the literature review. The number of institutions in each group will be 3. The parameters will be estimated using generalized estimating equations (GEE) to consider the cluster effect of the participants at each institution. The two groups will be compared using a logistic regression model, a model of comparison of the dichotomous outcome variable. Considering the above analysis method and the assumptions of the power of 80%, a significance level of 0.05 to control type I error, a 20% change in the number of unscheduled hospitalizations before and after the intervention (20% lower in the HBMC group), and 1:1 ratio of participants between the intervention and control groups, the minimum sample size should be 198 in the intervention group and 198 in the control group, totaling 396 participants (assuming within-cluster coefficient of 0.015). Assuming a maximum dropout rate of 15%, the final number of participants to be recruited is approximately 57 for each cluster, and a total of 342 participants are expected to be recruited. The expected minimum power is 78.8% (PASS 2022, v.22.0.2).<br>- Three institutions (i.e., Seoul National University Hospital, Chung-Ang University Hospital, and Dongguk University Ilsan Hospital) will participate as the intervention institutions, and three institutions (i.e., Kyung Hee University Hospital, Cha University Bundang Medical Center, and Seoul National University Bundang Hospital) will participate as the control institutions.
Vulnerable Study Participants	Not applicable

Procedures	**1. Participant Recruitment**<br>- Post the essential information in the outpatient and inpatient wards and public bulletin boards of the participating institutions. Explain the study to the patients and their guardians who meet the inclusion criteria in the outpatient or inpatient ward of co-researchers and register consenting individuals.<br><br>**2. Clinical Trial**<br>1) The Intervention Group (HBMC provided)<br>- The HBMC teams of institutions that will provide the intervention will include physicians, nurses, and social workers. Physicians and social workers involved in the existing patients' treatment and consultation can concurrently serve on the HBMC team, and nurses will be dedicated to home care.<br>- For inpatients, a HBMC team nurse will conduct the initial assessment, provide home care educational materials, and conduct home care education within a week before the scheduled discharge date. In the case of outpatients, the initial evaluation, provision of home care educational materials, and home care education will be conducted by a HBMC team nurse on the day of the outpatient visit.<br>- Within two weeks of enrollment in the study, a HBMC team medical staff (e.g., physicians and nurses) will visit the patient's home to assess their home care environment and educate them on the requirements for an appropriate home-care environment. The home care education will include education on taking medications, possible symptoms, and establishing care goals.<br>- The HBMC team will conduct monthly multidisciplinary meetings to share information on patients in the HBMC group and modify the home care management plan through periodic reevaluations.<br>- After the first home visit, the HBMC team nurse will check the patient's home care management status by contacting the patient and their guardian via phone call or text message every two weeks. The HBMC team physicians will be notified about the changes in patients' symptoms, and the medications, outpatient visits, and hospitalization schedules will be modified accordingly.<br>- Patients receiving the HBMC will be provided with a phone number reachable between 09:00 AM and 05:00 PM. This number will be operated by a nurse on the HBMC team who will provide the required consultation and education.<br>- Patients would be allowed to use other medical services, such as existing hospitalization, outpatient visit, ER visit, home care, and visit to a medical institution in the community as before.<br>2) The Control Group<br>- A research nurse will provide home care educational materials to patients who have consented to participate in the study one week before their discharge in the case of inpatients and on the day of the outpatient visit for outpatients.<br>- The patients can continue using the existing inpatient, outpatient, ER, home care, and community medical institution services other than the HBMC.<br><br>3) Analysis Methods<br>- Patients in the intervention and control groups will be followed up, after which a comparative analysis of the outcome variables will be performed.
Suspension Criteria	1. When a patient or their caregiver wishes to discontinue the HBMC intervention<br>2. When a participant passes away<br>3. When a participant is hospitalized at a medical institution for an additional four weeks<br>4. When a participant's treatment at the haemato-oncology department of the respective hospital has been terminated or the follow-up has been

	discontinued<br>5. When a participant uses an inpatient or home-based hospice care services<br>6. When the suspension of participation in the clinical trial is deemed best for the participant at the investigator's discretion<br>7. When a participant does not cooperate with the physician in charge of the clinical trial or does not follow the physician's instructions<br>8. When the regulatory authority, ethics committee, or Institutional Review Board suspends the clinical trial<br><br>* The termination of the study for patients who discontinue the study is as follows.<br>- Reasons 1, 3, 4, 5, 6, 7, or 8: Continuation of the investigation of medical records and secondary linkage data (18 months) until 12 months after the enrollment or withdrawal of consent; the additional survey will be conducted only with the patient's consent<br>- Reason 2: Survey or investigation of medical records will not be possible until 12 months after the enrollment; secondary data linkage will be conducted<br><br>* When participation consent is withdrawn, the previously collected data of the patient will be discarded if the patient does not wish the data to be used in the study.
Inclusion Criteria	Both the patient and their guardian must meet the inclusion criteria to be eligible for participation in the study.<br>1. Patient<br>① A patient diagnosed with advanced-stage solid cancer (ICD-10 code C00-C70)<br>①-1. A patient undergoing or planning cancer treatment<br>② A patient who meets one of the following conditions:<br>②-1. A patient with Eastern Cooperative Oncology Group (ECOG) performance status score of 2<br>②-2. A patient with an ECOG performance status score of 1 and aged 70 years or above<br>③ A patient who wishes to stay at home<br>④ A patient whose family guardian resides at home<br>⑤ A patient who wishes to participate in the study<br>2. Guardian<br>① A family member of the patient<br>* Family: Patient's spouse (including de facto partners), lineal ascendant and descendant and their spouses, siblings and their spouses, and third cousins and their spouses.<br>② A patient who meets one of the following conditions:<br>②-1. An individual living with the patient (a member of the patient's household)<br>②-2. An individual who does not live with the patient but visits the patient's home at least three times a week<br>③ An individual who wishes for the patient to live at home<br>④ An individual who can communicate with medical staff fluently<br>⑤ An individual who wishes to participate in the study

Exclusion Criteria	If the exclusion criteria apply to either the patient or their guardian, both are ineligible for participation.<br>1. Patient<br>① An individual who cannot speak, understand, or read Korean<br>② An individual who is judged by a physician to have a deplorable medical condition and is unable to participate in this study<br>③ An individual whose residence is located outside the range of home visits (the distance is specified in advance by each medical institution) for the medical staff of the respective medical institution (※)<br>④ An individual who has used inpatient or home hospice-palliative care services<br>※ Not applicable for the exclusion criteria of the control group<br>⑤ An individual under 19 years of age<br>2. Guardian<br>① An individual who cannot speak, understand, or read Korean<br>② An individual who is judged by a physician to have a deplorable medical condition and is unable to participate in this study<br>③ An individual under 19 years of age
Efficacy Assessment	1. Data Collection<br>1) Questionnaire<br>- Patients and guardians in both the intervention and control groups will complete a questionnaire at the time of participant registration.<br>- Participants in the intervention and control groups will complete a follow-up questionnaire 3 months after registration.<br>- Participants in the intervention group will complete an additional questionnaire 6 months after registration. In the case of the control group, only those who will provide consent to participate in the additional questionnaire at 3 months will complete the additional questionnaire at 6 months.<br><br>2) Medical Records Investigation<br>- Medical records at medical institutions will be investigated at 3, 6, and 12 months after the patient's registration for hospitalization (number and dates of hospitalizations), critical care use (date of ICU admission, date of ventilator support, and number of CPR attempts), and emergency room use (number of emergency room visits) at each time point.<br>- In the case of death at 3, 6, and 12 months after registration, the deceased patient's use of medical services (hospitalization, critical care use, and emergency room use) within one month of their death will be investigated.<br><br>3) Secondary Data Linkage<br>- For patients who will provide consent to secondary data linkages, the hospitalizations, medical expenses, death, type of end-of-life medical services used, and the use of emergency room, critical care, and hospice between the time of registration and 12 months will be investigated when all patients have been enrolled for 12 months or longer.<br>- Death, information related to hospice use, and the type of end-of-life medical services used will be investigated when all patients have been enrolled for 18 months or longer.<br>- For patients who will consent to secondary data linkages, whether legal forms for life-sustaining treatments (e.g., advance directives and physician orders for life-sustaining treatment) have been completed and by whom will be investigated at 12 and 18 months of enrollment when all patients have been enrolled for 12 and 18 months or longer, respectively.

	2. Efficacy Endpoints<br>- Primary endpoint: The number of unscheduled hospitalizations within 6 months of study enrollment.<br>* Unscheduled hospitalization: This includes hospitalization at a respective participating institution for acute treatments (excluding scheduled hospitalizations for anti-cancer treatments, operations, and procedures), hospitalization at medical institutions other than the participating institutions, or hospitalization of 4 weeks or less at a nursing hospital.<br>- Secondary endpoint<br>n Patient: Medical service use (e.g., hospitalization and ER visit), critical care use (e.g., ICU admission and ventilator support), use of end-of-life medical services (e.g., ICU admission one month before death and ventilator support), satisfaction with the services, quality of life (ESAS and EQ5D), affective disorders (PHQ9), and preparation of advance directives.<br>n Guardian: Satisfaction with the services, quality of life (EQ5D), burden of care (CRA-K), affective disorders (PHQ9), and care competency.<br>n Costs: Medical expenses and care costs
Safety Assessment	Not applicable
Statistical Analysis	The HBMC intervention group, control group, and each participating institution will be set as analysis groups for the statistical analysis of the outcome variables.<br>The analysis groups of the participants are based on the group assignment according to the initial intention-to-treat (ITT) that relies solely on the intervention of the original assignment to estimate the effectiveness in the intervention group conservatively. In the case of withdrawal of consent or dropout during follow-up, data collected till the time of withdrawal or dropout will be used. A comparative analysis of the dropout rate between the two groups will be performed and reported.<br>- Descriptive analysis of underlying characteristics<br>Continuous variables: mean (standard deviation) and median (IQR)<br>Dichotomous variables: frequency (%)<br>- Analysis of between-group differences<br>An analysis will be performed using a regression model while considering that participants, the observation units, were clustered into six participating institutions to evaluate the effects of the HBMC intervention and correcting for the differences in the baseline characteristics between the groups.<br>Dichotomous variable (primary endpoint): A generalized estimation equation (GEE) will be used to analyze the clusters of patients in participating institutions, and the effects of the HBMC intervention will be evaluated using a logistic regression model based on the logit link function.<br>Continuous variables: The effects of the HBMC intervention will be evaluated using a mixed model to consider the patient clusters in the participating institutions.<br>Count data: A GEE will be used to consider the patient clusters in the participating institutions, and the effects of the HBMC intervention will be evaluated using a regression model that appropriately uses Poisson and negative binomial as random components according to the distribution of variables using the logit link function as the link function.
Expected Effects and Results	Create a basis for the dissemination of the HBMC services for severely ill patients in Korea by developing a suitable HBMC model for severely ill patients in Korea and evaluating its effectiveness.

Study Protocol

1. Study Title

(Korean) Research-led, Non-randomized Cluster Clinical Trial on the Effectiveness of Home-Based Medical Care in Reducing Unscheduled Hospitalizations Within 6 Months of Enrollment Among Advanced Solid Cancer Patients Undergoing Anti-cancer Treatments

(English) A Cluster Non-randomized Controlled Trial on the Effectiveness of a Korean Model for Home-Based Care Among Patients with Advanced Cancer

2. Names and Addresses of the Participating Institutions

Institution Name	Institution Address
Seoul National University Hospital	101, Daehak-ro, Jongno-gu, Seoul, 03080
Seoul National University Bundang Hospital	82, Gumi-ro 173beon-gil, Bundang-gu, Seongnam-si, Gyeonggi-do, 13620
Dongguk University Ilsan Hospital	27, Dongguk-ro, Ilsandong-gu, Goyang-si, Gyeonggi-do, 10326
Chung-Ang University Hospital	102, Heukseok-ro, Dongjak-gu, Seoul, 06973
Cha University Bundang Medical Center	59, Yatap-ro, Bundang-gu, Seongnam-si, Gyeonggi-do, 13496
Kyung Hee University Hospital	23, Kyungheedae-ro, Dongdaemun-gu, Seoul, 02453

3. Names and Titles of the Principal Investigator and Co-investigators

1) Principal investigator: Professor Belong Cho, Family Medicine and Comprehensive Community Care Center, Seoul National University Hospital

2) Internal co-investigators (in alphabetical order)

Name	Affiliation	Designation
Kyae Hyung Kim	Public Healthcare Center, Seoul National University Hospital	Clinical Associate Professor
Minsun Kim	Public Healthcare Center, Seoul National University Hospital	Clinical Assistant

		Professor
Sohee Kim	Public Healthcare Center, Seoul National University Hospital	Research Coordinator
Beomseok Kim	Hemato-oncology Department and Palliative Care and Clinical Ethics Center, Seoul National University Hospital	Clinical Professor
Yoonkyung Bae	Palliative Care and Clinical Ethics Center, Seoul National University Hospital	Researcher
Jeongmi Shin	Public Healthcare Center, Seoul National University Hospital	Clinical Assistant Professor
Shin Hye Yoo	Center for Palliative Care and Clinical Ethics, Seoul National University Hospital	Clinical Assistant Professor
Gayeong Lee	Public Healthcare Center, Seoul National University Hospital	Research Coordinator
Sun Young Lee	Public Healthcare Center, Seoul National University Hospital	Clinical Assistant Professor
Joongyub Lee	Preventive Medicine, Seoul National University College of Medicine	Professor
Minseol Jang	Public Healthcare Center, Seoul National University Hospital	Clinical Instructor
Hyeonjeong Han	Social Welfare Team, Seoul National University Hospital	Social Worker
In-Young Hwang	Public Healthcare Center, Seoul National University Hospital	Clinical Assistant professor

3) External co-investigators (in alphabetical order)

Name	Affiliation	Designation
Beodeul Kang	Department of Internal Medicine, Bundang CHA Hospital	Professor
Do yeon Kim	Department of Internal Medicine, Dongguk University Ilsan Hospital	Professor
Dalyong Kim	Hemato-oncology Department, Dongguk University Ilsan Hospital	Professor
Yu Jung Kim	Department of Internal Medicine, Seoul National University Bundang Hospital	Professor
Sun Kyung Baek	Department of Internal Medicine, Kyung Hee University Hospital	Professor
Jinah Shim	School of AI Convergence, Hallym University	Professor
Chungryeol Oh	Hemato-oncology Department, Chung-Ang University Hospital	Professor
In Gyu	Department of Internal Medicine, Chung-Ang University	Professor

Hwang	Hospital	

3) Research directors

■ Professor Sinhye Yoo, Palliative Care and Clinical Ethics Center, Seoul National University Hospital (ifi1024@gmail.com)

■ Professor Sunyoung Lee, Comprehensive Community Care Center, Seoul National University Hospital (sy2376@gmail.com)

4) Investigational drug managing pharmacist/investigational medical device manager: Not applicable

4. Sponsoring Institution: Not applicable

5. Name and Address of the Research Funding Institution:

1) Name: National Evidence-based Healthcare Collaborating Agency

2) Address: 7F, Namsan Square Building, 173, Toegye-ro, Jung-gu, Seoul

6. Estimated Study Period: IRB approval date – December 31, 2026

7. Disease Under Study: Patients with advanced cancer

1. The title of the clinical study

A Cluster Non-randomized Controlled Trial on the Effectiveness of a Korean Model for Home-Based Care Among Patients with Advanced Cancer

2. Introduction

A. Background

*** The unmet medical needs of patients with severe diseases**

Patients with advanced stage cancer prefer to spend their lives in their homes, accept the inevitable, and prepare for their death. Therefore, providing adequate medical services to meet patients' requirements are a worthwhile goal for the present medical system. Hospitalized patients are forced to give up their life values. However, they have the right to live on their own terms at their homes, receive proper treatment and care, and spend time as they wish. Thus, medical services must provide home-based medical care to fulfill the needs and wishes of patients with advanced cancer.

*** A lack of medical support for patients with advanced cancer**

The HBMC and supportive care for patients with advanced cancer who live with chronic conditions often have unmet medical needs. Patients with advanced cancer often find it expensive to undergo treatment in hospitals and they do not receive the HBMC because they do not have alternatives to staying in hospitals. Furthermore, a long-term hospital stay in tertiary general hospitals reduces the number of available acute care beds, which may prevent many patients who need acute care from receiving proper treatment. Additionally, patients with advanced cancer do not have primary care physicians because they receive chemotherapy and have no choice but to visit emergency rooms (ERs) for small medical concerns, inevitably

causing overcrowding in ERs.

B. Literature Review

○ **The HBMC is cost-effective and improves the quality of life of patients staying at home**

- There is a growing need for continuous medical services with the increase in the ageing population and development of technology. Additionally, the number of patients staying at home who cannot visit medical institutions due to medical and social problems is increasing. Consequently, HBMC services have been developed and implemented for such patients.
- Unlike the existing residential facilities that concurrently provide acute-phase treatments, medical services, and care, such as long-term care hospitals and nursing homes, the HBMC encompasses the characteristics that allow the patient to stay at home and ensures visits by the medical professional at the patient's place of residence to provide medical services.
- The target and scope of the HBMC are increasingly expanding worldwide because the HBMC improves patients' quality of life because they can continue to live at home and lowers the cost because fewer medical facilities are necessary to provide services, such as buildings.

○ **HBMC interventions for non-severely ill patients staying at home reduce the use of medical services and improve their quality of life**

- The participants of studies conducted overseas on HBMC interventions have been patients staying at home who have mobility impairments and require continuous management through primary medical institutions, such as frail older adult patients, patients with complex chronic diseases (i.e., patients with multiple chronic diseases, including dementia, severe mental illness, and diabetes), patients with mobility difficulties, or patients with disabilities.
- The effect of home-based primary care (HBPC) on the improvement of the quality of life of non-severely ill patients staying at home is well known. Continued HBMC interventions for non-severely ill patients residing at home have demonstrated a positive effect by reducing the

use of medical services and total medical costs even when followed up after 18 months.

○ **HBMC interventions that incorporate transitional care are effective for severely ill patients**

- Providing the HBMC as a part of transitional care for patients with high risk for acute exacerbation, such as heart failure and chronic obstructive respiratory disease, discharged from acute-care hospitals positively reduces readmissions and medical costs.
- Providing home-based palliative care to severely ill patients with incurable illnesses, such as cancer, heart failure, and chronic obstructive respiratory disease, and limited life expectancy has been proven to effectively control their symptoms without shortening their life expectancy and improve their health-related quality of life.

3. The aim of the study

This study aims to assess the effects of a home-based medical intervention program on reducing unplanned rehospitalization within 6 months by providing home visits and periodic checkups by the medical staff for patients with advanced cancer with mobility difficulties and their family caregivers.

4. The design of the study

A. The phase of the clinical study

- Phase III

B. Study participants

Participants will include patients with advanced cancer with mobility difficulties and their family caregivers.

5. Inclusion and exclusion criteria

Both the patient and their family caregivers must meet the following criteria to be eligible for participation in this research.

A. Inclusion criteria

1) Patients

(1) A person who has an advanced-stage solid cancer diagnosis (ICD-10 Code C00-C70) and is receiving or planning to receive cancer treatment. (2) A person who meets one of the following conditions: (a) has been evaluated as having Eastern Cooperative Oncology Group (ECOG) performance status 2 or (b) has been evaluated as having ECOG performance status 1 and is aged above 65 years. (3) A person who wishes to stay at home (In South Korea, patients who need supportive care are usually hospitalized in long-term care hospitals due to the national insurance coverage and the lack of supportive care in cancer treatment hospitals. (4) A person who has a family caregiver cohabiting with them or visiting their home three or more times per week. (5) A person who wishes to participate in the research.

2) Caregivers

The following are the inclusion criteria for family caregivers. (1) A family member of the patient ("family" refers to the patient's spouse [including a common-law partner], children and their spouses, siblings and their spouses, and close relatives and their spouses). (2) A person who meets one of the following conditions: (a) lives with the patient (patient's household member), (b) does not live with the patient, but visits the patient's home more than three times per week, (c) wants the patient to stay at home, (d) can communicate with the medical staff via telephone without any difficulties, or (e) wishes to participate in the research.

B. Exclusion criteria

1) Patients

Patients will be excluded from the study if they (1) cannot speak, understand, or read Korean,

(2) are judged by a medical doctor to be unfit to participate in this research due to extremely poor health, (3) reside outside the geographical range that can be visited by the respective medical institution, (4) are already receiving hospice service, or (5) are aged below 19 years.

2) Caregivers

Exclusion criteria for family caregivers are as follows: a person who (1) cannot speak, understand, or read Korean, (2) is judged by a medical doctor to be unfit to participate in this research due to extremely poor health, or (3) are aged below 19 years.

6. Sample size calculation

The number of samples for the home-based program required in this study is based on a dichotomous variable, which is the primary endpoint, to secure enough patients for a stable and sufficient power. The primary endpoint of this study is the unplanned hospitalization within six months following enrollment. This study aims to determine the differences in the effects by comparing the odds ratio based on the proportion of unplanned hospitalizations in the intervention and control groups. Although no previous intervention study with a similar design has been conducted outside of Korea, the difference in pre- and post-intervention changes between the intervention and control groups should be approximately 20% (20% less in the intervention group) based on the literature review. The number of institutions in both groups is three. The parameters will be estimated using a generalized estimation equation (GEE) to compensate for the cluster effects of the study participants. The comparisons between the two groups will be analyzed using a logistic regression model, which is used to compare the effects of dichotomous outcome variables while considering other confounding variables. Therefore, a power of 80%, a significance level of 0.05 to control for type 1 error, and a 20% difference in the number of unplanned hospitalizations by intervention was used to calculate the sample size. For a ratio of 1:1 for participants in each group, the minimal sample size should be 396 patients

in total, including 198 patients for three clusters in the intervention groups and 198 patients for three clusters in the control group (assuming within-cluster coefficient=0.015).

Within-cluster coefficient (Inraclass correlation coefficient, ICC) was estimated using the analysis of variance (ANOVA) estimation method according to similar previous studies. The calculation formula is as follows (Sheng et al., 2012; Chen et al., 2015)

In the formula, $n_A = \frac{1}{k-1}\left(N - \sum n_i^2/N\right)$ $MSB = \frac{1}{k-1}\left(\sum Z_i^2/n_i - (\sum Z_i)^2/N\right)$ $MSW = \frac{1}{N-k}\left(\sum Z_i - \sum Z_i^2/n_i\right)$ k is the number of cluster and N is the total number of study participants. n_i and Z_i are the number of participants in group i and number of patients with endpoints in group i, respectively. Incidence rates by group were extracted from a study by Chen et al. (2015) and applied in the present study.

k	N	n_1, n_2	Z_1, Z_2	n_A	MSB	MSW	ICC
6	396	198,198	40,20	39.6	0.20202	0.127946	>0.01441

Power	Sample size	# of cluster	Allocation	Average Cluster size	Effect size	ICC	Alpha
0.72584	264	4	ri(1)	66	0.2	0.01	0.05
0.66263	264	4	ri(1)	66	0.2	0.015	0.05
0.5826	264	4	ri(1)	66	0.2	0.02	0.05
0.72584	264	4	ri(1)	66	0.2	0.01	0.05
0.83007	396	6	ri(1)	66	0.2	0.015	0.05
0.75675	396	6	ri(1)	66	0.2	0.02	0.05
0.88014	396	6	ri(1)	66	0.2	0.01	0.05
0.83007	396	6	ri(1)	66	0.2	0.015	0.05
0.8658	528	8	ri(1)	66	0.2	0.02	0.05
0.95162	528	8	ri(1)	66	0.2	0.01	0.05
0.92004	528	8	ri(1)	66	0.2	0.015	0.05
0.92905	660	10	ri(1)	66	0.2	0.02	0.05

ICC: Intraclass correlation coefficient
ri(1): The ratio of allocation for the intervention and control groups = 1:1

- Assuming a maximum dropout rate of 15% in each institution, the final number of enrolled study participants should be 57 for each institution, totaling 342 participants. The expected minimum power is 78.8% (PASS 2022, v22.0.2)
- Three institutions will participate in the intervention group (Seoul National University Hospital, Chung-Ang University Hospital, and Dongguk University Ilsan Hospital) and control group (Kyunghee University Hospital, Bundang CHA Hospital, and Bundang Seoul University Hospital). With a total of 396 expected participants, there will be sufficient power to test the differences in the effects.

7. Study period

Approximately 3.6 years (Recruitment – 2 years; Intervention and assessment – 1.6 years)

A. Recruitment

Approximately 2 years (June 2022 ~ May 2024)

B. Intervention

One year after the enrollment

C. Follow-up period

About 1.6 years (18 months) from the enrollment (including the intervention period)

9. Study participants' enrollment and initial assessment

A. Patient's Registration for the Clinical Trial and Initial Assessment

1) Patient Recruitment

- Post the recruitment information in the outpatient and inpatient wards and public bulletin boards of the participating institutions. Explain the study to the patients and their guardians who meet the inclusion criteria in the outpatient or inpatient ward of co-investigators and

register consenting patients.

- Explain the study to inpatients, outpatients, and their guardians who meet all inclusion criteria and none of the exclusion criteria at each participating institution. Obtain written informed consent from the patients who agree to participate in the study. Similarly, obtain separate written informed consent from their guardians.
- The patient's unique identification information (i.e., resident registration number) will be collected on the consent form for participation in the clinical trial to collect secondary data. Patients will check a separate box for providing their consent to allow the access and use of their data (e.g., data from the National Health Insurance Service and Health Insurance Review and Assessment Service, Statistic Korea's data on death, and data from the Korea National Institute for Bioethics Policy).

2) Initial assessment and education for patients and their family caregivers

An initial assessment will be conducted at the time of patient enrollment. Nurses will assess patients regarding their demographic information, past medical history, current medical condition, QoL, symptoms, emotional status, care burden, care ability, pharmaceutical history, symptom control status, and medical device usage. Patients will receive educational materials and have a one-on-one education session on symptom control (15 min), medication counseling, medical device management, and critical symptoms that must be reported to nurses.

- The research nurse will provide home care education materials (to be developed) at the initial assessment that will include the following contents.

Category	Content
Pain Management	Types of analgesics and the methods of administration
Symptom Management	Types of symptoms that patients may experience and ways to control them at home
Home Care	Ways to manage the home care environment

B. Intervention

1) Multidisciplinary team

The institutions in the intervention group will establish home-based medical teams comprising doctors, nurses, and social workers. The doctors will be home-based care specialists and/or hematologist-oncologists. The nurses will be dedicated home-based medical intervention nurses who would be newly recruited and receive home-based medical intervention training for more than 3 months. Professional medical staff will provide the educational program, consisting of lectures to understand palliative care and symptoms of patients with advanced cancer and HBMC program simulation training.

2) Additional assessment and education

An initial assessment will be conducted at the time of patient enrollment. Nurses will assess patients regarding their demographic information, past medical history, current medical condition, QoL, symptoms, emotional status, care burden, care ability, pharmaceutical history, symptom control status, and medical device usage. Patients will receive the educational materials and have a one-on-one education session on symptom control (15 min), medication counseling, medical device management, and critical symptoms that must be reported to nurses.

Category	Content
Symptom Management	Types of symptoms that patients may experience and ways to control them at home
Medication Administration	Types of medication prescribed to patients and the methods of administration
Medical Device Management Methods	Methods to manage medical devices in case a patient possesses a device
Items that must be Reported to a Healthcare Professional	Types and intensity of symptoms that must be reported to a healthcare professional

3) Home visits by nurses

Nurses in the team will visit the enrolled patient's home within 2 weeks of the initial

assessment. They will make an appointment for a home visit when both the patient and their family caregiver are available. Next, they will assess the home environment for the patient's care, medication, and medical device management. Patients will be educated regarding the condition of their home environment, fall prevention, symptom management, medication counseling, medical device management, and critical symptoms that need to be reported to nurses.

- The medical staff on the HBMC team will evaluate the following items at patients' homes.

Category	Content
Home Care Environment	Risk factors in patients' homes (e.g., rooms, living rooms, and bathrooms); potential risk of falls; proper management of medications and medical devices
Medication Administration	Types of medication a patient has or is currently taking along with the medications prescribed by the institution and the methods of administration
Medical Device Management Status	Whether patients are using medical devices appropriately at home in case they possess a device
Symptoms and Dietary Intake	Assess the symptoms reported by the patient and their dietary intake
Performance Status	ECOG performance status, CFS score
Vital Signs	Assessment of blood pressure, pulse rate, respiratory rate, body temperature, and oxygen saturation after the registration at the first visit

- The medical staff on the HBMC team will evaluate the following items at patients' homes. If adjustments to the home care environment besides the content covered in the initial training are necessary, then education on the relevant content will be provided.

Category	Content
Adjustment of Home Care Environment	Discuss methods of adjusting the home care environment if a risk factor is identified in a patient's home<br>- E.g., installing non-slip mattresses and power generators for emergency oxygen systems at home
Symptom Management	Types of symptoms that patients may experience and ways to control them at home
Medication Administration	Types of medication prescribed to patients and the methods of administration
Medical Device Management Methods	Methods to manage medical devices in case a patient possesses a device
Items that must be Reported to a Healthcare Professional	Types and intensity of symptoms that must be reported to a healthcare professional

4) Monthly multidisciplinary team meeting

A multidisciplinary team meeting of specialized home-based medical teams will be organized once a month. Team meeting agendas are as follows: sharing information on patients enrolled in the study, establishing a care plan for the participants, and regularly reassessing the patients to adjust the care plan. The care plan includes the objectives of home care based on the patient's assessment, symptom management, additional medical needs at home, and reported critical symptoms.

- The care plans established and periodically modified in the multidisciplinary team meetings will include the following.

Category	Content
Care Goal Setting	Establish care goals at the patient's home based on the assessment results of the patient and guardian (i.e., caregiver).<br>- Determine the duration for which the patient wishes to stay at home and the circumstances under which the patient wants to be hospitalized<br>- Check the patient's and guardian's intention for life-sustaining treatments
Symptom Management	Present types of symptoms that the patient may experience and ways to handle them at home.<br>1. For symptoms for which the patient has medication, the HBMC team nurse will educate the patient on how to take the medication and observe whether the symptoms improve.<br>2. For newly occurring symptoms or symptoms that are not controlled by taking the existing medications, the HBMC team nurse will contact the HBMC team physician to discuss whether or not additional medications should be prescribed.<br>3. When a consultation with a physician is necessary, such as if an additional medication prescription is required, the HBMC team nurse will consult with the HBMC team physician to schedule an outpatient visit and inform the patient and guardian.<br>4. If hospitalization or ER visit is required, the HBMC team nurse will contact the patient and their caregiver to inform them.
Home Care Management Plan	Determine whether additional management or visits are required besides biweekly routine management.<br>- A HBMC team nurse will make additional visits to provide training if additional training is required regarding items included in the education, such as symptom management and medication administration, or if the patient or guardian wishes additional visits.<br>- Patients at high risk for worsening symptoms will be contacted within a week after establishing a care plan to identify changes in their condition.
Items that must be Reported to a Healthcare Professional	A HBMC team nurse will share any symptoms or possible incidents that require reporting to the HBMC team physician throughout the routine management period. Commonly included items are:<br>- Pain of numerous ration scale (NRS) score of 7 or higher persists while on

	medication<br>- Uncontrolled symptoms persist while on medication<br>- Occurrence of issues in the use of a medical device that are difficult for a nurse to evaluate or train<br>- Changes in the care provider's care environment (such as difficulty in providing continuous care)

5) Home Care Management

The nurses will contact the patients biweekly via telephone and assess the status of their symptoms. The assessment will include their symptom status, medication adherence, medical device management, home environment for caring, and any requirements of patients and family caregivers. The team doctor will review the assessment, and if necessary, decide to provide medication/medical device adjustment, education, and guidance on visiting the ER. If the patient assessment is insufficient via telephone or in-person patient education is required, the nurses will visit the patient's home.

- A nurse on the HBMC team will evaluate the following items at patients' homes during the routine home care management check.

Category	Content
Performance Status	ECOG performance status, CFS score
Symptom Management	Presence or absence of symptoms, exacerbation, or alleviation compared to previous assessments
Medication Administration	Check whether medications are being taken as prescribed<br>If not, then identify the reason(s)
Medical Device Status	Ensure that medical devices are operating without abnormalities if the patient possesses a device
Miscellaneous	Check for anything the patient and guardian wish to consult with the medical staff

A daytime telephone number to contact the home care medical team nurse between 9 AM to 5 PM on weekdays will be provided to the study participants. If they have any questions or concerns, they can leave a message for the nurse via an access line. The nurse will call the patient and their caregiver to consult about their symptoms, events, or any concerns about the patient's status.

A multidisciplinary team meeting of specialized home-based medical teams will be organized once a month. Team meeting agendas are as follows: sharing information on patients enrolled in

the study, establishing a care plan for participants, and regularly reassessing the patients to adjust their care plan. The care plan will include the objectives of home care based on the patient's assessment, symptom management, additional medical needs at home, and reported critical symptoms.

2. Control

Patients enrolled in the control cluster (institution) will receive educational materials. These patients will continue to receive the usual oncology care.

- If a patient in the control group moves to an intervention group institution for the follow-up, the patient will be excluded from the clinical trial.

11. Outcome measurement

A. Questionnaire and interview

Participants in the intervention and control groups will be interviewed by the researchers at the time of enrollment. Additionally, they will be assessed using a questionnaire at the time of enrollment, three months, and six months. The questionnaire will be administered when the patients and caregivers will visit the institutions for outpatient treatment or hospitalization within one month before or after the time of enrollment. The research nurse will visit the outpatient or inpatient ward to directly evaluate the patients. If the patient and guardian do not visit the hospital due to the absence of outpatient or hospitalization schedules within one month before or after the time of enrollment, the nurse will contact the patient and guardian by phone or email to complete the questionnaire.

Item	Enrollment	3m	6m	12m	Evaluation Method

Demographic characteristics<br>Patient: age, sex, residence, medical security, education, marital status, religion, employment, monthly income, welfare service, disability type, private insurance, household characteristics, family and household composition, care provision status<br>Guardian: age, sex, relationship with the patient, co-residence with the patient, education, marital status, religion, employment, patient care experience	⊙				Researcher interview<br>Patient questionnaire
Patient's general condition.	⊙	⊙	⊙	⊙	Researcher interview
Medical equipment in possession	⊙				Researcher interview
Symptoms and dietary intake	●				
Drug evaluation	●				
Patient's quality of life (EQ-5D)	⊙	⊙	●		Patient questionnaire
Patient's symptoms (ESAS)	⊙	⊙	●		Patient questionnaire
Patient's emotional status (PHQ-9)	⊙	⊙	●		Patient questionnaire
Patient's satisfaction with the service		●	●		Patient questionnaire
Caregiver's quality of life (EQ-5D)	⊙	⊙	●		Patient questionnaire
Caregiver's emotional status (PHQ-9)	⊙	⊙	●		Patient questionnaire
Caregiver's burden of care (CRA-K)	⊙	⊙	●		Patient questionnaire
Caregiver's preparation for care	⊙	⊙	●		
Caregiver's competence	⊙	⊙	●		
Caregiver's sense of accomplishment for care	⊙	⊙	●		
Caregiver's satisfaction with the service		●	●		Patient questionnaire
Caregiver's indirect medical expenses		⊙	⊙*		Patient questionnaire

⊙ Both intervention/control groups, ● Only intervention group
*Conducted in the control group only when consent is provided at 3 months after enrollment for evaluation at 6 months.

B. Medical Record Investigation

- A research nurse will investigate the medical history (e.g., diabetes, hypertension, hyperlipidemia, heart disease, stroke, cerebral hemorrhage, chronic liver disease, chronic kidney disease, asthma, chronic obstructive pulmonary disease, musculoskeletal disease, digestive disease, neurological disease, and psychiatric disease), cancer diagnosis (e.g., carcinoma ICD-10 code and date of diagnosis), cancer treatment history, and plan for life-sustaining treatment at the respective medical institution at the time of patient registration.
- A research nurse will conduct the medical record survey of the respective medical institution at

3, 6, and 12 months of patient enrollment to investigate the status of cancer treatment, hospitalization, ER use, and the use of medical services by the severely ill patient from the time of the previous assessment to each time point, as well as the use of medical services one month prior to death in the case of a deceased patient. The following items will be investigated through medical record surveys.

	3 Months	6 Months	12 Months
Cancer Treatment Status<br>Cancer Progression<br>Anti-cancer Treatment Plan<br>Other Treatment Plans	O	O	O
Hospitalization at a Medical Institution<br>Date of Hospitalization and Discharge<br>Hospitalization Route (e.g., outpatient or ER)<br>Scheduled Hospitalization<br>Result of Hospitalization (e.g., discharge, transfer, or death)	O	O	O
Use of the ER<br>Date of Use (month and year)<br>KTAS at the ER<br>ER Address<br>Result of the Use (e.g., discharge, transfer, or death)	O	O	O
Critical Care Use<br>Date of ICU Admission<br>Date of Ventilator Support<br>Number of Cardiopulmonary Resuscitations	O	O	O
Death in Hospital<br>Date of Death<br>Place of Death (e.g., ward, ICU, or ER)<br>Cause of Death (based on the death certificate)	O	O	O
Medical Service Use 1 Month Before Death<br>Date of ICU Admission<br>Date of Ventilator Support<br>Number of Cardiopulmonary Resuscitations<br>Undergoing Anti-cancer Treatment	O	O	O

C. Secondary linkage data

Concerning patients in the intervention group who agree to provide secondary data linkage,

their National Health Insurance Service data will be accessed after more than 12 months of enrollment (Table 1). The relevant data will be used to investigate information on healthcare service use and direct medical cost up to 12 months after their enrollment. Data will be evaluated at 12 months to recalculate and utilize medical use and cost information at 3, 6, and 12 months. At 18 or more months after enrollment, all patients in the intervention study who provide consent for data sharing will be investigated for information on death, hospice use, healthcare service use before death, and advance care planning status. Table 1 shows the items to be investigated from the National Health Insurance Service data.

Secondary data linkage plan for primary and secondary outcomes

Item	Outcome measurement timing (from the time of enrollment)			
	3 months	6 months	12 months	18 months
Hospitalization<br>Hospitalization date, discharge date<br>Hospitalization route (outpatient or emergency room)<br>Hospitalization results (discharge or death)	○	○	○	
Emergency room use<br>Date (year, month, and date)<br>Result (discharge, hospitalization, or death)	○	○	○	
Critical care use<br>Date of ICU hospitalization (day)<br>Date of ventilator support (day)<br>Number of CPR attempts	○	○	○	○
Hospice use<br>Hospitalization date, discharge date	○	○	○	○
Death<br>Vital status<br>Date of death<br>Cause of death	○	○	○	○
Aggressive care use one month before death<br>Date of ICU hospitalization (day)<br>Date of ventilator support (day)<br>Number of CPR attempts	○	○	○	○
Direct medical expenses (KRW)	○	○	○	
Advance care planning status<br>Advance directives<br>Physician orders for life-sustaining treatment			○	○

12. Efficacy Endpoint Evaluation Criteria, Methods, and Interpretation

A. Evaluation Criteria

1) Primary Efficacy Endpoint

- The proportion of patients with unscheduled hospitalization within 6 months of study enrollment
- Definition of an unscheduled hospitalization: hospitalization at a respective participating institution for acute treatments (excluding scheduled hospitalizations for anti-cancer treatments, operations, and procedures), hospitalization at other medical institutions other than the participating institutions, or **hospitalization of 4 weeks or less at a nursing hospital**

2) Secondary Efficacy Endpoint

- Domain 1: The secondary endpoints related to patients' use of medical services and critical care are described below.
  - Number of unscheduled hospitalizations within 6 months of study enrollment (continuous or count variable)
  - Length of hospitalization (total length of hospitalization; continuous variable)
  - History of hospitalization (dichotomous variable)
  - Number of ER visits (continuous variable)
  - Length of ICU hospitalization (total length of hospitalization; continuous variable)

- Domain 2: The secondary endpoints related to patients' quality of life and their satisfaction with the service are described below.
  - Patient's quality of life (EQ-5D; continuous variable)
  - Patient's degree of symptom control (ESAS; continuous variable)
  - Patient's emotional status (PHQ-9; continuous variable)

- Patient’s satisfaction with the service (continuous variable)

- Domain 3: The secondary endpoints related to the patient’s death and preparation of advanced directives are described below.
  - Survival after enrollment (days; continuous variable)
  - Length of ICU hospitalization within 1 month of the patient’s death (total length of hospitalization; continuous variable)
  - History of anti-cancer treatment within 1 month of the patient’s death (dichotomous variable)
  - Duration of ventilator support within 1 month of the patient’s death (total number of days; continuous variable)
  - Preparation of advance directives or physician orders for life-sustaining treatment (dichotomous variable)

- Domain 4: The secondary endpoints related to guardians’ quality of life and their satisfaction with the service are described below.
  - Guardian’s quality of life (EQ-5D; continuous variable)
  - Guardian’s emotional status (PHQ-9; continuous variable)
  - Guardian’s burden of care (CRA_K; continuous variable)
  - Guardian’s care competency (continuous variable)
  - Guardian’s satisfaction with the service (continuous variable)

- Domain 5: The secondary endpoints related to medical expenses are described below.
  - Direct medical expenses (continuous variable; KRW)
  - Indirect medical expenses (continuous variable; KRW)

B. Evaluation Methods

1) Primary Efficacy Endpoint

- The mean proportion of unscheduled hospitalizations in the intervention and control groups will be compared.

2) Secondary Efficacy Endpoint

- For each item in Domains 1 through 5, the means of continuous variables of the intervention and control groups will be compared. Additionally, the proportions of dichotomous variables of the intervention and control groups will be compared.
- Post-enrollment duration of survival in the intervention and control groups will be compared using the Kaplan-Meier curve.

C. Evaluation Interpretation

1) Primary Efficacy Endpoint

- When the means of the continuous variables in the intervention group are 20% less than the means in the control group, it will imply that the HBMC intervention effectively reduced unplanned hospitalizations within 6 months of provision.

2) Secondary Efficacy Endpoint

- When the means of the continuous variables in the intervention and control groups differ at a significance level of 0.05 or less, it will imply that the HBMC intervention was effective for the secondary endpoint.

13. Suspension and Dropout Procedures

A. Clinical Trial Suspension Criteria

- The clinical trial will be suspended in the following cases.

1. When a patient or their caregiver wishes to discontinue the HBMC intervention
2. When a participant passes away
3. When a participant is hospitalized at a medical institution for an additional four weeks
4. When a patient's treatment at the hemato-oncology department of the respective hospital has been terminated or follow-up has been discontinued
5. When a participant uses an inpatient or home-based hospice care services
6. When the suspension of participation in the clinical trial is deemed best for the participant at the investigator's discretion
7. When a participant does not cooperate with the physician in charge of the clinical trial or does not follow the physician's instructions
8. When the regulatory authority, ethics committee, or Institutional Review Board (IRB) suspends the clinical trial

B. Clinical Trial Suspension Procedures

- A suspension of participation in the HBMC intervention group cannot be resumed.
- Once the suspension of participation in the clinical trial has been decided, the participants (i.e., the patient and guardian) will be asked whether they agree to proceed with the additional survey, except when the suspension is due to death.

C. Follow-up of Suspended Participants

- Investigation of the medical records and secondary data linkage will be performed on the suspended participants from the time of suspension to the end of the remaining scheduled follow-up period (18 months after the registration) until no longer possible due to the participant's death, failure to follow-up, or withdrawal of consent. Any case of a patient receiving additional HBMC services (e.g., other HBMC pilot projects) other than the one provided in this clinical trial after the completion of the study will be recorded in the care

reports.

- For participants suspended due to reasons other than death, the follow-up survey will only be conducted with those who consent to participate via a telephone call made by a research nurse.

D. Participant Dropout Procedures

- Study participants are free to discontinue participation in the clinical trial (the provision of the HBMC intervention and evaluation) at any time (withdrawal of consent). The reasons for the dropout will always be confirmed with these participants. If possible, the investigator will conduct a participant interview and perform a scheduled post-trial assessment and procedures.
- In the case of dropout patients, their previously collected data will be discarded if they do not wish the data to be used in the study.

14. Statistical analysis

A. Hypothesis

The intervention group will show a 20% decrease in unplanned hospitalization than the control group.

B. Outcome variables

1) Primary outcome measures

Unplanned hospitalization within 6 months following enrollment

2) Secondary outcome measures

Patients' medical service utilization, hospitalization, ER visits, healthcare use, end-of-life care (intensive care unit admission, ventilator treatment, and an advance directive), quality of life (modified Edmonton Symptom Assessment System [ESAS] and Euro-Quality of Life-5 Dimension [EQ-5D]), depressive symptom (patient health questionnaire-9 [PHQ-9]), caregivers'

quality of life and satisfaction with the services, and medical expenses.

3) Study group

Intervention and control groups

4) Statistical analysis

Definition of home-based medical service intervention group and study dropouts: Patients who provided informed consent to the selected home-based medical service intervention institutions and control group institutions are defined as the intervention group and control group, respectively. Based on the principle of group classification by intention-to-treat (ITT) that only considers the initial classification of intervention, omission of home-based medical service is still defined as the intervention group, which causes a bias that reduces the effects in the treatment group. Despite the study's design to conservatively estimate the effects in the treatment group, if significant differences are observed in the results among patients who withdraw consent or dropout during follow-up, data up to the point of withdrawal of consent or dropout will be used. Dropout rates will be analyzed and reported for each group.

The mean proportion of unplanned hospitalization will be compared between the intervention and control groups for the primary outcome. The mean of continuous variables and a fraction of dichotomous variables for each item in domains one to five will be compared between the two groups for the secondary outcome. A generalized estimation equation will be used to evaluate intra-group correlations to compensate for clusters of patients in the participating institutions. Survival after enrollment will be compared using the Kaplan-Meier curve and Cox proportional hazard model.

15. Safety Evaluation Criteria, Methods, and Reporting

- The intervention is expected to have no side effects or risks as the participants assigned to the intervention group in this clinical trial are provided with the HBMC program encompassing

education and counseling services Participants assigned to the control group will receive the existing medical services that are commonly provided and are expected to experience no side effects or risks from participating in the study.

16. Victim Compensation Regulations and Participant Protection Measures

A. Ethical Considerations

1) Institutional Review Board

- The investigator will obtain written approval from the IRB prior to the start of the study for the clinical trial protocol, participant information letter and consent form, participant recruitment material and procedures (e.g., advertisement), and all written documents provided to the participants. The investigator will provide updates and other information (e.g., rapid safety reporting, amendments, and administrative correspondence) that should be reported to the IRB according to the procedures of the institutions holding the clinical trial.

2) Ethical Research Conduct

- This clinical trial will be conducted in compliance with the Declaration of Helsinki, Korean national laws, and related regulations. The study will be conducted as per the study protocol. Approval will be obtained from the IRB for the protocol, protocol amendments, and informed consent before the start of the study.
- Investigators involved in this clinical trial will be appropriately qualified to perform their tasks through education, training, and experience.
- Investigators who have been sanctioned or associated with scientific fraud or deception (e.g., loss of medical license and sanctions) will not participate in conducting this clinical trial.
- Procedures and systems will be implemented to ensure quality in all aspects of this study.

3) Participant Information and Consent

- Informed consent will be obtained from all participants before any procedures related to the clinical trial are performed. Approval for the participant information letter and informed consent form will be obtained from the IRB.
- The principal investigator (or delegator) will provide sufficient explanation of the study to the participants. Participants will provide voluntary consent, and a signed copy of the informed consent form will be obtained. Participants may withdraw their consent anytime for any reason during the clinical trial.
- Apart from the consent form for participation in this clinical trial, those who agree to the third-party provision of data and its use in secondary studies other than for the purpose of the current study after the completion of this clinical trial will sign a separate consent form, which will be kept in a separate file.

4) Damage Compensation

- Participation in this clinical trial is expected to pose no risk of physical or psychological harm or specific damage to the participants because the intervention group will receive education and counseling and the control group will use conventional medical services.
- In the event of unforeseen psychological or mental harm due to added procedures and interventions as part of the clinical trial outside the existing course of treatment, the best possible measures will be taken to ensure that the study participants receive appropriate treatment and they will be compensated if there is a reasonable causal relationship between the clinical trial and damage.

B. Aspects of Practice

1) Adherence to Protocol and Protocol Amendments

- This clinical trial will be conducted as specified in the approved protocol. The investigator will

not deviate from or alter the protocol without prior IRB review and written approval for the modifications. However, there can be an exception when it is necessary to eliminate direct risk to participants.

2) Record Keeping

- The investigator will maintain records of the provision of the HBMC program, copy of case reports (or an electronic file), and supporting documents for the period specified by the sponsor and under applicable laws and guidelines

3) Case Report Form

- The investigator will prepare and maintain appropriate and accurate case history records designed to record all observations and other data related to the study obtained during the provision of the HBMC intervention program. Since the data reported in the case report form are based on supporting documents, case report forms should be consistent with the supporting document, and any inconsistencies will be explained.
- The confidentiality of personally identifiable information of the study participants will be respected and protected in compliance with applicable regulatory requirements.
- The investigator will keep a signature document to record the signature and initials of all persons authorized to input data in the case report forms or amend them.
- The investigator will maintain a copy of the case report forms, including records of modifications and amendments.

17. References

Brumley, R. D., Enguidanos, S., & Cherin, D. A. (2003). Effectiveness of a home-based palliative care program for end-of-life. Journal of Palliative Medicine, 6(5), 715–724. doi: 10.1089/109662103322515220. PMID: 14622451.

Brumley, R., Enguidanos, S., Jamison, P., Seitz, R., Morgenstern, N., Saito, S., … & Gonzalez, J. (2007). Increased satisfaction with care and lower costs: Results of a randomized trial of in-home palliative care. Journal of the American Geriatric Society, 55(7), 993–1000. doi: 10.1111/j.1532-5415.2007.01234.x. PMID: 17608870.

Chen, C. Y., Thorsteinsdottir, B., Cha, S. S., Hanson, G. J., Peterson, S. M., Rahman, P. A., … & Takahashi, P. Y. (2015). Health care outcomes and advance care planning in older adults who receive home-based palliative care: A pilot cohort study. Journal of Palliative Medicine, 18(1), 38–44. doi: 10.1089/jpm.2014.0150. PMID: 25375663. PMCID: PMC4273188.

Brian Cassel, J., Kerr, K. M., McClish, D. K., Skoro, N., Johnson, S., Wanke, C., & Hoefer, D. (2016). Effect of a home-based palliative care program on healthcare use and costs. Journal of the American Geriatrics Society, 64(11), 2288–2295. doi: 10.1111/jgs.14354. PMID: 27590922. PMCID: PMC5118096.

Lustbader, D., Mudra, M., Romano, C., Lukoski, E., Chang, A., Mittelberger, J., … & Cooper, D. (2017). The impact of a home-based palliative care program in an accountable care organization. Journal of Palliative Medicine, 20(1), 23–28. doi: 10.1089/jpm.2016.0265. PMID: 27574868. PMCID: PMC5178024.

Pouliot, K., Weisse, C. S., Pratt, D. S., & DiSorbo, P. (2017). First-year analysis of a new, home-based palliative care program offered jointly by a community hospital and local visiting nurse service. The American Journal of Hospice and Palliative Care, 34(2), 166–172. doi: 10.1177/1049909115617139. PMID: 26656032.

Dhollander, N., Smets, T., De Vleminck, A., Van Belle, S., Deliens, L., & Pardon, K. (2019).

Phase 0-1 early palliative home care cancer treatment intervention study. BMJ Supportive and Palliative Care, 12(e1), e103–e111. PMID: 31068333.

Rich, M. W., Beckham, V., Wittenberg, C., Leven, C. L., Freedland, K. E., & Carney, R. M. (1995). A multidisciplinary intervention to prevent the readmission of elderly patients with congestive heart failure. New England Journal of Medicine, 333(18), 1190–1195.

Stewart, S., Pearson, S., & Horowitz, J. D. (1998). Effects of a home-based intervention among patients with congestive heart failure discharged from acute hospital care. Archives of Internal Medicine, 158(10), 1067–1072.

Stewart, S., Vandenbroek, A. J., Pearson, S., & Horowitz, J. D. (1999). Prolonged beneficial effects of a home-based intervention on unplanned readmissions and mortality among patients with congestive heart failure. Archives of Internal Medicine, 159(3), 257–261.

Stewart, S., Marley, J. E., & Horowitz, J. D. (1999). Effects of a multidisciplinary, home-based intervention on planned readmissions and survival among patients with chronic congestive heart failure: A randomized controlled study. The Lancet, 354(9184), 1077–1083.

Stewart, S., Wiley, J. F., Ball, J., Chan, Y. K., Ahamed, Y., Thompson, D. R., & Carrington, M. J. (2016). Impact of nurse-led, multidisciplinary home-based intervention on event-free survival across the spectrum of chronic heart disease: Composite analysis of health outcomes in 1226 patients from 3 randomized trials. Circulation, 133(19), 1867–1877.

Carrington, M. J., Chan, Y. K., Calderone, A., Scuffham, P. A., Esterman, A., Goldstein, S., & Stewart, S. (2013). A multicenter, randomized trial of a nurse-led, home-based intervention for optimal secondary cardiac prevention suggests some benefits for men but not for women: The Young at Heart study. Circulation: Cardiovascular Quality and Outcomes, 6(4), 379–389.

Hermiz, O., Comino, E., Marks, G., Daffurn, K., Wilson, S., & Harris, M. (2002). Randomized controlled trial of home based care of patients with chronic obstructive pulmonary disease. BMJ, 325(7370), 938.

Stewart, S., Pearson, S., Luke, C. G., & Horowitz, J. D. (1998). Effects of home-based

intervention on unplanned readmissions and out-of-hospital deaths. Journal of the American Geriatrics Society, 46(2), 174–180.

Hughes, S. L., Weaver, F. M., Giobbie-Hurder, A., Manheim, L., Henderson, W., Kubal, J. D., ... & Department of Veterans Affairs Cooperative Study Group on Home-Based Primary Care. (2000). Effectiveness of team-managed home-based primary care: A randomized multicenter trial. JAMA, 284(22), 2877–2885.

Cordesse, V., Sidorok, F., Schimmel, P., Holstein, J., & Meininger, V. (2015). Coordinated care affects hospitalization and prognosis in amyotrophic lateral sclerosis: A cohort study. BMC Health Services Research, 15(1), 1–6.

Pozzilli, C., Brunetti, M., Amicosante, A. M. V., Gasperini, C., Ristori, G., Palmisano, L., & Battaglia, M. (2002). Home based management in multiple sclerosis: results of a randomized controlled trial. Journal of Neurology, Neurosurgery, and Psychiatry, 73(3), 250–255.

Pouliot, K., Weisse, C. S., Pratt, D. S., & DiSorbo, P. (2017). First-year analysis of a new, home-based palliative care program offered jointly by a community hospital and local visiting nurse service. American Journal of Hospice and Palliative Medicine, 34(2), 166–172.

Lustbader, D., Mudra, M., Romano, C., Lukoski, E., Chang, A., Mittelberger, J., ... & Cooper, D. (2017). The impact of a home-based palliative care program in an accountable care organization. Journal of Palliative Medicine, 20(1), 23–28.

Malik, A. H., Malik, S. S., Aronow, W. S., & MAGIC (Meta-analysis and Original Investigation in Cardiology) investigators (2019). Effect of home-based follow-up intervention on readmissions and mortality in heart failure patients: A meta-analysis. Future Cardiology, 15(5), 377–386.

Gomes, B., Calanzani, N., Curiale, V., McCrone, P., & Higginson, I. J. (2013). Effectiveness and cost-effectiveness of home palliative care services for adults with advanced illness and their caregivers. Cochrane Database of Systematic Reviews, 6. PMID: 23744578. PMCID: PMC4473359. doi: 10.1002/14651858.CD007760.pub2
