# Supplementary material for: SupporTive Care At Home Research (STAHR) for patients with advanced cancer: Protocol for a cluster non-randomized controlled trial
Source: PLoS One. 2024 May 13;19(5):e0302011. doi: 10.1371/journal.pone.0302011 (PMC11090303; doi:10.1371/journal.pone.0302011)
Supplement: S1 Data — (ZIP) [file pone.0302011.s002.zip › IRB_DUIH_2022-02-013-017_approval_extension_document.doc]

|  | **Notification Letter** |  |
| --- | --- | --- |

| ※ The document retention period of this study is 3 years. | | | | | | | | | |
| --- | --- | --- | --- | --- | --- | --- | --- | --- | --- |
| Recipient | Sponsoring (Supporting) Institution | | National Evidence-based Healthcare Collaborating Agency | | | | | | |
|  | Principal Investigator | | Do Yeon Kim, Department of Internal Medicine | | | | | | |
| IRB File No. | | | DUIH  2022-02-013-020 | Review Content | | Interim Report | | Notification Date | March 2, 2023 |
| Study Title | | Korean | 항암치료를 지속 중인 고형암 환자를 대상으로 재택의료를 제공하여 등록부터 6개월 이내 예정되지 않은 입원 감소 효과를 평가하기 위한 군집 비무작위 배정 연구자 주도 임상시험 | | | | | | |
|  |  | English | A Cluster, Non-randomized Controlled Trial of the Effectiveness of a Korean Model for Home-based Care in Patients with Advanced Cancer | | | | | | |
| Clinical Study Code | | |  | | Study Nick Name | |  | | |

| Study Classification 1 | □ Drug | | | | | □ Biologics | | | | | | □ Cell therapy product | | | | | | | | □ Health supplement food | | | | |
| --- | --- | --- | --- | --- | --- | --- | --- | --- | --- | --- | --- | --- | --- | --- | --- | --- | --- | --- | --- | --- | --- | --- | --- | --- |
|  | □ Medical procedure | | | | | □ Medical device | | | | ( ○ Class 1 | | | | ○ Class 2 | | | | | ○ Class 3 | | | | | ○ Class 4 ) |
|  | ■ N/A | | | | | | | | | | | | | | | | | | | | | | | |
| Study Classification 2 | ■ Research in human | | | | | □ Research on human-derived materials (specimen) | | | | | | | | | | | □ Medical record study | | | | | | | |
|  | □ Genetic study | | | | | □ Gene therapy | | | | | | | | | | | | | | | | | | |
|  | □ Embryo research | | | | | □ Somatic cell cloning embryo research | | | | | | | | | | | □ Stem cell line research | | | | | | | |
|  | □ Other () | | | | | | | | | | | | | | | | | | | | | | | |
| Study Classification 3 | ● Prospective study | | | | | ○ Retrospective study | | | | | | ○ Prospective and retrospective study | | | | | | | | | | | | |
| Study Classification 4 | ■ Intervention study | | | | | □ Questionnaire survey | | | | | | □ Data analysis and analysis study | | | | | | | | | | | | |
|  | □ Observational study | | | | | (□ Cross-sectional study | | | | | | □ Case-control study | | | | | | | | | | | □ Cohort study ) | |
|  | □ Other () | | | | | | | | | | | | | | | | | | | | | | | |
| Study Classification 5 | □ Non-human study/Non-clinical study (in vitro. in vivo preclinical study) | | | | | | | | | | | | | | | | | | | | | | | |
| Generic name |  | | | | | | | | Brand Name | | | | | |  | | | | | | | | | |
| Total Number of Subjects | Total | 396 subjects | | | | | Domestic | | | subjects | | | | | | This Hospital | | | | | | 66 subjects | | |
| Study Approval Period | April 26, 2022 to April 25, 2024 | | | | | | | | | | | | | | | | | | | | | | | |
| Sponsor | Institution Name | | National Evidence-based Healthcare Collaborating Agency | | | | | | Representative (Position) | | | |  | | | | | Name | | | Gwang Hyeop Hahn | | | |
| List of Submitted Documents | (Attachment) Protocol [1.6] [221202]  (Attachment) Subject Information Sheet and Informed Consent Form [1.5] [221027]  (Attachment) Study Checklist [ ] [ ] | | | | | | | | | | | | | | | | | | | | | | | |
| Relevant Basis | Review Date | | | | March 2, 2023 | | | | | | | | | | | | | | | | | | | |
| Interim Report Period | By February 25, 2024 | | | | | | | Remark | | |  | | | | | | | | | | | | | |
| Review Result | ● Approval | | | ○ Conditional approval | | | | | | | | | | | | | | | | | | | | |
| Review Result | As a result of reviewing the study checklist and the submitted document for the study above, it seems that the risk to the subjects has not significantly increased, in comparison to the risk since the initial approval of the study, and we grant “approval.”  ■ Interim report approval date: March 2, 2023  [Notes]  1. The investigator should submit and obtain approval on the continuation review in accordance with the continuation review cycle, and when the study is completed, a close-out report should be submitted within 6 months. | | | | | | | | | | | | | | | | | | | | | | | |

All investigators who have been approved by this Board must comply with the following guidelines:

1. Subjects are prohibited from participating in the corresponding clinical study before obtaining approval for the protocol and protocol amendment.
2. The study must be conducted based on the approved protocol. Conducting the study differently from the original protocol prior to approval of a protocol amendment is prohibited.
3. Please use an informed consent form that has been approved by the IRB.
4. The consent process should be conducted based on a sufficient explanation without coercion or undue influence on the subjects, and the potential subjects should be provided sufficient opportunities to consider their participation in the study.
5. Any change to the study must be made only after prior approval from the Board, unless it is absolutely necessary for the protection of the subjects during the study. Additionally, you must immediately report to the Board on any change made for the protection of the subjects in an emergency situation.
6. If the study needs to be conducted differently from the original protocol to remove an immediate risk factor that has occurred to the subjects, a report must be promptly submitted to the Board on any changes that can increase the risk factor to the subjects or significantly affect the implementation of the study, any suspected unexpected serious adverse drug/medical device reactions, and new information that can adversely affect the safety of the subjects or the implementation of the clinical trial.
7. Subject recruitment advertisements must be used after obtaining approval from the IRB.
8. The approval of the IRB cannot exceed a maximum of 1 year. To continue the study, please submit an application for continuation review according to the continuation review interval requested by the IRB.
9. If the review result is non-approval, a response letter should be submitted within 4 months from the review date.
10. A close-out report and a clinical study report must be submitted upon completion of the study.
11. All investigators must comply with the relevant domestic and overseas laws and regulations, such as the Bioethics and Safety Act, the Pharmaceutical Affairs Act/Medical Devices Act, the Declaration of Helsinki, and the ICH/GCP guidelines.
12. The institution’s internal audit and external inspection may be conducted on the approved study. Whenever access to the study documents (including electronic documents) is requested by the institution’s internal auditor, external monitoring personnel and auditor, regulatory inspector, etc., the study staff must actively cooperate with the request.

►Any board member who has a conflict of interest in this study has been excluded from the review of this study.

►You may check this document for forgery or alteration by scanning the barcode at the bottom of the document.

►This Board complies with the International Conference on Harmonisation-Good Clinical Practice (ICH-GCP).

**Institutional Review Board of Dongguk University Ilsan Hospital** [seal: Seal of the Chairperson of the Institutional Review Board of Dongguk University Ilsan Hospital]
