# Supplementary material for: SupporTive Care At Home Research (STAHR) for patients with advanced cancer: Protocol for a cluster non-randomized controlled trial
Source: PLoS One. 2024 May 13;19(5):e0302011. doi: 10.1371/journal.pone.0302011 (PMC11090303; doi:10.1371/journal.pone.0302011)
Supplement: S1 Data — (ZIP) [file pone.0302011.s002.zip › IRB_KHUH-2022-06-064-013_approval.doc]

| [Logo:] Association for the Accreditation of Human Research Protection Programs, Inc.  Full Accreditation | **Notification Letter** |  |
| --- | --- | --- |

| ※ The document retention period of this study is 3 years. | | | | | | | | | |
| --- | --- | --- | --- | --- | --- | --- | --- | --- | --- |
| Recipient | Sponsoring (Supporting) Institution | | Seoul National University Hospital | | | | | | |
|  | Principal Investigator | | Seon Kyeong Baik, Department of Hemato-oncology | | | | | | |
| IRB File No. | | | KHUH  20220-06-064-003 | Review Content | | Correction Plan | | Notification Date | July 25, 2022 |
| Study Title | | Korean | 항암치료를 지속 중인 고형암 환자를 대상으로 재택의료를 제공하여 등록부터 6개월 이내 예정되지 않은 입원 감소 효과를 평가하기 위한 군집 비무작위 배정 연구자 주도 임상시험 | | | | | | |
|  |  | English | A Cluster, Non-randomized Controlled Trial of the Effectiveness of a Korean Model for Home-based Care in Patients with Advanced Cancer | | | | | | |
| Clinical Study Code | | |  | | Study Nick Name | |  | | |

| Study Classification 1 | □ Drug | | | | | □ Biologics | | | | | | □ Cell therapy product | | | | | | | | □ Health supplement food | | | | |
| --- | --- | --- | --- | --- | --- | --- | --- | --- | --- | --- | --- | --- | --- | --- | --- | --- | --- | --- | --- | --- | --- | --- | --- | --- |
|  | □ Medical procedure | | | | | □ Medical device | | | | ( ○ Class 1 | | | | ○ Class 2 | | | | | ○ Class 3 | | | | | ○ Class 4 ) |
|  | ■ N/A | | | | | | | | | | | | | | | | | | | | | | | |
| Study Classification 2 | ■ Research in human | | | | | □ Research on human-derived materials (specimen) | | | | | | | | | | | □ Medical record study | | | | | | | |
|  | □ Genetic study | | | | | □ Gene therapy | | | | | | | | | | | | | | | | | | |
|  | □ Embryo research | | | | | □ Somatic cell cloning embryo research | | | | | | | | | | | □ Stem cell line research | | | | | | | |
|  | □ Other () | | | | | | | | | | | | | | | | | | | | | | | |
| Study Classification 3 | ● Prospective study | | | | | ○ Retrospective study | | | | | | ○ Prospective and retrospective study | | | | | | | | | | | | |
| Study Classification 4 | □ Intervention study | | | | | ■ Questionnaire survey | | | | | | □ Data analysis and analysis study | | | | | | | | | | | | |
|  | ■ Observational study | | | | | (□ Cross-sectional study | | | | | | □ Case-control study | | | | | | | | | | | ■ Cohort study ) | |
|  | □ Other () | | | | | | | | | | | | | | | | | | | | | | | |
| Study Classification 5 | □ Non-human study/Non-clinical study (in vitro. in vivo preclinical study) | | | | | | | | | | | | | | | | | | | | | | | |
| Generic name |  | | | | | | | | Brand Name | | | | | |  | | | | | | | | | |
| Total Number of Subjects | Total | 396 subjects | | | | | Domestic | | | 396 subjects | | | | | | This Hospital | | | | | | 66 subjects | | |
| Study Approval Effective Period | July 22, 2022 to July 21, 2023 | | | | | | | | | | | | | | | | | | | | | | | |
| Sponsor | Institution Name | | Seoul National University Hospital | | | | | | Representative (Position) | | | |  | | | | | Name | | | Yeon Soo Kim | | | |
| List of Submitted Documents | (Attachment) Protocol [1.3]  (Attachment) Study (Research) Subject Information Sheet and Informed Consent Form (ICF) [1.1]  (Attachment) ICF on the release to 3^rd^ parties and secondary use of study information [1.1]  (Attachment) Response letter to review comments  (Attachment) Protocol agreement  (Attachment) Table of Change | | | | | | | | | | | | | | | | | | | | | | | |
| Relevant Basis | Review Date | | | | July 22, 2022 | | | | | | | | | | | | | | | | | | | |
| Interim Report Period | By July 21, 2023 | | | | | | | Remark | | |  | | | | | | | | | | | | | |
| Review Result | ● Approved | | | ○ Conditional approval | | | | | | | | | | | | | | | | | | | | |
| Review Result | <Response letter to the Board’s review comments>  1) When a dropout rate of 15% is assumed among 198 subjects, the number 172 is not met. Please check the calculation of the number of samples.  [Response] As the previously submitted protocol is the version prior to the approval of the Seoul National University Hospital, we submit the amended version, 1.3ver, which includes the calculation formula for the number of samples.  2) There is an inconsistency in the primary endpoints in the protocol—please revise for consistency. (Page 6: number of unplanned hospitalizations for 6 months after study enrollment, page 22: percentage of patients with unplanned hospitalizations for 6 months after study enrollment).  [Response] Page 6: It has been revised as follows: The number of unplanned hospitalizations for 6 months after study enrollment/Page 23: The number of patients with unplanned hospitalizations for 6 months after study enrollment.  3) Please make revisions by referring to the revised Subject Information Sheet and ICF (including the ICF on the provision to 3^rd^ parties and secondary use of study information) in the attachment file.  [Response] We have revised and attached the Subject Information Sheet and ICF (including the ICF on the provision to 3^rd^ parties and secondary use of study information) in the form of the attachment file.  ※ Please obtain consent from subjects by printing out the ICF with a watermark.  ※ The protocol has been amended from Ver 1.0 -> Ver 1.3 for Seoul National University Hospital.  ※ For inquiries, please contact the administrative secretary: Kyeong Hwa Hahn, Tel: 958-9566, Fax: 958-9559, email: [yynam@khmc.or.kr](mailto:yynam@khmc.or.kr).  ※ This is an expedited review as it fell under the category of not increasing the risk for subjects or reports or amendments with below minimal risk, according to the expedited review checklist.  - Review of protocol submitted after supplementation in accordance with the IRB review outcome (conditional approval)  (Review of the amended protocol as instructed by the IRB review) | | | | | | | | | | | | | | | | | | | | | | | |

- The Institutional Review Board of Kyung Hee University Hospital complies with relevant regulations, such as the International Council for Harmonisation ICH/GCP, Good Clinical Practice (GCP), and the Bioethics and Safety Act.
- Any board member who has a conflict of interest in this study has been excluded from the review of this study.
- The institution name may never be used in connection with this clinical trial result, except for academic purposes, without the prior written consent of the institution.
- You may check this document for forgery or alteration by scanning the barcode at the bottom of the document.

**Chairperson of Kyung Hee University Hospital Institutional Review Board** [seal: Seal of the Chairperson of the Kyung Hee University Hospital Institutional Review Board]
