# Supplementary material for: SupporTive Care At Home Research (STAHR) for patients with advanced cancer: Protocol for a cluster non-randomized controlled trial
Source: PLoS One. 2024 May 13;19(5):e0302011. doi: 10.1371/journal.pone.0302011 (PMC11090303; doi:10.1371/journal.pone.0302011)
Supplement: S1 Data — (ZIP) [file pone.0302011.s002.zip › IRB_KHUH-2022-06-064-013_approval_extension_document.doc]

| [Logo:] Association for the Accreditation of Human Research Protection Programs, Inc.  Full Accreditation | **Notification Letter** |  |
| --- | --- | --- |

| ※ The document retention period of this study is 3 years. | | | | | | | | | |
| --- | --- | --- | --- | --- | --- | --- | --- | --- | --- |
| Recipient | Sponsoring (Supporting) Institution | | Seoul National University Hospital | | | | | | |
|  | Principal Investigator | | Seon Kyeong Baik, Department of Hemato-oncology | | | | | | |
| IRB File No. | | | KHUH  20220-06-064-025 | Review Content | | Interim Report | | Notification Date | August 28, 2023 |
| Study Title | | Korean | 항암치료를 지속 중인 고형암 환자를 대상으로 재택의료를 제공하여 등록부터 6개월 이내 예정되지 않은 입원 감소 효과를 평가하기 위한 군집 비무작위 배정 연구자 주도 임상시험 | | | | | | |
|  |  | English | A Cluster, Non-randomized Controlled Trial of the Effectiveness of a Korean Model for Home-based Care in Patients with Advanced Cancer | | | | | | |
| Clinical Study Code | | |  | | Study Nick Name | |  | | |

| Study Classification 1 | □ Drug | | | | | □ Biologics | | | | | | □ Cell therapy product | | | | | | | | □ Health supplement food | | | | |
| --- | --- | --- | --- | --- | --- | --- | --- | --- | --- | --- | --- | --- | --- | --- | --- | --- | --- | --- | --- | --- | --- | --- | --- | --- |
|  | □ Medical procedure | | | | | □ Medical device | | | | ( ○ Class 1 | | | | ○ Class 2 | | | | | ○ Class 3 | | | | | ○ Class 4 ) |
|  | ■ N/A | | | | | | | | | | | | | | | | | | | | | | | |
| Study Classification 2 | ■ Research in human | | | | | □ Research on human-derived materials (specimen) | | | | | | | | | | | □ Medical record study | | | | | | | |
|  | □ Genetic study | | | | | □ Gene therapy | | | | | | | | | | | | | | | | | | |
|  | □ Embryo research | | | | | □ Somatic cell cloning embryo research | | | | | | | | | | | □ Stem cell line research | | | | | | | |
|  | □ Other () | | | | | | | | | | | | | | | | | | | | | | | |
| Study Classification 3 | ● Prospective study | | | | | ○ Retrospective study | | | | | | ○ Prospective and retrospective study | | | | | | | | | | | | |
| Study Classification 4 | □ Intervention study | | | | | ■ Questionnaire survey | | | | | | □ Data analysis and analysis study | | | | | | | | | | | | |
|  | ■ Observational study | | | | | (□ Cross-sectional study | | | | | | □ Case-control study | | | | | | | | | | | ■ Cohort study ) | |
|  | □ Other () | | | | | | | | | | | | | | | | | | | | | | | |
| Study Classification 5 | □ Non-human study/Non-clinical study (in vitro. in vivo preclinical study) | | | | | | | | | | | | | | | | | | | | | | | |
| Generic name |  | | | | | | | | Brand Name | | | | | |  | | | | | | | | | |
| Total Number of Subjects | Total | 396 subjects | | | | | Domestic | | | 396 subjects | | | | | | This Hospital | | | | | | 66 subjects | | |
| Study Approval Effective Period | August 21, 2023 to August 20, 2024 | | | | | | | | | | | | | | | | | | | | | | | |
| Sponsor | Institution Name | | Seoul National University Hospital | | | | | | Representative (Position) | | | |  | | | | | Name | | | Yeong Tae Kim | | | |
| List of Submitted Documents | - Application for Annual Continuation Review  - Protocol [1.9]  - Protocol Table of Change [1.3] -> [1.4] -> [1.5] -> [1.6] -> [1.9]  - Case Report Form (CRF) [1.7]  - Subject Information Sheet and Informed Consent Form (ICF) [1.1] -> [1.4] -> [1.2] -> [1.3] -> [1.4]  - ICF on the release to the 3^rd^ parties and secondary use of study information [1.5]  - ICF on the release to the 3^rd^ parties and secondary use of study information Table of Change [1.1] -> [1.4] -> [1.5]  - Patient Questionnaire [1.4]  - Caregiver Questionnaire [1.4]  - Statement of Study Budget Calculation (KRW 140,600,000)  - Principal Investigator CV: Prof. Seon Kyeong Baik, Department of Hemato-oncology  - Data and Safety Monitoring Plan [1.2]  - Delegation Signature Log  - Approval Letter from the overseeing institution  - Response material for conditional approval  - Subject progress status list  - Sub-investigator Table of Change | | | | | | | | | | | | | | | | | | | | | | | |
| Relevant Basis | Face-to-face Meeting | | | | August 21, 2023 | | | | | | | | | | | | | | | | | | | |
| Interim Report Period | By August 20, 2024 | | | | | | | Remark | | |  | | | | | | | | | | | | | |
| Review Result | ● Approved | | | ○ Conditional approval ○ Supplement ○ Rejected | | | | | | | | | | | | | | | | | | | | |
| Review Result | As a result of reviewing the submitted documents for the study above, the Board has decided to grant approval for the above application for study review as it is deemed that the risk for subjects is not expected to increase additionally since the initial approval of the study.  ※Interim review cycle: Replaced with an annual review (12 months)  ※In the case of “supplement” or “conditional approval,” which requires supplementation, please submit the supplementary data or the materials on objection (within 3 months from the date of notification) to the IRB. The application will be automatically rejected if the supplementary data or materials on objection are not submitted within the specified timeline. For inquiries, please contact the administrative secretary: Soo Jin Hahn (Tel: 958-9579, Fax: 958-9559, email: [khcri@khmc.or.kr](mailto:khcri@khmc.or.kr)), administrative staff: Ji Eun Shin (Tel: 958-9571, Fax: 958-9559, email: 27459@khmc.or.kr). | | | | | | | | | | | | | | | | | | | | | | | |

- The Institutional Review Board of Kyung Hee University Hospital complies with relevant regulations, such as the International Council for Harmonisation ICH/GCP, Good Clinical Practice (GCP), and the Bioethics and Safety Act.
- Any board member who has a conflict of interest in this study has been excluded from the review of this study.
- The institution name may never be used in connection with this clinical trial result, except for academic purposes, without the prior written consent of the institution.
- You may check this document for forgery or alteration by scanning the barcode at the bottom of the document.

**Chairperson of Kyung Hee University Hospital Institutional Review Board** [seal: Seal of the Chairperson of the Kyung Hee University Hospital Institutional Review Board]
